# Single‐cell transcriptomics of suprachiasmatic nuclei reveal a Prokineticin‐driven circadian network

**DOI:** 10.15252/embj.2021108614

**Published:** 2021-09-06

**Authors:** Emma L Morris, Andrew P Patton, Johanna E Chesham, Alastair Crisp, Antony Adamson, Michael H Hastings

**Affiliations:** ^1^ Division of Neurobiology MRC Laboratory of Molecular Biology Cambridge UK; ^2^ The Genome Editing Unit Faculty of Biology, Medicine and Health University of Manchester Manchester UK

**Keywords:** neural network, neuropeptides, Prokineticin receptor 2, Prokineticin2, single‐cell transcriptomics, Neuroscience, Signal Transduction

## Abstract

Circadian rhythms in mammals are governed by the hypothalamic suprachiasmatic nucleus (SCN), in which 20,000 clock cells are connected together into a powerful time‐keeping network. In the absence of network‐level cellular interactions, the SCN fails as a clock. The topology and specific roles of its distinct cell populations (nodes) that direct network functions are, however, not understood. To characterise its component cells and network structure, we conducted single‐cell sequencing of SCN organotypic slices and identified eleven distinct neuronal sub‐populations across circadian day and night. We defined neuropeptidergic signalling axes between these nodes, and built neuropeptide‐specific network topologies. This revealed their temporal plasticity, being up‐regulated in circadian day. Through intersectional genetics and real‐time imaging, we interrogated the contribution of the Prok2‐ProkR2 neuropeptidergic axis to network‐wide time‐keeping. We showed that Prok2‐ProkR2 signalling acts as a key regulator of SCN period and rhythmicity and contributes to defining the network‐level properties that underpin robust circadian co‐ordination. These results highlight the diverse and distinct contributions of neuropeptide‐modulated communication of temporal information across the SCN.

## Graphical abstract

Eleven distinct neuronal sub‐populations constitute the central circadian clock in the mammalian hypothalamus, and integrate temporal information via diverse and distinct contributions of neuropeptide‐modulated communication.

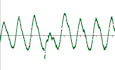

## Introduction

The ca. 20,000 circadian (*circa‐diem*) clock cells of the mammalian suprachiasmatic nucleus (SCN) (Abrahamson & Moore, 2001) co‐ordinate sub‐ordinate cellular clocks distributed across the body and thereby sustain adaptive daily rhythms of physiology and behaviour (Reppert & Weaver, 2002). Individual cells maintain near 24 h autonomous rhythms through self‐sustaining transcriptional/ translational feedback loops (TTFLs) (Partch *et al*, 2014), whereby Period (Per) and Cryptochrome (Cry) proteins negatively regulate their own expression, which is trans‐activated by CLOCK:BMAL1 heterodimers. These cellular TTFLs are coupled and synchronised across the SCN by paracrine and synaptic signals (Yamaguchi *et al*, 2003; Maywood *et al*, 2011; Noguchi *et al*, 2017; Patton *et al*, 2020). This coupling between cells generates emergent network‐level properties of robust and precise oscillation (Hastings *et al*, 2018), which are adjusted to external time via direct retinal innervation (Abrahamson & Moore, 2001). The synchrony, robustness and precision generated by network interactions are necessary to sustain the role of the SCN as principal pacemaker. Indeed, conditional activation of the TTFL of SCN neurons alone not only initiates circadian rhythms in the SCN, but is also sufficient to drive circadian behaviour in an otherwise clockless mouse (Maywood *et al*, 2018). This powerful autonomous time‐keeping of the SCN is evident in its ability to maintain precise network‐level rhythmicity when isolated *in vitro* (Green & Gillette, 1982; Yamaguchi *et al*, 2003). Nevertheless, the cellular, neurochemical and topological bases of the network mechanisms that generate such a reliable oscillator remain unclear.

All SCN neurons express the neurotransmitter γ‐aminobutyric acid (GABA) alongside a range of neuropeptides, and it has been proposed that the GABAergic network is a foundation on which neuropeptidergic signals broadcast information between compartments, i.e. cellular nodes, of the SCN network (Hastings *et al*, 2018). Compartmentalisation of the SCN first highlighted the dichotomy of retinorecipient core and surrounding shell (Abrahamson & Moore, 2001). More recently, however, genetic manipulations have revealed the distinct contributions of cells expressing key neuropeptides: vasoactive intestinal peptide (VIP), arginine vasopressin (AVP) and their cognate receptors (VPAC2 and AVPR1A/B) (Harmar *et al*, 2002; Brown *et al*, 2007, 2017; Yamaguchi *et al*, 2013; Mieda *et al*, 2015), which map onto this core/shell topology. Transcriptomic analysis using single‐cell PCR on 87 candidate genes recently provided greater resolution, highlighting distinct cellular sub‐populations within the neuropeptidergic topology of the SCN (Park *et al*, 2016). Five SCN neuronal subtypes have also been identified by single‐cell RNA sequencing (scRNASeq) of tissue harvested from adult mice, with cluster‐specific marker genes of VIP, AVP, gastrin‐releasing peptide (GRP), cholecystokinin (CCK) and the cell‐adhesion regulator C1ql3 (Wen *et al*, 2020).

The aims of the current study were, first, to conduct a comprehensive and unbiased scRNASeq screen of the SCN to identify cellular components transcriptionally. We used the SCN organotypic slice preparation, which contains the complete apparatus for unperturbed and autonomous molecular time‐keeping. We then used that information to define the topology of neuropeptidergic signalling across circadian time. Finally, having defined cellular nodes within that topology, we aimed to test their circadian role within the network. In doing so, we revealed that the neuropeptidergic signalling axis based on cells expressing Prokineticin2 (Prok2) and its cognate receptor (ProkR2) is a transcriptionally, topologically and functionally distinct pacemaking element of the SCN. Overall, these findings provide a new framework within which to understand the role of intercellular signalling networks, and their transcriptional assembly, in building SCN resilience, and may also inform other settings in which neuropeptidergic cues direct information flow across time and circuit space.

## Results

### ScRNASeq of SCN neurons

**Figure 1:**
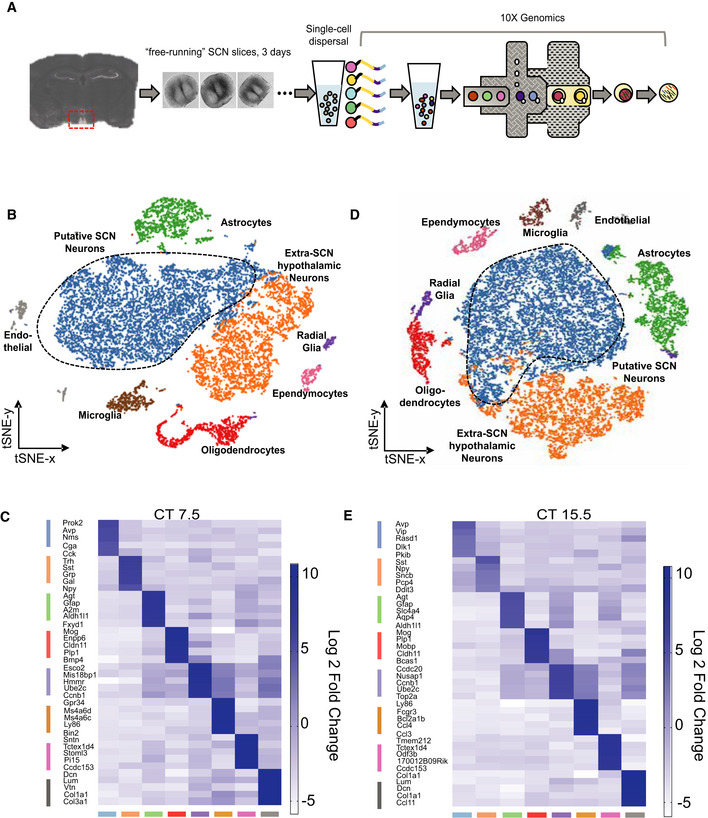
ScRNASeq of mouse SCN slices at two opposing circadian time‐points ASchematic work flow for 10× Genomics scRNASeq of pooled SCN slices after 3 days in culture. For each sequencing run, ca. 17 organotypic SCN slices were pooled into a single sample and their cells dissociated using papain to obtain single‐cell suspension of ˜8,000 cells/µl. Dispersed single cells, along with barcoded 10× Genomics Chromium Single Cell 3′ v2 technology gel beads, were partitioned into water‐in‐oil droplets. Within these droplets, reverse transcription and amplification steps generated cDNA libraries for the barcoded single cells.Bt‐SNE plot from 13,324 sequenced SCN cells collected at CT7.5, across three independent sequencing runs. Each sequenced cell marked with a unique barcode is represented as a single dot. This dimensionality reduction method aims to maintain both local and global structure of the data by clustering data points of highest similarity nearest to each other. Cell clusters recognised by the graph‐based clustering algorithm are further colour‐coded to highlight the cell types: putative SCN neurons, extra‐SCN hypothalamic neurons, astrocytes, oligodendrocytes, radial glia, microglia, ependymocytes and endothelial cells.CHeatmap of the top 5 up‐regulated genes that most distinguish the transcriptional clusters identified in B. The degree of up‐regulation is measured as the log 2‐fold change ratio of gene expression calculated for cells of each cluster and normalised to a size factor accounting for the total transcript count of each sequenced cell across each cluster.Dt‐SNE plot from 16,996 SCN sequenced cells collected at CT15.5, across two independent sequencing runs. As for B, the cell types, putative SCN neurons, extra‐SCN hypothalamic neurons, astrocytes, oligodendrocytes, radial glia, microglia, ependymocytes and endothelial cells, were identified.EAs C, but for cell clusters identified at CT15.5. Source data are available online for this figure.

To obtain a comprehensive transcriptomic dataset of mouse SCN cells, free‐running organotypic SCN slices were collected in subjective day, during peak neuronal activity levels (Patton *et al*, 2020), at circadian time (CT)7.5. This phase was identified by prior recording of Per2::Luciferase bioluminescence rhythms (Appendix Fig S1A). Single‐cell dispersions were made from ca. 17 SCN slices pooled for each of three, independent sequencing runs (52 slices in total), sequencing 13,324 cells (83,220 ± 15,783 reads/cell, mean ± SEM) (Fig 1A). A t‐distributed stochastic neighbour embedding (t‐SNE) plot clustered cells with similar transcriptional characteristics (Fig 1B). This readily identified eight distinct populations, and cluster‐distinguishing genes were used to identify cell types: putative SCN neurons (*Nms* ^+^, *Avp* ^+^), extra‐SCN hypothalamic neurons (*Sst* ^+^, *Gal* ^+^), astrocytes (*Aqp4* ^+^, *Gfap* ^+^), oligodendrocytes (*Mog* ^+^, *Plp1* ^+^), radial glia (*Ccnb1* ^+^, *Ube2c* ^+^), microglia (*Ly86* ^+^, *C1qa* ^+^), ependymocytes (*Tmem212* ^+^, *Tctex1d4* ^+^) and endothelial cells (*Lum* ^+^, *Dcn* ^+^) (Fig 1C). Importantly, the same marker genes describing the same eight clusters were observed for all three, independent circadian daytime sequencing runs. Moreover, the proportion of putative SCN cells within the dataset (50.3 ± 1.7% across the three sequencing runs) was equivalent (unpaired *t*‐test *P* = 0.11) to that expected (41.8 ± 3.8%, *n* = 52), based on the phase‐bright morphology of the SCN in slices (Fig 1A). SCN time‐keeping is under‐pinned by its spontaneously changing transcriptional state across circadian time. We therefore also determined the transcriptome of SCN slices in circadian night, CT15.5 (Appendix Fig S1B), when the activity of SCN neurons enters its quiescent interval (Patton *et al*, 2020). Two independent replications processed a total of 36 SCN slices and sequenced 16,996 cells (43,231 ± 14,291 reads/cell) and identified the same eight cell types (Fig 1D and E). We subsequently focused on SCN neurons.

**Figure 2:**
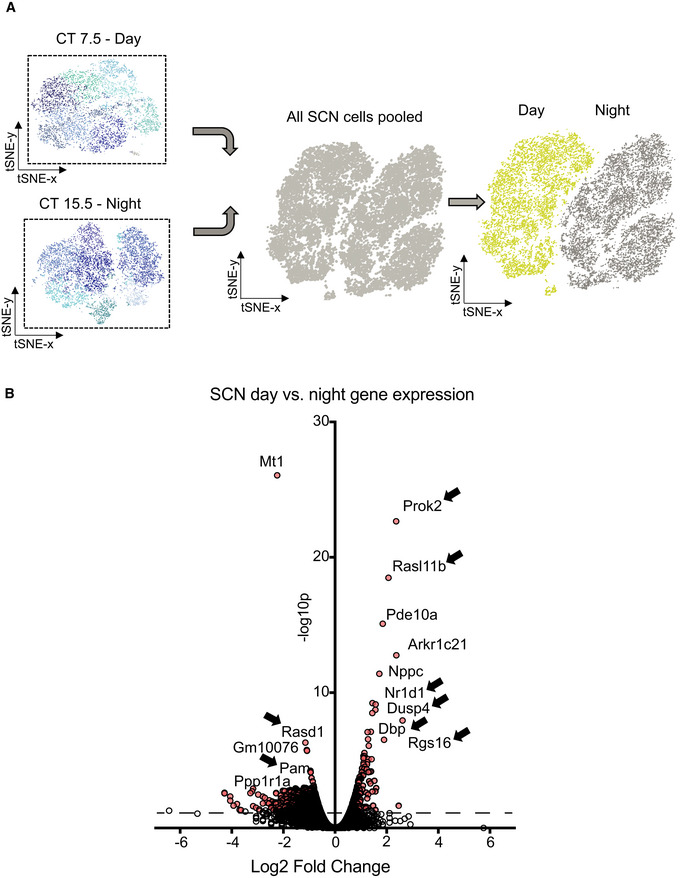
Transcriptional state of SCN neurons at day and night APooled SCN cells generate a combined dataset of 17,363 cells. The sequenced SCN cells carry explicit barcodes, which were used to back‐reference sequencing run identity. This yields a clear‐cut segregation into two transcriptionally distinct cell groups. Left: 98.6% of cells in the yellow cluster are CT7 5 cells. Right: 98.8% of cells in the grey cluster are CT15.5 cells.BVolcano plot showing the magnitude of differential gene up‐regulation, as measured by the respective log 2‐fold change in expression level, at day (positive) or night (negative) versus statistical significance, as quantified by a negative binomial exact test (red dots, dashed line *P* < 0.05 adjusted for multiple testing using Benjamin–Hochberg). Known circadian‐regulated genes are labelled. Source data are available online for this figure.

To obtain a global view of their circadian time‐dependent changes, transcriptomes of all 17,363 SCN neurons were combined and the graph‐based clustering algorithm was set to identify two clusters. This yielded a clear‐cut separation of two transcriptionally distinct clusters (Fig 2A). Back‐reference to cell‐specific barcodes revealed that the two clusters represented either circadian daytime or night‐time samples. Thus, circadian time influences the transcriptional state of SCN neurons to such a degree that the pooled cells segregated almost perfectly (98.8% cluster coverage) into those collected at day or night. We next asked which were the main circadian day‐/night‐defining genes by using a negative binomial exact test (CellRanger in‐built algorithm, multiple testing corrected using Benjamin–Hochberg) (Fig 2B). Known circadian genes such as *Rasd1*, which regulates SCN responsiveness to photic and non‐photic cues (Cheng *et al*, 2004, 2006), and the E3 ligase *Pam*, which is involved in Rev‐Erba degradation (Yin *et al*, 2010), were significantly up‐regulated at night. In contrast, day‐distinguishing SCN genes included *Nr1d1* (encoding Rev‐Erba), *Dbp* and *Rasl11b* whose expression is known to peak during early subjective day (Yamaguchi *et al*, 2000; Gerstner *et al*, 2006). In addition, genes involved in the gating of SCN network signalling such as *Rgs16* (Doi *et al*, 2011) and *Dusp4* (Hamnett *et al*, 2019) were notably up‐regulated in the daytime. Overall, gene ontology identified highly significant up‐regulation of genes associated with synaptic function (including GABA and dopamine) and cell contacts in circadian day, whilst circadian night was characterised by protein degradation and loss of neuropeptide transport (Appendix Fig S2). The most significantly daytime up‐regulated gene (> 5‐fold enriched) was *Prok2*, which encodes the neuropeptide Prokineticin (Prok)2.

**Figure 3:**
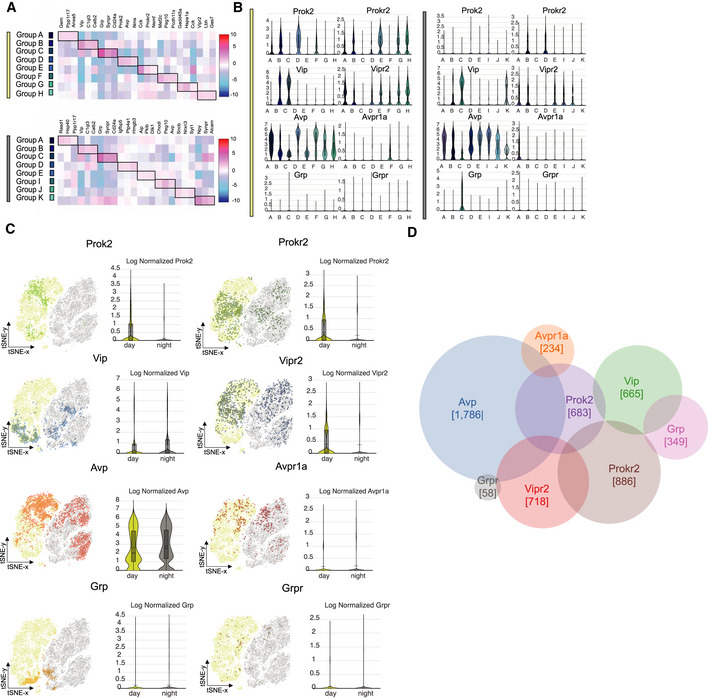
SCN neuronal sub‐population transcriptomes ASub‐clustering of eight neuronal populations using unsupervised graph‐based clustering within the transcriptomes of CT7.5 (top) and CT15.5 (bottom) SCN cells. The heatmaps show the respective up‐regulation of three representative marker genes in each sub‐population. When compared, three sub‐populations showed strong transcriptional similarity between the time‐points of sequencing (Groups A, B and C), two showed partial similarity (Groups D and E), and three were transcriptionally distinct to either CT7.5 or CT15.5 sequenced cells, giving rise to the identification of eleven SCN neuronal sub‐populations.BViolin plots depicting the distribution of the quantified average cellular expression of *Prok2*, *ProkR2*, *Vip*, *Vipr2*, *Avp*, *Avpr1a, Grp* and *Grpr* in each sub‐cluster in CT7.5 (left) and CT15.5 (right) datasets.CVisualisation of the location within the pooled t‐SNE plot of cells positively expressing >90% of the overall expression range of *Prok2*, *ProkR2*, *Vip*, *Vipr2*, *Avp*, *Avpr1a*, *Grp* and *Grpr*. This shows a contrasting expression pattern of the respective genes in cells collected at CT7.5 versus CT15.5.DVenn diagram summarising the degree of non‐/overlapping signalling axes: *Prok2* (purple)*–ProkR2* (brown)*, Vip* (green)*–Vipr2* (red)*, Avp* (blue)*–Avpr1a* (orange) *and Grp* (pink)*–Grpr* (grey). The number SCN neurons collected at CT7.5 positively expressing each gene, which is associated to the respective signalling axis, is further annotated within the population circles. Source data are available online for this figure.

To identify SCN neuronal sub‐populations, we used unsupervised graph‐based clustering of the transcriptomes of the 7,931 daytime SCN neurons. This identified eight clusters for which three marker genes, chosen from the top 12 most up‐regulated genes per cluster, were used as cluster labels: Groups A (*Gem* ^+^/*Ppp1r17* ^+^/*Anxa5* ^+^), B (*Vip* ^+^/*C1ql3* ^+^/*Calb2* ^+^), C (*Grp* ^+^/*Synpr* ^+^/*Cd24a* ^+^), D (*Prok2* ^+^/*Avp* ^+^/*Nms* ^+^), E (*Cck* ^+^/*ProkR2* ^+^/*Nsmf* ^+^), F (*Mef2c* ^+^/*Peg10* ^+^/*Pcdh11x* ^+^), G (*Gadd45a* ^+^/*Hspa1a* ^+^/*Cck* ^+^) and H (*Vipr2* ^+^/*Lbh* ^+^/*Gas7* ^+^) (Fig 3A). For all three sequencing runs, the algorithm consistently found the same marker genes defining the same eight groups. Thus, neuropeptidergic identity based on ligand and/or receptor expression was a commonly distinguishing feature across the sequenced neuronal cell types, and was accompanied by distinct, cluster‐defined transcriptional programmes (or states). Similarly, clustering of the 9,432 circadian night SCN neurons identified eight distinct sub‐populations. Three of these showed strong similarity to daytime Groups A, B and C, whilst two showed partial similarity with CT7.5 Groups D and E, and likely represent the same sub‐populations. Nevertheless, three clusters had transcriptional profiles not evident in circadian day and so were named Groups I, J and K. In the absence of neurogenesis, we interpret these as distinct, newly adopted states of the cells identified as Groups F, G and H in daytime samples. Marker genes for all eight night‐time sub‐populations were identified: Groups A‐C as for CT7.5, Groups D (*Igfbp5* ^+^/*Ptp4a1* ^+^/*Hmgb3* ^+^), E (*Aip* ^+^/*Pkib* ^+^/*Dlk1* ^+^), I (*Chodl* ^+^/*Peg10* ^+^/*Avp* ^+^), J (*Sncb* ^+^/*Nrxn3* ^+^/*Syt1* ^+^) and K (*Alcam* ^+^/*Synpr* ^+^/*Npy* ^+^). Moreover, due to their cluster‐specific enrichment, the expression counts of the eight genes, *Prok2* and *ProkR2*, *Vip* and *Vipr2*, *Avp* and *Avpr1a*, and *Grp* and *Grpr*, provided a common point of comparison between each of the eight individual circadian daytime and/or night‐time 5 clusters (Fig 3B). Cells positively expressing > 90% of the overall expression range of these genes were highlighted within the t‐SNE plot (Fig 3C), from which contrasting expression patterns and levels clearly illustrate their circadian modulation.

### Topology of SCN neuropeptidergic signalling axes

**Figure EV1:**
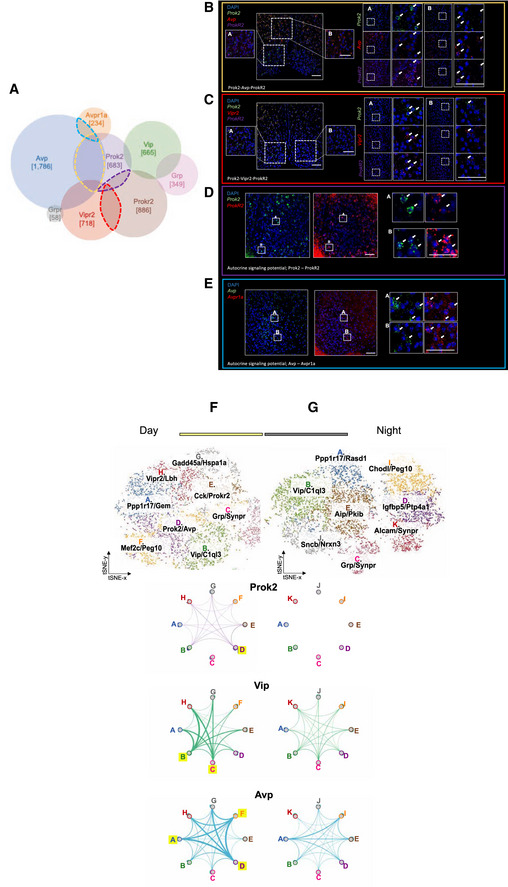
Venn diagram of non‐/overlapping SCN neuronal sub‐populations and their unscaled topology maps, Related to Figs 3 and 4 ASchematic view of populations of interest from Venn analysis of scRNAseq data from daytime SCN neurons.B–ERepresentative RNAScope in situ hybridisation images from SCN cryostat sections to reveal overlap/non‐overlap of neuropeptide/receptor‐expressing cells. (B) Cells expressing *Prok2* (green) and/or *Avp* (red). (C) Cells expressing *Vipr2* and/or *ProkR2*. (D) Potential autocrine signalling in cells expressing *Prok2* ^+^ and/or *ProkR2* ^+^. (E) Cells expressing *Av*p and/or *Avpr1a*. Left: 40×, right 63×, scale bar: 100 µm. For each gene set, panel A and B show magnified 63× images. White arrows highlight the overlap‐/non‐overlap of neuropeptide/ receptor‐expressing cells.F, GInferred topology of neuropeptidergic signalling axes between identified neuronal sub‐populations in SCN from circadian day (F) or circadian night (G). Inter‐cluster signalling is unscaled as connections are weighted by highest expression count measured across the entire dataset. Clusters are numbered according to their size and are annotated in the overview t‐SNE for day (bottom left) and night (top right), respectively. Source data are available online for this figure.

**Figure EV2:**
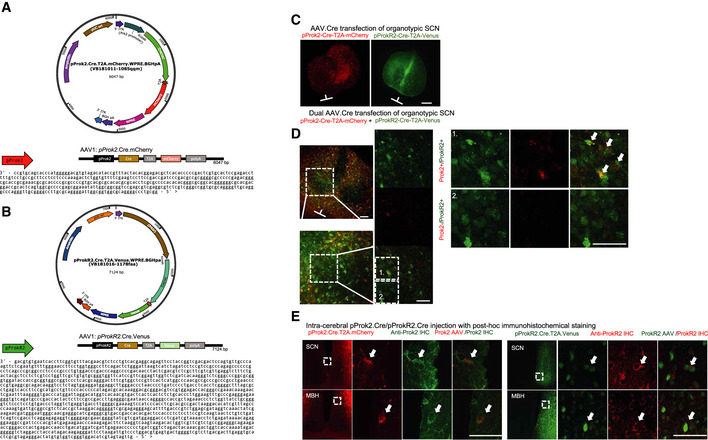
Annotated genomic information and transfection efficacy and efficiency analysis of adeno‐associated viruses used, Related to Fig 5 ATop: *pProk2*.Cre.T2A.mCherry AAV1 map. Bottom: 400bp sequence upstream of the *Prok2* gene driving Cre.T2A.mCherry.BTop: *pProkR2*.Cre.T2A.Venus AAV1 map. Bottom: *ProkR2* containing sequence used to drive Cre.T2A.Venus. *pProk2*.Cre.T2A.mCherry and *pProkR2*.Cre.T2A.Venus.CConfocal images of representative organotypic SCN slices transfected with AAV1:*pProk2*.Cre.T2A.mCherry (left) or AAV1:*pProkR2*.Cre.T2A.Venus (right). Scale bar = 500 µm. Orientation bars depict the horizontal axis of the optic chiasm and the vertical axis of the third ventricle for each SCN. Both images acquired using 10× air objectives with a pinhole aperture set at 1 airy unit (AU).DConfocal images of representative organotypic SCN slices dual‐transfected with both *pProk2*.Cre.T2A.mCherry and *pProkR2*. Cre.T2A.Venus taken at 20, 40 and 63×. Scale bar = 50 µm. Areas annotated as 1 and 2 were selected to show regions with (1) dual‐transfected SCN cells (white arrows), and (2) only *ProkR2* ^+^ SCN cells.EConfocal images of SCN sections from mice injected stereotaxically with the AAV vectors in the region of the SCN (*n* = 6 mice injected with AAV1:*pProk2*.Cre.T2A.mCherry, *n* = 6 mice injected with AAV1:*pProkR2*.Cre.T2A.Venus). The mCherry and Venus reporters are seen in and around the SCN and fluorescent immunostaining for endogenous Prok2 and ProkR2 immuno‐reactivity (‐ir) confirms left: *pProk2*.Cre targeted cells (red) showing cytosolic Prok2*‐ir* (green) and right: ProkR2*‐ir* (red) on the membrane of *pProkR2*.Cre cells (red). White arrows highlight cells co‐expressing mCherry/ Venus reporter and fluorescent immunostaining for Prok2 or ProkR2.

**Figure EV3:**
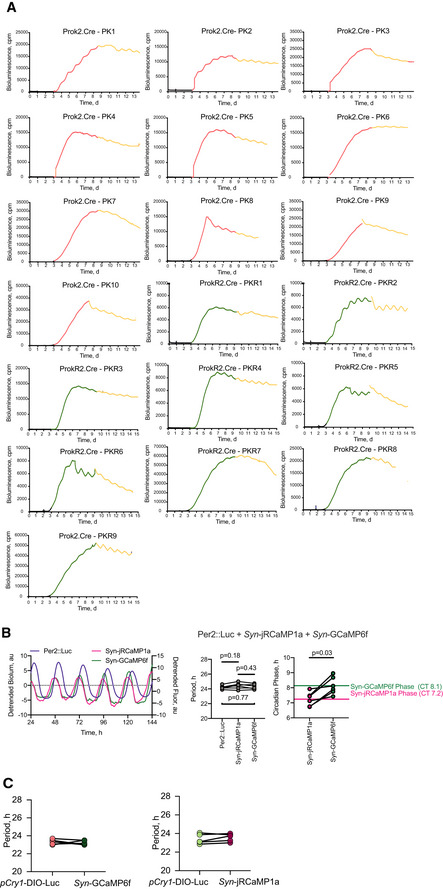
Raw bioluminescent traces mapping circadian functions of SCN *Prok2* ^+^ and *ProkR2* ^+^ cells, Related to Fig 5 ARaw bioluminescent traces of all WT SCN slices transfected with *pCry*.DIO.Luc, *pProk2*.Cre.T2A.mCherry (red, *n* = 10) or *pProkR2*.Cre.T2A.Venus (green, *n* = 9), and after media change (yellow).BRepresentative detrended bioluminescence traces phase‐mapping the intra‐cellular Ca^2+^ reporter AAVs (*Syn*‐GCaMP6f, green, and *Syn*‐jRCaMP1a, red) against whole‐field SCN Per2::Luciferase (purple). No difference in the slice periods reported by SCN Per2::Luciferase bioluminescence or Syn‐jRCaMP/Syn‐GCaMP rhythms were seen (one‐way ANOVA, *P* > 0.05). *Syn*‐GCaMP6f‐associated signal shows a circadian phase at CT8.1 which is significantly delayed to that of *Syn*‐jRCaMP1a at CT7.2 (Wilcoxon matched‐pairs signed rank test, *P* = 0.03).CCircadian period of Cry1‐Luc and GCaMP6f rhythms from *Prok2* ^+^ cells (*P* = 0.64, above) or jRCaMP1a rhythms from *ProkR2* ^+^ cells (paired *t*‐test, *P* = 0.27). Source data are available online for this figure.

**Figure EV4:**
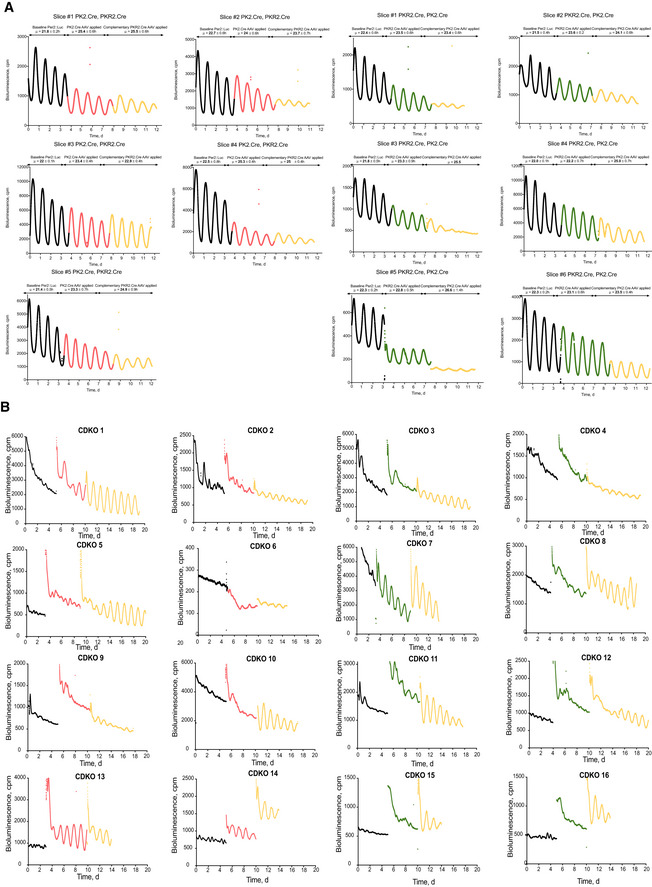
Raw bioluminescent traces of Cry1^−/−^ SCN or Cry1,2^−/−^ SCN complemented with Cry1 in *Prok2* ^+^ and/or *ProkR2* ^+^ cells, Related to Figs 7 and 8 ARaw bioluminescent traces of all Cry1^−/−^ SCN slices transfected with *pCry*.DIO.eGFP, *pProk2*.Cre.T2A.mCherry (red, *n* = 5) or *pProkR2*.Cre.T2A.Venus (green, *n* = 6), followed by the other *pProk2*.Cre or *pProkR2*.Cre‐expressing AAV (yellow). The mean period, µ, measured after each AAV transfection is shown on each respective plot.BRaw bioluminescent traces of all Cry1^−/−^Cry2^−/−^ SCN slices transfected with *pCry*.DIO.eGFP, *pProk2*.Cre.T2A.mCherry (red, *n* = 8) or *pProkR2*.Cre.T2A.Venus (green, *n* = 8), followed by the other *pProk2*.Cre or *pProkR2*.Cre‐expressing AAV (yellow). Source data are available online for this figure.

**Figure EV5:**
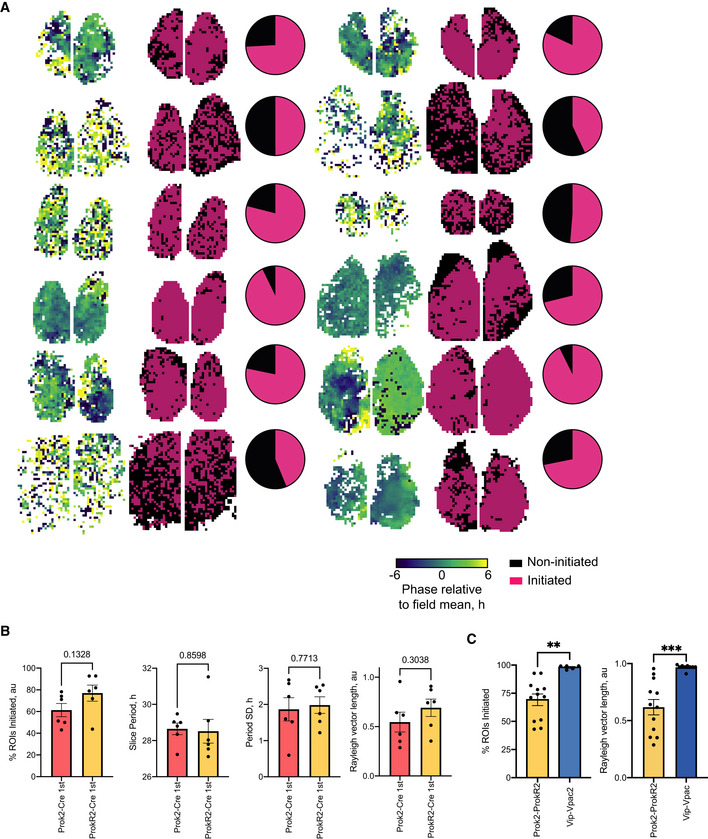
Spatio‐temporal pattern of Per2::Luciferase bioluminescence analysis of Cry1,2^−/−^ SCN initiated by complementation of Cry1 in *Prok2* ^+^/*ProkR2* ^+^ cells, Related to Fig 8 AFirst and fourth column: phase map plots of Per2::Luciferase bioluminescence in *Prok2* ^+^/*ProkR2* ^+^ *Cry1*‐initiated SCN slices showing spatial phase synchronisation across the slices (phases colour‐coded relative to the mean phase of each slice). Second and fifth column: Binary representation of initiated slice elements (CCD recorded bioluminescence pixels) – black indicating non‐initiated and pink initiated elements. Third and sixth column: pie chart of initiated and non‐initiated slice elements.BThe order of initiation of either *Prok2* ^+^ or *ProkR2* ^+^ cells does not affect the % of rhythmic elements within the slice, the period of initiated rhythm, the inter‐slice variability of period or the tightness of coupling within the slice as calculated by the mean Rayleigh vector length (unpaired *t*‐tests, respectively, *n* = 6 for each cohort, mean ± SEM).CLeft: histogram of the % of rhythmic ROIs in Prok2/ProkR2 (*n* = 12) or VIP/VPAC2 (*n* = 5) initiated SCN slices (taken from Patton *et al*, 2020). An unpaired *t*‐test, *P* = 0.003, shows significantly less rhythmic ROIs in the Prok2/ProkR2 (69.2 ± 5%) versus VIP/VPAC2 (98 ± 0.8%) initiated SCN. Right: Rayleigh vector lengths for Prok2‐ProkR2 (*n* = 12) and VIP‐VPAC2 (*n* = 9, Patton *et al*, 2020) initiated SCN slices. Mean ± SEM, **P = 0.003, ***P = 0.0003. Source data are available online for this figure.

To gain insight into the neuropeptidergic topology of the SCN, a Venn diagram summarised the degree of overlap between the ligand‐ and receptor‐expressing cells across these four signalling axes (Fig 3D), using circadian daytime scRNASeq data when the axes were most pronounced. The *Vip* ^+^ and *Vipr2* ^+^ populations were distinct from each other, with limited capacity for autocrine signalling. In contrast, the *Avp* ^+^ and *Avpr1a* ^+^ populations (*Avpr1b* was not expressed at detectable levels) showed some overlap and, thus, autocrine potential. A limited capacity for VIP‐to‐VPAC2/AVP‐to‐AVPR1A serial signalling arose from co‐expression of *Vipr2* and *Avp*. In the *Grp*‐*Grpr* axis, the ligand and receptor populations were clearly segregated. *Grp* ^+^ cells were a sub‐population of *Vip* ^+^ cells, but their efferent (likely paracrine) targets diverged because of limited co‐expression of *Grpr* and *Vipr2*. The organisation of Prok2‐mediated signalling exhibited a limited capacity for autocrine signalling, the *Prok2*‐ and *ProkR2*‐expressing populations being distinct: only 10% of *Prok2* ^+^ cells co‐expressed *ProkR2*, and 7% of *ProkR2* ^+^ cells also expressed *Prok2* (Fig EV1D). Moreover, the *Prok2*‐to‐*ProkR2* axis had only limited overlap with the other three axes: co‐expression with *Grp*/*Grpr* was < 1%, and *Vip* 3%. Of *ProkR2* cells, 18% co‐expressed *Vipr2*, indicative of some convergence in Prok2‐ and VIP‐mediated signalling. The major overlap between axes, however, was principally because of co‐expression of *Avp* in 51% of *Prok2* ^+^ cells (Fig EV1A). These pathways are, nevertheless, divergent as there was little overlap between target cells expressing *ProkR2* and *Avpr1a*. These overlapping sub‐populations were cross‐validated by *in situ* hybridisation (Fig EV1, EV2, EV3, EV4, EV5).

**Figure 4:**
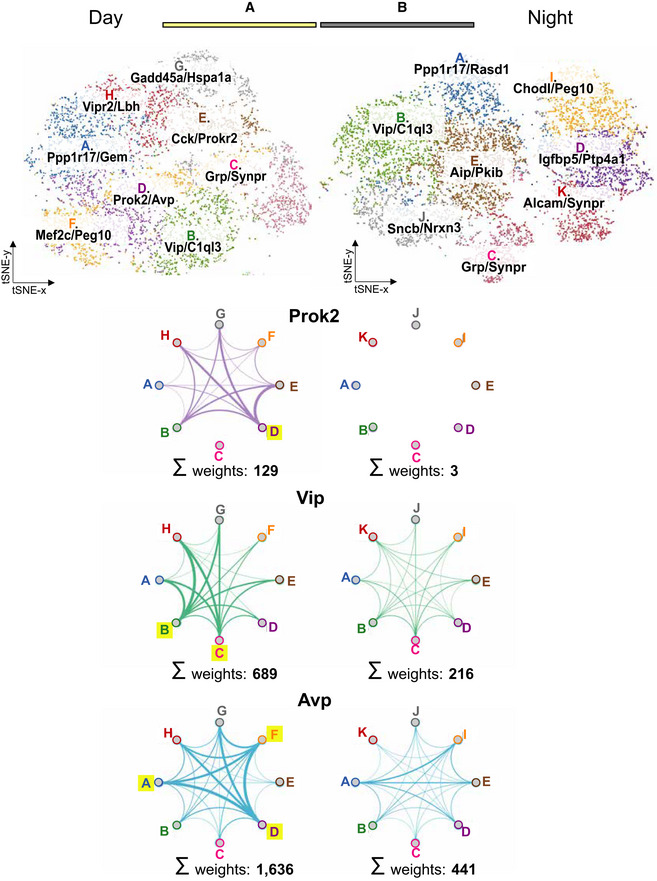
Topology of transcriptionally inferred SCN signalling axes Schematic of inferred topology of neuropeptidergic signalling axes between SCN neuronal sub‐populations in circadian day. Top: clusters are annotated in the overview t‐SNE for circadian day (A) and night (B), respectively. Below: the identified SCN sub‐populations are depicted as nodes of each neuropeptide receptor network, connected by edges that are weighted (shown as differential line thickness) based on the average count of the respective cellular gene expression for each ligand and receptor. The nodes of the inferred topology networks are colour‐coded to match those of the overview t‐SNE plots. Nodes further highlighted in yellow mark ligand‐enriched source clusters. The edges are colour‐coded for each neuropeptide receptor signalling pair: *Prok2*/*ProkR2* – purple, *Vip*/*Vipr2* – green, *Avp*/*Avpr1a* – blue. The sum of edge weights within each day and night inferred signalling network is presented below each map to provide a quantification of the overall degree of connectivity. Source data are available online for this figure.

Having identified SCN neuronal populations and their putative circuit‐based relationships, we then sought to quantify the degree of neuropeptidergic signalling between them by exploiting the transcriptional profiles of SCN cells collected in circadian day or night. The strength of intra‐SCN communication used by each neuropeptide ligand‐receptor pair was inferred from the expression level of each gene of interest in each cluster. More specifically, the average cellular count of the neuropeptide gene expression across one cluster was multiplied with the average cellular count of the corresponding neuropeptide receptor gene expression in another cluster (Zingg *et al*, 2014; Bentley *et al*, 2016). For example, the strength of *Prok2*‐dependent signalling from Group A to Group B was represented as the product of the average cellular count of *Prok2* in Group A and *ProkR2* in Group B, and scaled to its respective overall expression range. The strength and directionality of Prok2, VIP and AVP signalling between SCN sub‐populations at circadian day (Fig 4A, see Fig EV1F and G for the unscaled maps) formalises the Venn diagram (Fig 3D). The overall degree of connectivity within each inferred signalling network is further quantified as the sum of the edge (connection) weights (Fig 4A, sigma values). The distinct topology inferred for the signalling axes VIP‐VPAC2 and AVP‐AVPR1A shows segregated inter‐nodal signalling: VIP and AVP axes have separate source nodes, VIP projects across the SCN network to nodes including those expressing AVP/AVPR1A (Group A), and whilst AVP can signal between its source nodes (Groups A, F and D), VIP does not. The neuropeptidergic network could therefore be viewed topologically as clusters capable of secreting peptides acting as source or “distributor” nodes signalling to their interacting partners capable of receiving and interpreting peptidergic signals as “integrator” nodes (Sporns *et al*, 2004; Rubinov & Sporns, 2010). Importantly, network topology of Prok2, VIP and AVP signalling was also examined at circadian night (Fig 4B) and revealed a marked shift as all neuropeptide source clusters were poorly defined. Most strikingly, Prok2‐ProkR2 signalling was dismantled at night when compared to its prominence during the circadian day. Thus, Prok2 signalling within the SCN exhibited strong potential for regulating network‐wide functions in a time‐dependent manner.

### Circadian properties of SCN Prok2^+^ and ProkR2^+^ cells

ScRNASeq highlighted the *Prok2* ^+^‐*ProkR2* ^+^ cellular axis as a central, circadian‐regulated component of SCN circuit topology. To explore its function, we designed and validated adeno‐associated viral (AAV) vectors to express Cre‐recombinase under the *Prok2* or *ProkR2* regulatory sequences: AAV1:*pProk2. Cre.T2A.mCherry* and AAV1:*pProkR2. Cre.T2A. Venus* (Fig EV2A and B). Intersectional use of the Cre‐lox system thereby allowed us to express reporters of circadian function and manipulate the cell‐autonomous TTFL of *Prok2* ^+^ and *ProkR2* ^+^ cells in a specific, conditional manner. The AAVs readily transfected SCN slices, expressing fluorescence with appropriate cellular distribution and abundance (Fig EV2C and D). Approximately 265 ± 39 *Prok2* ^+^ cells and 317 ± 36 *ProkR2* ^+^ cells were identified in each slice (*n* = 10). These relative proportions (84% Prok2/ProkR2) accord with the daytime scRNASeq data, i.e. 77% (683/886) Prok2. Similarly, in dual‐transfected SCN the AAVs revealed a low degree of co‐expression: only 10% of mCherry‐labelled *Prok2* ^+^ cells emitted *ProkR2* ^+^‐driven Venus fluorescence, whilst 9% of *ProkR2* ^+^ Venus‐labelled cells expressed mCherry‐fluorescence (scRNASeq: 10% and 7%, respectively). Dual *in situ* hybridisation also showed limited overlap: 11 ± 1% of Prok2 cells expressed ProkR2‐signal and 9.6 ± 1% of ProkR2 cells co‐emitted Prok2 signal. Finally, AAV vectors were injected stereotaxically into the SCN region of mice and confocal microscopy revealed expression of the mCherry and Venus reporters in and around the SCN. Immunostaining for endogenous Prok2 and ProkR2 immuno‐reactivity (‐ir) confirmed Cre‐expressing cellular identity: *pProk2*. Cre‐targetted cells showed cytosolic Prok2‐ir, whilst *pProkR2*. Cre cells showed membrane‐localised ProkR2‐ir (Fig EV2E). The newly developed *pProk2*. Cre and *pProkR2*. Cre vectors therefore targeted *Prok2* ^+^ and *ProkR2* ^+^ SCN cells, respectively, to provide efficient and specific genetic access.

**Figure 5:**
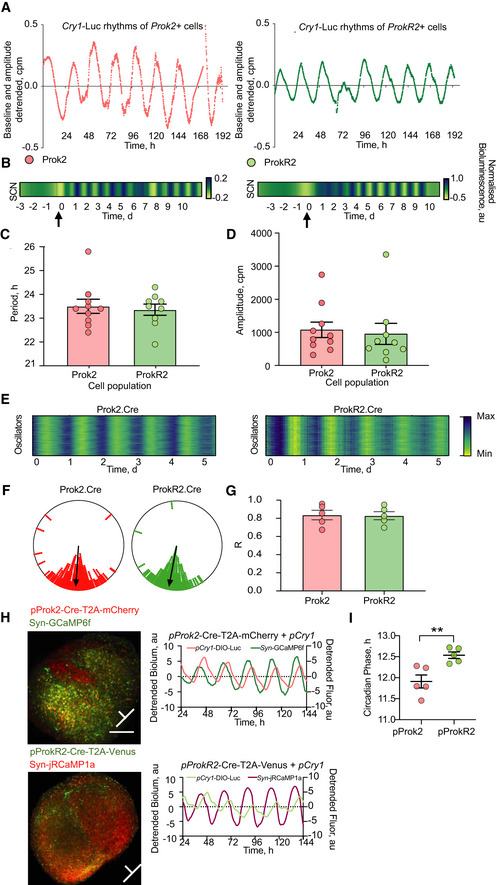
Circadian functions of SCN *Prok2* ^+^ and *ProkR2* ^+^ cells ARepresentative, detrended *Cry1*‐luc traces of emitted bioluminescence (photon count values) from 4 SCN slices, pre‐transfected with AAV‐*pCry1*‐DIO‐Luciferase after addition of (left) *pProk2*.Cre.T2A.mCherry (red, cohort *n* = 10) or (right) *pProkR2*.Cre.T2A.Venus (green, cohort *n* = 9) transfection.BRaster plots (yellow = trough, blue = peak) of *Cry1*‐luc bioluminescence oscillations from a representative slice of each cohort. The black arrow marks the time‐point of *pProk2*.Cre.T2A.mCherry or *pProkR2*.Cre.T2A.Venus AAV transfection, thus resulting in the evolution of rhythmic *Cry1*‐luc bioluminescence.CThe period of *Cry1*‐luc bioluminescence rhythms in SCN slices transfected with either *pProk2*.Cre.T2A.mCherry or *pProkR2*.Cre.T2A.Venus does not differ between treatments (*P* = 0.71, *n* = 10, 9 SCN slices per group, respectively).DSimilarly, the amplitude of *Cry1‐*luc bioluminescence rhythms from *Prok2* ^+^ SCN cells and *ProkR2* ^+^ SCN cells does not differ between treatments (*P* = 0.67).ERepresentative raster plots of single‐cell bioluminescence (yellow = minimum, blue = maximum) from Prok2^Cry1‐luc^ (left) or ProkR2^Cry1‐luc^ (right) cells in single slices.FWithin‐slice cellular synchrony depicted in representative Rayleigh plots of the phases of peak *Cry1*‐luc bioluminescence for Prok2^Cry1‐luc^ (red) and ProkR2^Cry1‐luc^ (green) cells. The Rayleigh plots can be read as the face of a clock with CT0 marking the top and CT12 the bottom of the plot circle. The black arrow depicts the mean peak phase across the individual oscillators.GThe mean Rayleigh vector lengths for Prok2^Cry1‐Luc^ and ProkR2^Cry1‐luc^ (*P* > 0.99) do not differ between the cohorts (*n* = 5 SCN slices per group).HLeft: confocal images of representative SCN slices dual‐transfected with *pProk2*.Cre.T2A.mCherry and *Syn*.GCaMP6f (top), or *pProkR2*.Cre.T2A.Venus and *Syn*.jRCaMP1a (below) (scale bar = 500 µm). Right, above: representative detrended plots of signal from GCaMP6f (green) traces and *pCry1*‐DIO‐Luc, emitted from *Prok2* ^+^ SCN cells (red). Right, below: representative detrended plots of jRCaMP1a (purple) and *pCry1*‐DIO‐Luc emitted from *ProkR2* ^+^ cells (light green). Orientation bars depict the horizontal axis of the optic chiasm and the vertical axis of the third ventricle for each SCN.IAfter registering the respective peaks of [Ca^2+^]_i_ fluorescence to the peaks of Cry1‐luc bioluminescence in each respective cohort, phase‐alignment between the peaks of Prok2^Cry1‐luc^ and ProkR2^Cry1‐luc^ bioluminescence shows that the intra‐cellular clocks of *Prok2* ^+^ cells are phase advanced by ˜0.6 h to those of *ProkR2* ^+^ cells (**P < 0.01) (*n* = 5 SCN slices per group). Data information: All values depicted as mean ± SEM. C, D, G, I unpaired two‐tailed *t*‐tests. Source data are available online for this figure.

We then used the AAVs to monitor TTFL function in *Prok2* ^+^ and *ProkR2* ^+^ SCN cells. Wild‐type organotypic SCN slices were transfected with a Cre‐conditional *Cry1* transcriptional reporter (*pCry.DIO. Luc*) (Patton *et al*, 2020) and subsequently with *pProk2*. Cre.T2A.mCherry or *pProkR2*. Cre.T2A. Venus for its cell‐specific activation. In both cases, as recombination released *pCry1*‐driven luciferase, baseline bioluminescent signal rose (Fig EV3A) and within a few days a clear circadian rhythm of bioluminescence developed (Fig 5A and B). The TTFL ensemble periods and amplitudes of *Prok2* ^+^ and *ProkR2* ^+^ SCN cells were equivalent (Fig 5C and D). Thus, both *Prok2* ^+^ and *ProkR2* ^+^ SCN cells exhibit clear TTFL circadian activity. We then used CCD recording to monitor the TTFL activity of individual Prok2^Cry1‐luc^ cells and ProkR2^Cry1‐luc^ cells across the SCN and revealed tight coupling of cellular rhythms within each sub‐population (Fig 5E), whose significant Rayleigh distributions did not differ between each other (Fig 5F and G).

To phase‐map the TTFL rhythms in *Prok2* ^+^ and *ProkR2* ^+^ cells, we combined bioluminescent recording with fluorescent recording of intra‐cellular calcium levels ([Ca^2+^]_i_), such that the circadian peak of [Ca^2+^]_i_ could be used as a phase reference (Brancaccio *et al*, 2019). This was reported by AAV‐expressed synapsin‐GCaMP6f in *pProk2*. Cre.T2A.mCherry slices and synapsin‐jRCaMP1a in SCN expressing *pProkR2*. Cre.T2A. Venus. Prior recordings against whole‐field Per2::Luciferase phase‐mapped the peaks of GCaMP and jRCaMP as CT8.1 and CT7.2, respectively (Fig EV3B), allowing the *Cry1*‐luc and [Ca^2+^]_i_ oscillations to be aligned in each SCN (Figs 5H and EV3C). The periods of *Prok2* ^+^ and *ProkR2* ^+^ cells were comparable with their respective slice periods, as reported by G/jRCaMP traces. Phase registration with the peak of the respective GCaMP/RCaMP traces revealed a difference, however, in the peak time of *Cry1*‐luc rhythms. Prok2^Cry1‐luc^ cells peaked at CT11.9 ± 0.2 h, whereas ProkR2^Cry1‐luc^ cells peaked later at CT12.5 ± 0.1 h. The difference of ˜0.6 h was significant (*P* = 0.007) (Fig 5I) and parallels previous phase‐mapping of the VIP‐VPAC2 axis in which the TTFL of ligand‐secreting cells was phase advanced relative to the TTFL of their receptor‐expressing targets (Patton *et al*, 2020).

### Control of SCN circadian function by Prok2 and the *Prok2* ^+^‐*ProkR2* ^+^ cellular axis

**Figure 6:**
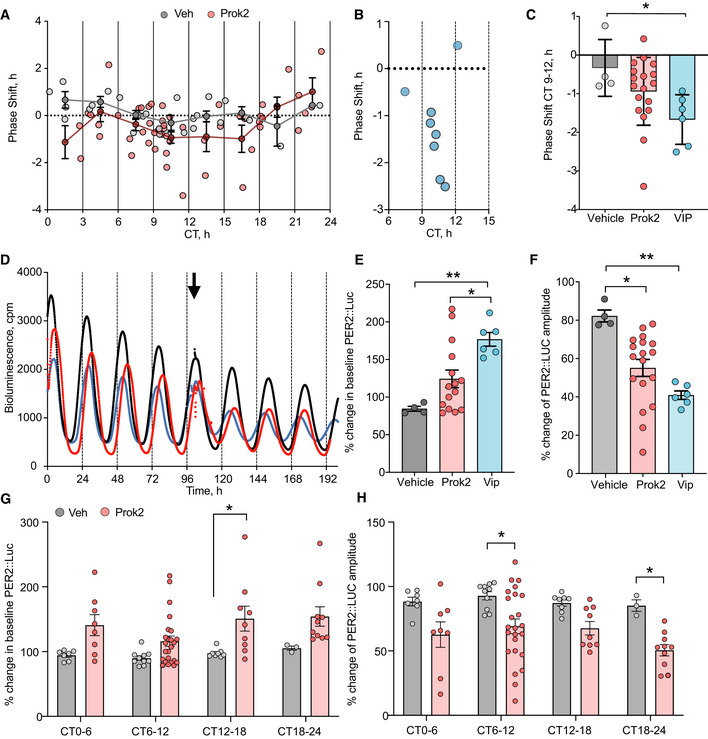
Exogenous Prok2 alters oscillation waveform without altering ensemble phase APhase response curve for Prok2‐treated (red) and vehicle‐treated (grey) SCN slices (*P* > 0.05). The phase shift (h), calculated from the mean of the first three peaks after Prok2 application in Per2::Luciferase rhythms, is plotted against the CT of treatment. Faded circles (grey = vehicle, red = Prok2) show individual SCN data points, whilst the mean values across the vehicle‐treated or Prok2‐treated cohort within 3‐h time‐bins are shown bold.BApplication of VIP (blue) delivered during CT7‐13 served as a positive control and elicited robust phase delays of ongoing Per2::Luciferase SCN slice oscillations when administered between CT9 and CT12.CComparison of phase shifts measured across each treatment (vehicle versus VIP, *P = 0.04) when administered between CT9 and CT12.DRepresentative Per2::Luciferase traces before and after administration (arrow) of vehicle (black), Prok2 (red) or VIP (blue) within CT9‐12.EComparison across treatment cohorts of the relative change in baseline of Per2‐driven bioluminescence when vehicle, Prok2 or VIP was applied within CT9‐12. Only VIP is statistically shown to significantly raise Per2::Luciferase baseline levels (*P* = 0.0007; vehicle versus Prok2, ns. *P* = 0.17; vehicle versus VIP, **P = 0.002, Prok2 versus VIP, *P = 0.02).FThe relative change in amplitude of Per2::Luciferase rhythm after treatment with Prok2 or VIP significantly decreases compared to vehicle application (*P* = 0.002; vehicle versus Prok2, *P = 0.01, vehicle versus VIP, **P = 0.001).GRelative change in baseline Per2::Luciferase measured from the first cycle after treatment across circadian time, binned in 6 h intervals (treatment factor *P* < 0.0001). Prok2 within CT12‐18 significantly increased baseline Per2::Luciferase more than vehicle, *P = 0.01.HRelative change in the amplitude of the first Per2::Luciferase cycle measured after treatment across circadian time, binned in 6‐h intervals (treatment factor *P* < 0.0001). Prok2 within CT6‐12 and CT18‐24 significantly decreased Per2::Luciferase rhythm amplitude compared to vehicle, CT6‐12 *P = 0.01, CT18‐24 *P = 0.04. Data information: All values depicted as mean ± SEM. C, E, F one‐way ANOVA with Tukey’s multiple comparisons; A, G, H, I two‐way ANOVA with Sidak’s multiple comparisons. For G, H, CT0‐6 *n* = 7 vehicle and *n* = 8 Prok2 treated, CT6‐12 *n* = 10 vehicle and *n* = 24 Prok2 treated, CT12‐18 *n* = 8 vehicle and *n* = 9 Prok2 treated, CT18‐24 *n* = 3 vehicle and *n* = 10 Prok2 treated. Source data are available online for this figure.

**Figure 7:**
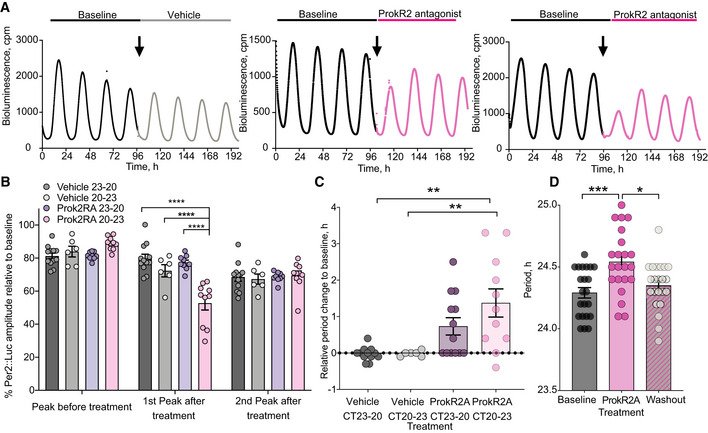
SCN rhythm amplitude and period are determined by endogenous Prok2 signalling ARepresentative Per2::Luciferase traces before (black) and after vehicle (grey) or Prok2RA (pink) topical application of SCN slices.BComparison of the Per2::Luciferase rhythm amplitude of the cycle preceding and the first and second cycles succeeding vehicle (applied CT23‐20 dark grey *n* = 12 SCN slices, CT‐20‐23 light grey *n* = 6 SCN slices) or Prok2RA (applied CT23‐20 purple *n* = 13 SCN slices, CT20‐23 pink *n* = 11 SCN slices) treatment (interaction, *P* < 0.0001). A significant decline in amplitude occurred when Prok2RA signalling was blocked between CT20 and CT23 (****P < 0.0001).CChange in SCN period after treatment with vehicle (grey) or Prok2RA (pink) between CT23‐20 and CT20‐23 (vehicle CT23‐20 versus Prok2RA CT20‐23, **P = 0.009; vehicle CT20‐23 versus Prok2RA CT20‐23, **P = 0.001) (*n* = 6, 12, 13 and 11 independent SCN slices, respectively).DProk2RA application resulted in a significant period lengthening effect of ongoing SCN ensemble Per2::Luciferase rhythms, which was reversed upon drug washout (*P* = 0.0002, ***P = 0.0009, *P = 0.01) (*n* = 22 independent SCN slices with repeated measure). Data information: All values depicted as mean ± SEM. B two‐way ANOVA with Sidak’s multiple comparisons; C,D one‐way ANOVA with Tukey’s multiple comparisons. Source data are available online for this figure.

Given that both *Prok2* ^+^ and *ProkR2* ^+^ cells express clear TTFL rhythms, with *Prok2* ^+^ cells phase‐leading, and *Prok2* being a clock‐controlled gene, we asked whether Prok2 relays circadian information to *ProkR2* ^+^ cells. We first tested the effect of exogenous Prok2 on the SCN network‐level oscillation, generating a phase‐response curve for Per2::Luciferase bioluminescence rhythms in slices treated with 10 µM Prok2 (*n* = 51) or vehicle (*n* = 28) (Appendix Fig S3A and B). Treatment with 10 µM VIP (*n* = 6) between CT9‐12 provided a positive control (Hamnett *et al*, 2019). Exogenous Prok2 had no systematic phase‐shifting effect across circadian time (Fig 6A). In comparison, at CT9‐12 VIP reliably phase‐delayed the ongoing oscillation (Fig 6B and C). We then questioned whether Prok2 application had other actions on the Per2‐reported TTFL. VIP increased the baseline of Per2::Luciferase at CT9‐12 (Fig 6D and E) (Hamnett *et al*, 2019), whereas Prok2 did not (Fig 6E) (Appendix Fig S3C). Nevertheless, administration of VIP or Prok2 between CT9 and CT12 reduced the amplitude of Per2‐driven bioluminescence (Fig 6D and F). We then tested other phases and found that Prok2 treatment between CT12 and CT18 significantly increased the Per2::Luciferase baseline (Fig 6G) and Prok2 decreased the amplitude of Per2::Luciferase between CT18‐24 (in addition to CT6‐12) (Fig 6H). Thus, although not capable of re‐phasing the ensemble oscillation, exogenous Prok2 had an overall effect on the waveform of the Per2::Luciferase rhythm, increasing baseline and suppressing amplitude. We then asked whether blocking endogenous Prok2 signalling would perturb ongoing Per2::Luciferase oscillations (Fig 7A). Treatment of SCN slices with a Prok1R/Prok2R antagonist (ProkR2A, 10 µM) at various time‐points across the day revealed a particularly sensitive phase between CT20 and CT23 in which the subsequent peak of Per2‐driven bioluminescence was significantly suppressed (Fig 7B) (Appendix Fig S4A–C). Treatment at other phases did not affect Per2 levels. In addition, blocking ProkR2 between CT20 and CT23 significantly lengthened the period of the SCN ensemble rhythm (0.3 ± 0.06 h) compared to vehicle application (0 ± 0.1 h) (Fig 7C), an effect reversed by washout of ProkR2A (Fig 7D). Impeding Prok2‐mediated activation of *ProkR2* ^+^ SCN cells therefore had phase‐dependent, network‐level effects on SCN circadian function: acute Per2 suppression and a lengthening of the ensemble period, highlighting an ongoing circadian role for endogenous Prok2 in the SCN.

**Figure 8:**
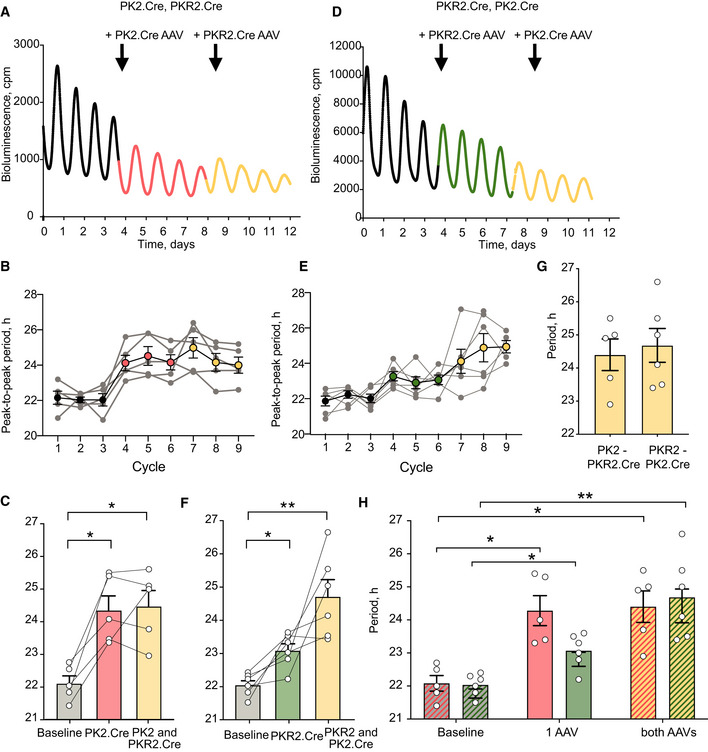
*Prok2* ^+^ and *Prok2R* ^+^ cells control SCN circadian period ARepresentative Per2::Luciferase trace of Cry1^‐/‐^ SCN slice at baseline (black) and after AAV‐mediated (arrows) Cry1 complementation in *Prok2* ^+^ cells (red) or *ProkR2* ^+^ cells (yellow).BCorresponding peak‐to‐peak period plots for baseline (black), *Cry1*‐expressed in *Prok2* ^+^ (red), and then in *ProkR2* ^+^ cells (yellow). Individual slices are depicted as grey traces, and filled points are their mean ± SEM.CGroup data (*n* = 5) across three Per2::Luciferase cycles as shown in B (*P* = 0.006; baseline versus *Prok2*.Cre *P = 0.04, baseline versus *Prok2*.Cre and *ProkR2*.Cre *P = 0.02, *Prok2*.Cre versus *Prok2*.Cre and *ProkR2*.Cre ns *P* = 0.95).DAs in A but with order of transfection reversed; green = Cry1 only in *ProkR2* ^+^ cells (baseline versus *ProkR2*.Cre *P = 0.04).E, FAs in B and C but with transfection order reversed (green = Cry1 expression in *ProkR2* ^+^ cells only, *n* = 6) (*P* = 0.006; baseline versus *ProkR2*.Cre *P = 0.03, baseline versus *ProkR2*.Cre and *Prok2*.Cre **P = 0.009, *ProkR2*.Cre versus *ProkR2*.Cre and *Prok2*.Cre ns *P* = 0.1).GThe final circadian periods of Cry1^‐/‐^ SCN slice double‐transduced in complementary reverse orders did not differ between each other, *P* = 0.7.HPeriod lengthening occurred at the same rate when *Prok2*.Cre or *ProkR2*.Cre was the first AAV to be applied (*P* = 0.1; *Prok2*.Cre – baseline versus 1 AAV, *P = 0.01; baseline versus both AAVs, *P = 0.04; *ProkR2*.Cre – baseline versus 1 AAV, *P = 0.03; baseline versus both AAVs, **P = 0.01). Data information: All values depicted as mean ± SEM. C, D, E, F one‐way ANOVA with Tukey’s multiple comparisons; G unpaired two‐tailed *t*‐test; H two‐way ANOVA with Sidak’s multiple comparisons. In histograms individual points are individual slices, bars are their mean ± SEM. Source data are available online for this figure.

We then focussed on the cellular elements of the signalling axis and asked whether *Prok2* ^+^ and/or *ProkR2* ^+^ SCN cells can direct other emergent properties. We first used genetic complementation of short period (˜22 h) Cry1‐null SCN by AAV‐mediated conditional expression of Cre‐dependent Cry1 (*pCry1*‐DIO‐Cry1::EGFP). If *Prok2* ^+^ and/or *ProkR2* ^+^ SCN cells are period‐setting nodes of the SCN network, Cry1 complementation targeted to them should lengthen both their cell‐autonomous period and also the period of the entire SCN network. Cry1 complementation in *Prok2* ^+^ cells alone significantly lengthened Per2‐reported TTFL period from 22.1 ± 0.2 h to 24.3 ± 0.5 h (Fig 8A–C) (Fig EV4A). Additional expression of Cry1 in *ProkR2* ^+^ cells had no further effect (period = 24.4 ± 0.5 h). Cry1 expression in *ProkR2* ^+^ cells, alone, was equally effective, slowing ensemble period from 22 ± 0.1 h to 23.1 ± 0.2 h (Figs 8D–F and EV4A). Again, additional expression in *Prok2* ^+^ cells had no significant effect (24.7 ± 0.5 h). The ultimate periods in the two treatment cohorts of double‐complemented SCN were not significantly different (Fig 8G). Similarly, the rate of effect did not differ with the order of population‐specific Cry1 expression (Fig 8H). Importantly, the sinusoidal profile of Per2‐driven bioluminescence was not distorted as period lengthened, indicating that all cells in the slice adopted the longer period and maintained a stable phase relationship.

**Figure 9:**
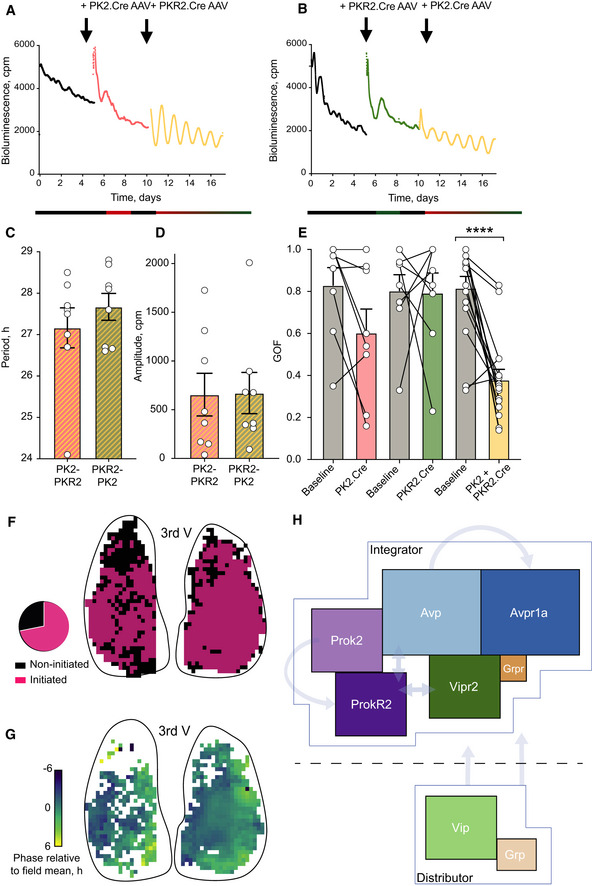
*Prok2* ^+^ and *Prok2R* ^+^ cells can initiate de novo SCN network rhythms ARepresentative Per2::Luciferase traces of Cry1,2^−/−^ SCN slices at baseline (black, arrhythmic), after Cry1 complementation in *Prok2* ^+^ cells (red) and further complementation in *ProkR2* ^+^ cells (yellow, rhythmic) (cohort *n* = 8).BAs in A but order of complementation reversed (green = Cry1 complementation in *ProkR2* ^+^ cells) (cohort *n* = 8).CSCN period after Cry1 expression in both *Prok2* ^+^ and *ProkR2* ^+^ cells did not differ with order of transduction (*P* = 0.4).DBioluminescence rhythm amplitude did not differ with order of complementation (*P* = 0.96).EPaired goodness‐of‐fit values for Per2::Luciferase oscillations of baseline, single‐transfection and double‐transfection SCN (*P* = 0.02, *Prok2*.Cre before and after, ns. *P* = 0.17; *ProkR2*.Cre before and after, ns. *P* = 0.99; both *Prok2*.Cre and *ProkR2*.Cre applied before and after ****P < 0.0001).FBinary representation of initiated slice elements of a representative SCN slice (CCD recorded bioluminescence pixels) – black indicates non‐initiated and pink indicates initiated, i.e. rhythmic elements (cohort *n* = 12).GPhase map of Per2::Luciferase bioluminescence in a representative *Prok2* ^+^/*ProkR2* ^+^ *Cry1*‐initiated SCN slice showing spatial phase synchronisation across the slices (phases colour‐coded relative to the mean phase of each slice).HInferred neuronal group interactions in relation to circuit‐level contribution as “Distributor” or “Integrator” hubs. Data information: All values depicted as mean ± SEM. C, D unpaired two‐tailed *t*‐tests; E two‐way ANOVA with Sidak’s multiple comparisons. Source data are available online for this figure.

Thus, both *Prok2* ^+^ and *ProkR2* ^+^ SCN cells are able to act as period‐setting nodes within the SCN circuit, imposing their cell‐autonomous period on the network. But to what extent are they able, individually or in combination, to drive the SCN oscillator when they alone contain a functional cell‐autonomous TTFL? To address this, we conditionally expressed Cry1 in either *Prok2* ^+^ or *ProkR2* ^+^ cells in arrhythmic SCN lacking both Cry1 and Cry2 (Patton *et al*, 2020). Cre‐conditional expression of *Cry1* to activate the TTFL in either *Prok2* ^+^ or *ProkR2* ^+^ cells alone was insufficient for reliable initiation of network‐level SCN rhythms (Figs 9A and B, and EV4B). When the cell‐autonomous TTFL was complemented in both *Prok2* ^+^ and *ProkR2* ^+^ cells by additional transduction, however, robust *de novo* SCN rhythms were initiated. Importantly, the period of these rhythms was ≥ 27 h, characteristic of a Cry1‐driven, Cry2‐null mouse or SCN (van der Horst *et al*, 1999; Anand *et al*, 2013) and comparable to that observed in pan‐neuronally driven expression of *Cry1* (Brancaccio *et al*, 2019). Neither the ultimate period nor the ensemble amplitude differed depending on the order of sub‐population restoration (Fig 9C and D). Goodness‐of‐fit values measured from Per2::Luciferase traces were used to quantify the quality of the rhythms before viral transfection, and after each of the three possible (single and dual) transfection conditions. There was no significant improvement of rhythm quality when only one population was targeted, but a significant oscillation occurred when both populations had been targeted (Fig 9E). These results show that, together, *Prok2* ^+^ and *ProkR2* ^+^ SCN cells are able to initiate network rhythms if they are the only cell‐autonomous oscillators within the SCN.

We then asked whether the spatio‐temporal pattern of Per2::Luciferase bioluminescence initiated by complementation of *Prok2* ^+^/*ProkR2* ^+^ cells, and which reflects intercellular coupling, mirrored that seen in WT SCN or in *Vip* ^+^/*Vipr2* ^+^ complemented Cry‐null slices (Patton *et al*, 2020). CCD recordings showed that the order in which the Prok2/ProkR2 axis was activated did not affect the pattern of circuit‐level oscillation; neither the ensemble slice period nor the degree of synchronicity (as determined by the coupling of bioluminescence phases observed) differed between dual‐transduced cohorts (Fig EV5A and B). We then constructed SCN‐wide maps of the local strength of oscillation and its phase. This showed that the cell‐specific expression of Cry1 in *Prok2* ^+^/*ProkR2* ^+^ cells did not initiate rhythms in the entire SCN network. Rather, local rhythmicity was evident in 69 ± 5% of the SCN (Fig 9F). Furthermore, in contrast to the regionally specific phase‐clusters of Per2::Luciferase activation observed in WT SCN and in Cry‐null SCN complemented in *Vip* ^+^ and *Vipr2* ^+^ cells, CCD recordings revealed a more temporally homogeneous phase‐locking of bioluminescence following joint complementation of *Prok2* ^+^and *ProkR2* ^+^ cells (Fig 9G). Thus, whereas the VIP/VPAC2 signalling axis engages the entire (98 ± 0.8%) SCN network (Patton *et al*, 2020), acting as a distributor node, the Prok2/ProkR2 axis engages only ˜70% (Fig EV5C) and it also fails to establish the phase‐distribution characteristic of WT and *Vip* ^+^/*Vipr2* ^+^‐complemented SCN. This reveals the Prok2/ProkR2 signalling axis as an integrator node and highlights the distinct roles and mechanisms for the VIP/VPAC2 and Prok2/ProkR2 signalling axes in directing ensemble SCN rhythms (Fig EV5C).

## Discussion

SCN cellular coupling determines the properties of its network‐level oscillation and the circadian cues that are broadcast to control organismal behaviour and physiology. We used scRNASeq to identify the individual cellular components of the circuit, define its network topology and show how that topology is modified across circadian time. We thereby revealed that *Prok2* ^+^ and *ProkR2* ^+^ cells are circadian‐regulated nodes, able to determine SCN ensemble period and initiate circadian oscillations in an otherwise circadian‐incompetent network. Complementing these findings, endogenous Prok2/ProkR2 signalling tunes the period and amplitude of circuit oscillations. These results extend understanding of SCN network topology and the contribution of defined SCN neuropeptidergic populations to the powerful time‐keeping properties of the SCN.

In contrast to the cerebral cortex or cerebellum, which present repeating modules of cellular identity and organisation, the hypothalamus is a neurochemically and structurally diverse collection of distinct nuclei. Transcriptomic analysis of single cells therefore offers a powerful way to explore its organisation (Moffitt *et al*, 2018; Mickelsen *et al*, 2019). Although the SCN is nested within the hypothalamus, meta‐analysis of whole‐tissue transcriptomes has identified its distinctiveness (Brown *et al*, 2017), with the SCN featuring less synaptic and more dense‐core vesicle signalling, consistent with the importance of neuropeptidergic nodes. Indeed, single‐cell transcriptomic analysis of 87 candidate genes revealed the substantial heterogeneity of transcriptionally assigned source or target clusters for paracrine and autocrine signalling (Park *et al*, 2016). More extensive unbiased scRNASeq applied to SCN cells obtained from tissue sections analysed 62,083 cells across varying CT points, although only 3,718 cells were deemed putative SCN cells (Wen *et al*, 2020). Nevertheless, five SCN subtypes were identified with cluster‐specific marker genes, including *Avp*, *Grp*, *Vip*, *Cck* and *C1ql3* and SCN‐enriched genes *Prok2*, *Rasl11b*, *Nr1d1*, *Rgs16* and *Rasd1* exhibited strong circadian expression in serial tissue samples. Our dataset of 17,363 sequenced SCN neurons significantly expands these initial findings, providing a deep transcriptional characterisation of eleven SCN neuronal cell groups, and identifying 817 (274 up‐regulated at day, 543 at night) circadian‐regulated genes in the autonomously oscillating organotypic slice.

The principal aim of our transcriptomic analysis was to reveal topological features of the SCN network. In the conventional ventral “core” and dorsal “shell” model, signalling by VIP is considered strongly directional, from core to shell (Maywood *et al*, 2006; Colwell, 2011; Patton *et al*, 2020) whereas autocrine AVP signalling is distributed across the shell (Yamaguchi *et al*, 2013; Mieda *et al*, 2015, 2016; Ono *et al*, 2016). The identification of eleven distinct SCN neuronal populations by scRNASeq allowed us to expand this binary model by adapting approaches used successfully in *C*. *elegans* and mouse neocortex (Zingg *et al*, 2014; Bentley *et al*, 2016) to infer relationships between cellular nodes, as conferred by neuropeptide ligand and receptor expression. Four inter‐nested neuropeptidergic signalling axes were identified, with complex interactions beyond the linear core‐shell model. There were clear examples of divergence, with VIP/GRP cells addressing separate *Vipr2* ^+^ and *Grpr* ^+^ targets, and convergence via cellular co‐expression of *ProkR2* and *Vipr2*. Based on these findings and *in situ* hybridisation analysis [this study and (Wen *et al*, 2020)], we have identified *Vip* ^+^ cells as a paracrine source cluster whose topological arrangement represents a distributing node, whereas the *Avp* ^+^ and *Prok2* ^+^ clusters can be seen to act as integrating nodes of the SCN network (Fig 9H). Furthermore, comparison of cells from subjective day and night‐time showed how this multiplexed network is dramatically reconfigured across the circadian cycle. It is transcriptionally disassembled in circadian night, co‐incident with neuronal inactivation, and restored for the neurally active day phase. This temporal control of network topology reveals an additional level of sophistication to the SCN pacemaker, and indirectly highlights the importance of nocturnal, astrocytically mediated signalling in stabilising and progressing time‐keeping by the SCN network at night (Brancaccio *et al*, 2017, 2019).

The most pronounced circadian change of network configuration was seen for the Prok2/ProkR2 axis. *Prok2* has four E‐box elements that drive its circadian transcription (Cheng *et al*, 2002) (Cheng *et al*, 2005) (Masumoto *et al*, 2006) and recombinant Prok2 can induce locomotor activity in rats (Cheng *et al*, 2002), whilst genomic ablation of Prok2 in mice attenuates clock‐controlled behaviour and physiology, including glucocorticoid signalling and body temperature rhythms, (Li *et al*, 2006) and mis‐expression of Prok2 disrupts behavioural rhythms (Li *et al*, 2018). Moreover ProkR2^‐/‐^ mice lose precise timing of nocturnal locomotor activity onset (Prosser *et al*, 2007; Jethwa *et al*, 2008) and Prok2^‐/‐^ mice show altered circadian and homeostatic regulation of sleep and disrupted post‐deprivation recovery sleep (Hu *et al*, 2007). Given that SCN TTFL rhythms of ProkR2^‐/‐^ mice appeared normal, these findings placed Prok2 signalling downstream of the SCN clock, as a key output, and argued against any intrinsic network role. Our identification of the Prok2/ProkR2 axis as a pacemaker within the SCN now raises the possibility that at least some of these consequences of global mutation arise from loss of SCN precision. Indeed, Prok2 can suppress GABAergic function in SCN slices in circadian daytime by reducing the amplitude of mini‐inhibitory post‐synaptic currents, disinhibiting the firing rate of SCN cells (Ren *et al*, 2011). This highlights the potential for circuit‐level Prok2‐ProkR2 signalling within the SCN, which we confirmed by showing that exogenous Prok2 increased the baseline level of Per2‐driven bioluminescence, likely reflective of decreased GABAergic tone and thus disinhibited neuronal firing. In contrast, blocking ProkR2 damped the subsequent peak of Per2 expression in circadian day, an effect consistent with increased GABAergic tone, which would attenuate the circadian up‐swing in firing rate and thus activation of cAMP/Ca^2+^ responsive elements, damping Per2 expression (Colwell, 2011; Brancaccio *et al*, 2013). The absence of effect at other phases is possibly because firing rate and CRE activation are spontaneously declining and GABA tone is already high. The strict temporal control of *Prok2* expression would further support phase‐dependence of this regulation. ProkR2 blockade also caused a modest lengthening of SCN period, an effect seen in Prok2‐null mice (Li *et al*, 2006), which may again reflect sustained enhancement of GABAergic tone and consequent attenuation of activity‐induced Per2 (Colwell, 2011). Conditional knockout of Prok2/ProkR2 in the SCN would therefore be expected to enhance GABAergic tone, possibly reducing neuronal firing rate and attenuating Prok2‐dependent and Prok2‐independent output signals, weakening circadian co‐ordination and making the circadian system more prone to environmental disruption.

Given that Prok2R is expressed widely in areas innervated by SCN *Prok2* ^+^ cells (e.g. PVN, medial thalamus and DMN), the newly developed AAVs offer the means for more refined analyses to parse out local and distal effects of Prok2 signalling in both the SCN and its targets. For example, can conditional activation or silencing of these pathways initiate, suppress or otherwise re‐programme circadian behaviour? Importantly, our scRNASeq data show that the Prok2/ProkR2 axis is distinct from the retinorecipient VIP/GRP cells (only 6% of *Vip* and 3% of *Grp* ^+^ SCN neurons co‐express *Prok2*) and this may explain why Prok2 signalling is not involved in phase‐entrainment of the SCN. Its transcriptional regulation by retinal input (Cheng *et al*, 2005) is likely mediated indirectly via VIP‐mediated signalling (Hamnett *et al,* 2019). This provides a clear distinction between the Prok2/ProkR2 and VIP/VPAC2 axes (Hamnett *et al*, 2019; Patton *et al*, 2020).

This distinction was further emphasised by our analysis of pacemaking. Using AAV‐mediated intersection, we demonstrated the role of the distinct *Prok2* ^+^ and *ProkR2* ^+^ cell populations as pacemaking nodes. The unitary profile of Per2::Luciferase bioluminescence confirmed that all Cry1‐deficient SCN cells had adopted the new period and phase, even though the majority did not express Cry1, highlighting the intrinsic pace‐setting role of both populations. The independent actions and absence of additivity with dual transfection are consistent with *ProkR2* ^+^ cells being the ultimate effectors, but subject to afferent regulation by *Prok2* ^+^ cells as indirect effectors. This is in contrast to *Vip* ^+^ and *Vipr2* ^+^ cells, which individually had only a minor or no effect at all, respectively, on SCN period, and only when the cell‐autonomous clocks of *Vip* ^+^ and *Vipr2* ^+^ cells were slowed together did the SCN ensemble period also slow (Patton *et al*, 2020). Thus, the Prok2/ProkR2 and VIP/VPAC2 cellular axes are transcriptionally distinct (only 3.5% of all SCN neurons co‐express *Vip* or *Vipr2* with either *Prok2* or *ProkR2*) and functionally separable pace‐setting nodes within the SCN network: even though they are of comparable abundance (Prok2/ProkR2 represent 19%, VIP/VPAC2 17% of daytime SCN neurons), the Prok2 axis appears the more potent in controlling ensemble period.

As a final test of the potential pacemaking role of *Prok2* ^+^ and *ProkR2* ^+^ cells, we expressed Cry1 in them, individually and together, in arrhythmic Cry1/2‐null SCN. Pan‐neuronal expression of Cry1 in Cry‐null SCN can initiate circadian oscillations with a characteristically long period (Edwards *et al*, 2016; Maywood *et al*, 2018; Brancaccio *et al*, 2019), and this was mirrored in our dataset. The *Prok2* ^+^ and *ProkR2* ^+^ cells, as a unit, exhibited pacemaking properties that matched those of the combined *Vip* ^+^ and *Vipr2* ^+^ cellular unit (Patton *et al*, 2020). CCD imaging revealed an interesting difference, however, because oscillatory behaviour was apparent in only ˜70% of the SCN with circadian‐competent *Prok2* ^+^/*ProkR2* ^+^ cells. This is significantly above their relative abundance (19% *Prok2* ^+^/*ProkR2* ^+^) but appreciably less than the widespread initiation of rhythms (98%) following the combined activation of *Vip* ^+^ and *Vipr2* ^+^ cells. A second contrast was in the circuit‐wide phase relationships. Activated *Vip* ^+^/*Vipr2* ^+^ cells established a network rhythm in which SCN sub‐regions showed temporally defined, phase‐dispersed activation (Patton *et al*, 2020). In contrast, when TTFL rhythmicity was established in *Prok2* ^+^/*ProkR2* ^+^ cells, the SCN‐wide oscillatory cells were tightly phase‐clustered. This emphasises a functional difference between the neurochemical signals broadcast by the respective cell groups, and has echoes in the contrasting roles of GABA and VIP in sculpting the phase‐distribution across the SCN during re‐entrainment to photoschedules (Evans *et al*, 2013). The closer phasing of *Prok2* ^+^ cells and *ProkR2* ^+^ cells compared to the *Vip* ^+^ and *Vipr2* ^+^ cellular pair (0.6 h versus 1.7 h) may underly this difference in phase‐control across the “driven” circuit: Prok2 signalling mediating robustness and tight coupling as an “integrator” hub, whilst the VIP‐mediated distributor hub determines cellular and ensemble phases.

In conclusion, the current results reveal the cellular nodes of the SCN circuit and provide a framework to understand its topology, a feature that is subject to transcriptional assembly/disassembly across circadian time. They also underline *Prok2* ^+^/*ProkR2* ^+^ cells as a neurochemically, topologically and functionally distinct cellular axis that is able to determine the emergent properties of the SCN, but is nevertheless different from the far better characterised *Vip* ^+^/*Vipr2* ^+^ axis. In extrapolating topological organisational principles of the SCN, we highlight a structure consisting of divergent “distributor” VIP/VPAC2 and GRP/GRPR nodes, and “integrator” Prok2/ProkR2 and AVP/AVPR1A nodes, which is further supported by our functional observations of VIP/VPAC2 and Prok2/ProkR2 signalling axes differentially routing SCN network communication. When compared to each other, the Prok2/ProkR2 populations appear more powerful in setting ensemble period, yet VIP/VPAC2‐initiated network‐wide SCN rhythms stereotypically show organised sequential phase activation that Prok2/ProkR2‐initiated SCN lack. We propose that this inter‐nested, multi‐layered network structure is the origin of the remarkable time‐keeping power of the SCN. Future work in functionally annotating additional cellular nodes identified by our transcriptomic study will aid in further unravelling how information flows through this uniquely effective time‐keeping nucleus. This analysis may also increase our understanding of network principles shared across domains that computationally shape emergent properties of cellular circuits.

## Materials and Methods

### Reagents and Tools table

**Table:** 

Reagent or Resource	Source	Identifier
**Antibodies**		
Anti‐Prok2	Abcam	Cat# ab76747; RRID: AB_1524238
Anti‐ProkR2	Alomone Labs	Cat #APR‐042
Alexa Fluor 488 Goat Anti‐Rabbit antibody	Thermo Fisher Scientific	A‐11008; RRID: AB_143165
Alexa Fluor 568 Goat Anti‐Rabbit antibody	Thermo Fisher Scientific	A‐11011; RRID: AB 143157
Vectashield HardSet Mounting Medium with DAPI	Vector Laboratories	Cat# H‐1500; RRID:AB_2336788
**Deposited Data**		
AAV1.pProk2. Cre.T2A.mCherry	VectorBuilder	#169013
AAV1.pProkr2. Cre.T2A. Venus	VectorBuilder	#169014
Mouse suprachiasmatic nucleus scRNASeq data	This paper	GSE167927
**Chemicals, Peptides, and Recombinant Proteins**		
Triton x‐100	Sigma Aldrich	Cat #X100
Eagle’s basal medium	Sigma Aldrich	Cat# B1522
Dulbecoo Modified Eagle Medium	Sigma Aldrich	Cat# D5796
B27	GIBCO	Cat# 17504‐044
HEPES	Sigma Aldrich	Cat# H0887
FCS	Sigma Aldrich	Cat# 59665
EBSS	GIBCO	Cat# 24010043
D‐glucose	Fisher	Cat# 10373242
Glutamax	GIBCO	Cat# 35050061
MK801	Sigma Aldrich	Cat# M107
APV	Sigma Aldrich	Cat# A5282
D‐MEM	Sigma Aldrich	Cat# D5030
NaHCO3	Fisher	Cat# 10553325
Heat inactivated horse serum	GIBCO	Cat# 16050130
Recombinant Prokineticin 2	Sigma Aldrich	Cat# SRP3146
Recombinant PKRA7	Tocris, Biotechne	Cat# 6238
**Critical Commercial Assays**		
10× Genomics Chromium Single Cell Library Kit v2	10× Genomics	Cat# 120234
RNAscope Fluorescent Multiplex	Advanced Cell Diagnostics	Cat# 320850
RNAscope Probe‐ Mm‐ Prok2	Advanced Cell Diagnostics	Cat# 447941
RNAscope Probe‐ Mm‐ Avp	Advanced Cell Diagnostics	Cat# 401391
RNAscope Probe‐ Mm‐ Avp‐C2	Advanced Cell Diagnostics	Cat# 401391‐C2
RNAscope Probe‐ Mm‐Prokr2‐C3	Advanced Cell Diagnostics	Cat# 498431‐C3
RNAscope Probe‐ Mm‐Vipr2‐C2	Advanced Cell Diagnostics	Cat# 465391‐C2
RNAscope Probe‐ Mm‐Avpr1a‐C2	Advanced Cell Diagnostics	Cat# 418061‐C2
Worthington Papain dissociation kit	Worthington/ Lorne Labs	Cat# LK003150
**Experimental Models: Organisms/Strains**		
PER2::LUC (B6.129S6‐Per2tm1Jt/J)	Jax Laboratories	RRID:IMSR_JAX:006852
Cry1^−/−^, Cry2^−/−^	Van der Horst *et al*, 1999	N.A.
**Recombinant DNA**		
pAAV. Syn.NES.jRCaMP1a.WPRE.SV40	Addgene	RRID : A ddgene_100848
pAAV. Syn.GCaMP6f.WPRE.SV40	Addgene	RRID : A ddgene_100837
pAAV‐EF1a‐DIO‐Chr2(H113R)‐EYFP‐HGHpA	Addgene	RRID : A ddgene_20298
**Software and Algorithms**		
Cell Ranger analysis pipeline v2.0	10× Genomics	https://support.10xgenomics.com/single‐cell‐gene‐expression/software/pipelines/latest/what‐is‐cell‐ranger; RRID:SCR_017344
Loupe Browser	10× Genomics	https://support.10xgenomics.com/single‐cell‐gene‐expression/software/visualization/latest/what‐is‐loupe‐cell‐browser; RRID:SCR_018555
STAR aligner v2.5.2b	Dobin *et al*, 2013	STAR; RRID:SCR_015899
BioDare	Moore *et al*, 2014	https://www.biodare.ed.ac.uk
GraphPad Prism 8.0	GraphPad Software	http://www.graphpad.com/; RRID:SCR_002798
R version 3.6.1	R Foundation for Statistical Computing, Vienna, Austria	http://www.R‐project.org; RRID:SCR_001905
R package “circular” version 0.4‐93	Comprehensive R Archive Network	https://r‐forge.r‐project.org/projects/circular
R package “ggplot2” version 3.1.2 and “scales” version 1.0.0	Comprehensive R Archive Network	https://cran.r‐project.org/web/packages/ggplot2/index.html; RRID:SCR_014601
R package “RColorBrewer” version 1.1‐2	Comprehensive R Archive Network	http://colorbrewer2.org/
RStudio version 1.2.1335	RStudio Team, Boston, MA	http://www.rstudio.com; RRID:SCR_000432
Matlab R2020b	MathWorks Inc, USA	http://www.mathworks.com/products/matlab/; RRID:SCR_001662
Matlab Toolbox “CircularGraph” version 2.0.0.0	P. Kassebaum (GitHub)	Retrieved February 3, 2021; https://github.com/paul‐kassebaum‐mathworks/circularGraph
FIJI/ImageJ	Schindelin *et al*, 2012	https://imagej.net/Fiji; RRID:SCR_002285
ZEN Blue	Zeiss	RRID :S CR_013672
Adobe Illustrator CC	Adobe	RRID :S CR_010279

### Methods and Protocols

#### Experimental model and subject details

##### Mice

All experiments were performed on healthy mice, with normal immune status, housed in a specific pathogen free (SPF) unit (Ares Facility, Babraham Institute Campus, Cambridge, UK). Experimental subjects were not involved in any previous test or drug treatment. For scRNASeq and *ex vivo* SCN slice experiments, both female and male pups were used. Pups were maintained in a 12:12 light‐dark cycle together with their mothers before being sacrificed at P10‐12. SCN material used for scRNASeq at CT15.5 came from pups housed in a reversed 12:12 lighting schedule. Food and water were provided *ad libitum*. All animal work was conducted and licensed in accordance with the Code of Practice for the Housing and Care of Animals Bred, Supplied or Used for Scientific Purposes under A(SP)A and the EU Directive 2010/63/EU, and with local ethical approval (MRC‐LMB AWERB). PER2::LUC mice (allele: *B6.129S6‐Per2tm1Jt*/*J*) were a gift from J.S. Takahashi (UT Southwestern, US). Cryptochrome‐null mice were derived from founders provided by G van der Horst (Erasmus University Medical Centre, Rotterdam, NL). All lines were maintained on a C57BL/6J background with required genotypes bred in‐house by inter‐crossing the lines.

##### Experimental design

All *ex vivo* experiments were performed on at least five animals. scRNASeq experiments were performed on five independent cohorts of mice. Number of experimental replicates (*n*) is indicated in figure legend and text and refers to the number of animals used independently treated in each experimental condition. Animals were selected in an unblended manner, but no specific randomisation strategy was used. Statistical computations were not performed to determine the optimal sample size for experiments. Data from all the experiments were included in the analysis, with the only exclusion of SCN slices being those that died for unrelated technical reasons (e.g. inadequate seal of the glass cover on petri dishes).

##### SCN explant culture, bioluminescence and fluorescence imaging

Brains were retrieved from mouse pups at P10‐12 and cut into 300‐µm‐thick slices using a McIlwain Tissue Chopper. The slices were examined under a bright‐light microscope to sort those containing optimal SCN preparations and trimmed accordingly. Trimmed SCN slice preparations were transferred to Millipore membrane filters (Millipore, USA) and placed inside 35‐mm‐diameter dishes with culture medium containing: 50% v/v Eagle’s basal medium (Sigma, USA), 25% v/v EBSS (Gibco, USA), 25% v/v heat inactivated horse serum (Invitrogen, USA), 5 mg/ml D‐Glucose (Fisher, USA), 1% glutamax (Invitrogen, USA), 100 nM MK801 (Sigma, USA) and 3 mM MgCl_2_ and 0.05 mM APV (Sigma, USA).

For real‐time bioluminescent recordings of PER2 expression, organotypic SCN slices were transferred to 35‐mm‐diameter dishes with air medium solution containing: 8.3 mg/ml DMEM (Sigma, USA), 0.35 mg/mK NaHCO3 (Fisher, USA), 5 mg/ml d‐glucose (Fisher, USA), 0.01 M HEPES (Sigma, USA) and 100 µM luciferin (Promega, USA). Dishes were air‐sealed by lining the rim with silicon grease. Photomultiplier tubes (PMTs) were used to detect luciferase‐emitted photons.

Single SCN cells were isolated for transcriptional profiling by proteolytic enzymatic digestion of pooled organotypic SCN slices using the Worthington Papain dissociation kit (Lorne Laboratories, UK). Sterile procedures were maintained throughout the manufacturer’s protocol. Each of 16‐20 SCN slices that were pooled per sequencing run into a single sample were first imaged on an EVOS microscope to ensure healthy sample tissue. The slices were gently removed from their membrane supports, pooled into one Falcon tube and incubated in 1 mM l‐cysteine/0.5 mM EDTA activated papain solution (Worthington) under constant agitation for 90 min. Dissociated cells were centrifuged at 300 *g* for 5 min at room temperature and dual‐filtered through an ovomucoid gradient (Worthington) and a 40‐µm strainer (Corning, UK) to avoid doublets.

##### ScRNASeq

scRNASeq libraries were prepared using the Chromium Single Cell Controller (10× Genomics, Pleasanton, CA). Both biochemical and bioinformatic quality control measures were performed to ensure that the average size and cDNA concentration satisfied quality thresholds and that the spread of the sequenced reads across genes was even across single cells (i.e. a normal distribution of genes with at least one UMI tag). Cells were treated following the manufacturer’s protocols using 10× Genomics ChromiumTM Single Cell 3′ Library and Gel Bead Kit v2 (120267) and ChromiumTM Single Cell A Chip Kit (1000009). SCN cells, diluted in 0.04% BSA‐nuclease‐free water, reverse transcription master mix and partitioning oil, were loaded on a single cell chip and run on the Chromium Controller to obtain a target cell recovery rate of 8,000 cells. Reverse transcription was performed within the droplets at 53°C for 45 min. cDNA was amplified for 12 cycles total on a Bio‐Rad C1000 Touch thermocycler. A qualitative analysis of the amplified cDNA was run on the Agilent TapeStation High Sensitivity D1000 ScreenTape to determine the average size and cDNA concentration. cDNA was fragmented using the proprietary fragmentation enzyme blend for 5 min at 32°C, followed by end repair and A‐tailing at 65°C for 30 min. Sequencing adaptors were ligated to the cDNA at 20°C for 15 min. Final libraries were sequenced on one flow cell of an Illumina HiSeq 4000 (Illumina, San Diego) with a read length of 26 bp for read 1 (cell barcode and unique molecule identifier (UMI) and i7 index read (sample barcode)) and 98 bp for read 2 (RNA read) to yield approximately 340 million reads per sample (across the runs sequencing at CT7.5 our depth was 83,220 ± 15,783 reads/cell, mean ± SEM, and across those at CT15.5, we obtained 43,231 ± 14,291 reads/cell). To ensure high‐quality data were taken for further analysis, only genes expressed in three or more cells, and cells with at least 100 detected genes, were retained in the dataset. A verification step in Seurat was performed to ensure all mitochondrial transcript‐expressing cells were removed from the dataset during Cell‐ranger processing.

##### AAV particles design and production

Plasmids encoding for *Cry1*‐Flex‐Cry1‐Luc and *Cry1*‐Flex‐Cry1::EGFP were produced in‐house as described in (Brancaccio *et al*, 2019). Briefly, the Cry1 promoter was PCR‐amplified from mCry‐Cry1::EGFP and cloned into pAAV‐EF1a‐DIO‐Chr2(H113R)‐EYFP‐HGHpA (Addgene 20298) by using MluI and EcoRI sites. AAV‐pCry1‐DIO‐ChR2‐YFP was then used to produce both mCry1‐Luc and mCry1‐Cry1::EGFP. Plasmids were packaged into AAVs by Penn Vector Core (University of Pennsylvania). Plasmids encoding AAV1.pProk2. Cre.T2A.mCherry and AAV1.pProkR2. Cre.T2A. Venus were designed by A. A. For the pProk2 promoter sequence, a 400‐bp region upstream of the gene encompassing histone post‐translational modifications and brain‐associated DNase hypersensitive regions was incorporated. For the pProkR2 promoter sequence, the upstream brain‐associated DNase hypersensitive sites corresponding to its untranslated exon 1 were contracted with the conserved first coding exon untranslated regions. Plasmids were constructed and packaged into AAVs by VectorBuilder (vectorbuilder.com), details, maps and plasmids available at Addgene (addgene.org): AAV1.pProk2. Cre.T2A.mCherry Plasmid #169013 and AAV1.pProkR2. Cre.T2A. Venus Plasmid #169014.

##### AAV transduction of SCN slices

SCN organotypic slices from P10‐12 mice were obtained, cultured and transduced as previously described (Brancaccio *et al*, 2013). The calcium reporter AAVs *pSyn*‐NES.jRCaMP1a.WPRE.SV40 (Addgene viral prep 100848‐AAV1; http://n2t.net/addgene:100848; RRID:Addgene_100848) and *pSyn*‐GCaMP6f.WPRE.SV40 (Addgene viral prep 100837‐AAV1; http://n2t.net/addgene:100837; RRID:Addgene_100837) were gifts from Douglas Kim & GENIE project and purchased from Addgene as AAV serotype 1.

For AAV transduction, slices were briefly taken out from the PMT tubes and immediately returned for PMT recording, with no medium change. Dynamic changes in PER2::LUC signal were recorded in real‐time for > 7 days; no further treatment was performed. No phenotype was generally observed during the first 4 days post‐transduction, consistent with time required for AAV infection cycle (Edwards *et al*, 2016). After that, a phenotype became evident and was assessed from day 4 to day 8 post‐transduction. Transduction efficiency was verified at the end of the experiment by assessing number of mCherry^+^ or Venus^+^ cells/SCN area.

##### Drug treatments

Recombinant Prok2 was purchased from Sigma Aldrich (#SRP3146) and PKRA7 antagonist was purchased from Tocris Bioscience (#6238). Prok2 and PKRA7 were diluted in air medium containing no luciferin, VIP was diluted in HEPES‐buffered medium and air medium without luciferin was used as vehicle controls. For washout, slices were transferred to fresh medium containing luciferin. SCN slices were kept in standard culture medium (DMEM based with 5% serum and glutamax supplement 1× (Life Technologies).

##### Immunofluorescence on SCN slices

Antiserum for immunofluorescence on SCN slices: anti‐rabbit Prok2 (Abcam, #ab76747), anti‐rabbit ProkR2 (Alomone, #APR‐042); secondary antibodies: goat anti‐rabbit conjugated with Alexa 488; goat anti‐rabbit conjugated with Alexa 568 (Life Technologies). Targeting rates and co‐localisation analysis of antisera with fluorescently labelled AAVs of co‐transduced SCN slices were manually performed on single confocal planes (ImageJ, USA) (Schindelin *et al*, 2012).

##### Multiplexed long‐term live‐imaging

Multiplexed bioluminescence/fluorescence imaging was performed as previously described (Brancaccio *et al*, 2013), using the LV200 system (Olympus Microscopy, UK). Briefly, SCN explants were sealed into 35 mm dishes with glass bottoms (Mattek, Slovakia) and transferred to the heated stage of an LV200 microscope system equipped with an EM‐CCD camera (Hamamatsu, Japan). For combined bioluminescence and fluorescence, images were taken at a time resolution of 30 min. Fluorescence exposure was 100 ms, and bioluminescence signal was acquired over 29.5 min.

##### Immunohistochemistry

Brains were retrieved from 4% PFA perfused adult mice and left in 4% PFA for a further 4 h before cryopreservation in 20% sucrose (in 0.1 M PBS). Fixed brains were trimmed and aligned on a freezing microtome stage, sectioning the brain rostral to caudal at 40‐µm intervals. SCN sections were washed twice in PBS, before incubating for 15 min in 50 mM glycine solution. Sections were then pre‐blocked in 3% Triton X‐100, 5% normal goat serum prepared in PBS solution for 60 min and subsequently blocked in 0.3% Triton X‐100, 1% BSA, 5% normal goat serum in PBS solution for a further 60 min. Sections were incubated for 24 h in primary antibody, washed twice in 0.1% Triton X‐100, 0.3% BSA–PBS solution and incubated for 2 h with secondary antisera at room temperature. The immunostained brain sections were mounted on slides (Superfrost, Fisher, USA) using Vectashield with DAPI (Vectalabs) to support fluorophore signal retention.

##### In situ hybridisation of tissue sections

Brains dissected from 7‐month‐old C57BL/6 mice were embedded in OCT medium and frozen at −80°C. 14‐µm sections were prepared for RNAscope (ACD Inc.) in situ hybridisations (ISH). SCN sections on slides were submerged in 4% pre‐chilled PFA for 30 min and subjected to dehydration using an ethanol series of 50, 70 and 100% ethanol solutions with slides being immersed for 5 min in each solution. A hydrophobic barrier around the tissue section was created using the ImmEdge hydrophobic barrier pen (ACD Inc.) to localised applied solution to the sample. RNAScope Protease IV was topically applied to each section and incubated for 30 min at room temperature. C1‐, C2‐ and C3‐conjugated RNAscope probes were applied in a 50:1:1 ratio to detect *Prok2* (#447941), *Avp* (#401391/#401391‐C2), *ProkR2* (#498431‐C3), *Vipr2* (#465391‐C2) and *Avpr1a* (#418061‐C2).

##### Confocal imaging

ISH imaging was carried out using a Zeiss 710 confocal microscope with spectral detector using a 40×/1.1NA oil objective. Alexa 488 was stimulated with a 488 nm laser, and 546 was stimulated with a 561 laser. For detection of single transcripts of weakly expressed genes, imaging was performed with a 63×/1.1NA long oil immersion objective (Zeiss LD C‐Apochromat). Spectral unmixing was performed using Zeiss ZEN software.

#### Quantification and statistical information

##### scRNASeq

Base calls were converted to reads with the software Cell Ranger (10× Genomics; version 2.1) mkfastq. These reads were then aligned against the mouse reference (GENCODE Mouse Release 26 (GRCm38)) using the Cell Ranger 2.1.0 pipeline (an implementation of STAR v2.7.0, 10× Genomics) with SC3Pv2 chemistry and 5,000 expected cells per sample (Dobin *et al*, 2013). Cell barcodes representative of quality cells were delineated from barcodes of apoptotic cells or background RNA based on a threshold of having at least 200 unique transcripts profiled, < 10,000 total transcripts and less than 10% of their transcriptome of mitochondrial origin. Potential multiplets were classified as outside three median absolute deviations (MADs) for percentage mitochondrial content, number of genes and number of UMIs and removed. UMIs from each cell barcode were retained for all downstream analysis. Raw UMI counts were normalised with a scale factor of 10,000 UMIs per cell and subsequently natural log transformed.

Clustering of cells based on transcriptome similarities was performed by either graph‐based clustering or k‐means clustering methods (Cell Loupe Browser, 10× Genomics).

##### PMT recordings

Data analyses of period, amplitude and goodness‐of‐fit (GOF) were performed manually or by using the FFT‐NLLS function of the online BioDare suite (Moore *et al*, 2014; Zielinski *et al*, 2014) (https://www.biodare.ed.ac.uk) (Courtesy of Prof. Andrew Millar, University of Edinburgh). For phase maps, a threshold was applied to the bulk‐emitted signal to remove extra‐SCN signals present in organotypic slices. A continuous grid of ROIs (custom Fiji plugin) was overlaid, and signals extracted from ROIs considered of SCN origin were detrended and smoothed using a 2.5‐h moving average. The ROI signals were then analysed using the BioDare FFT‐NLLS algorithm. ROIs were considered initiated if they passed two tests: (i) the period of the fit was between 23.5 and 34.5 h, and (ii) the relative amplitude error (RAE) of the fit was < 0.3. Continuous phase maps and maps of initiated versus non‐initiated ROIs were generated from BioDare output data in RStudio using the ggplot2, scales and RColorBrewer packages. For comparison across different SCN slices, one‐way ANOVA with Sidak’s multiple comparisons test or two‐way ANOVA with Tukey’s multiple comparisons test in GraphPad Prism 8 (GraphPad) were used to assess statistical significance. Phase coherence was assessed by applying a Rayleigh test to ROI data. Circular Rayleigh plots to illustrate this dispersal were generated using the R package circular (version 0.4‐93, R package “circular”: Circular Statistics, https://r‐forge.r‐project.org/projects/circular) in R (version 3.6.1, courtesy of R Foundation for Statistical Computing, Vienna, Austria, http://www.R‐project.org) and RStudio (version 1.2.1335, courtesy of RStudio Team, Boston, MA. http://www.rstudio.com). Rayleigh vectors were plotted on top of phase distributions in GraphPad Prism 8 (GraphPad).

## Author contributions

ELM and MHH designed research; AA designed and provided AAV reagents; ELM performed and analysed research except for LV200 recordings, which were performed and analysed by AP and mouse stereotaxic injections performed by JEC; APP contributed to project discussions; and AC guided analysis of scRNASeq data. ELM and MHH wrote the paper.

## Conflict of interest

The authors declare that they have no conflict of interest.

## Supporting information



Appendix

Expanded View Figures PDF

Source Data for Expanded View and Appendix

Source Data for Figure 1

Source Data for Figure 2

Source Data for Figure 3

Source Data for Figure 4

Source Data for Figure 5

Source Data for Figure 6

Source Data for Figure 7

Source Data for Figure 8

Source Data for Figure 9

## Data Availability

The datasets and computer code produced in this study are available in the following databases:
ScRNASeq data: NCBI Gene Expression Omnibus with the accession number: GSE167927 (https://www.ncbi.nlm.nih.gov/geo/query/acc.cgi?acc=GSE167927). Codes used to analyse scRNASeq data are publicly available from 10× Genomics at “https://support.10xgenomics.com/single‐cell‐gene‐expression/software/overview/welcome.” Viral reagents generated in this study have been deposited with Addgene, AAV1.pProk2.Cre.T2A.mCherry Plasmid #169013 and AAV1.pProkR2.Cre.T2A.Venus Plasmid #169014.
