# Supplementary material for: Single‐cell transcriptomics of suprachiasmatic nuclei reveal a Prokineticin‐driven circadian network
Source: EMBO J. 2021 Sep 6;40(20):e108614. doi: 10.15252/embj.2021108614 (PMC8521297; doi:10.15252/embj.2021108614)
Supplement: Supplementary file 1 — Appendix [file EMBJ-40-e108614-s010.pdf]

Appendix

Table of Contents:

Appendix Figure S1: Raw Per2::Luciferase traces of SCN slices harvested in parallel to slices used for scRNASeq, related to Figure 1

Appendix Figure S2: Gene Ontology analysis of the transcriptional profiles of day versus night sequenced SCN cells, related to Figure 2

Appendix Figure S3: Raw bioluminescent traces of SCN used to generate a phase response curve for Prok2 treatment, related to Figure 6

Appendix Figure S4: Raw bioluminescent traces of SCN treated with ProkR2 antagonist and corresponding controls, related to Figure 7

Appendix Figure S1. Raw PER2::LUC traces of SCN slices harvested in parallel to slices used for scRNASeq.

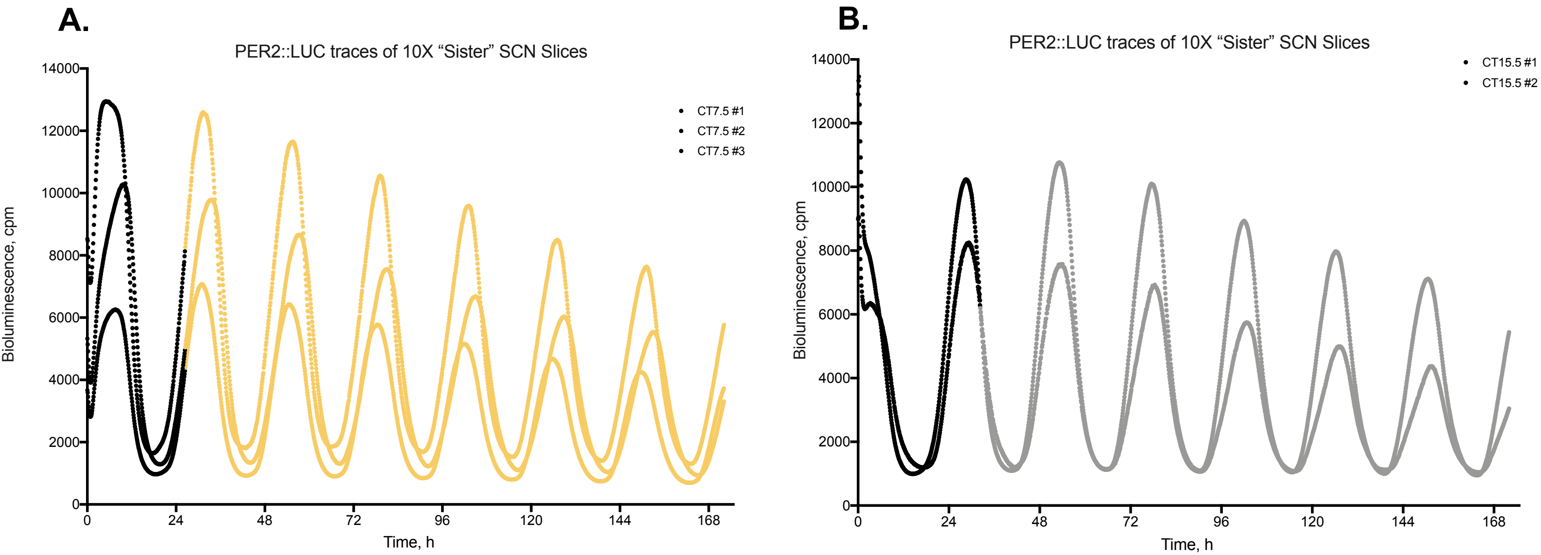

Appendix Figure S1. Raw PER2::LUC traces of three representative SCN slices harvested in parallel to slices sequenced at CT7.5. The transition from black to yellow indicates when corresponding slices were taken for scRNASeq. B. Raw Per2::Luciferase traces of two representative SCN slices harvested in parallel to slices sequenced at CT15.5.

Appendix Figure S2: Gene Ontology analysis of the transcriptional profiles of day versus night sequenced SCN cells

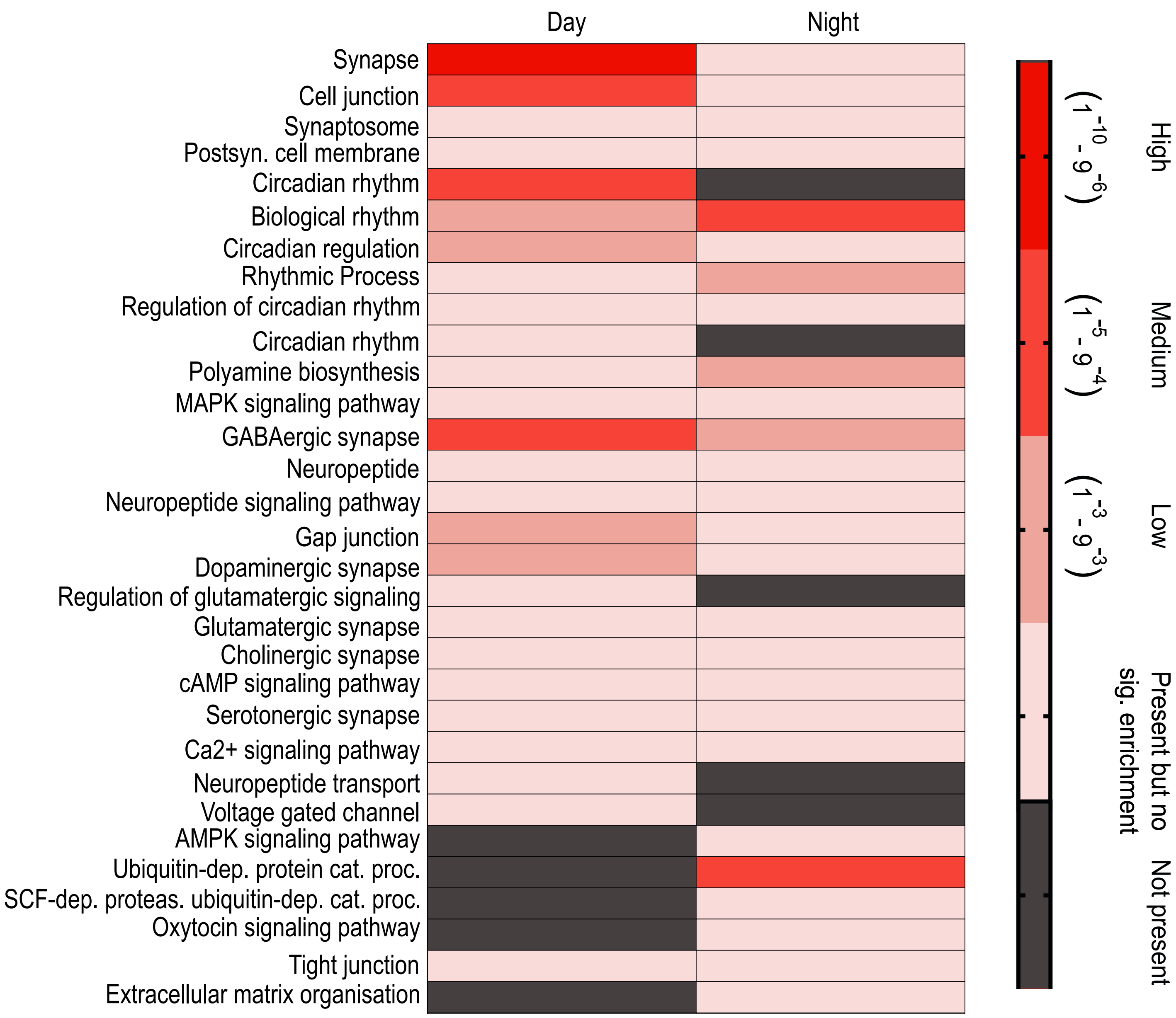

Appendix Figure S2: Gene Ontology term functional annotation of the transcriptional profiles of CT7.5 (day) and CT15.5 (night) SCN cells. The intensity of red indicates increased enrichment of genes associated with the respective functional annotations. Grey is used when no genes associated with the respective annotation is found in the particular dataset.

Appendix Figure S3. Raw bioluminescent traces of SCN used to generate a phase response curve for Prok2 treatment, Related to Figure 6:

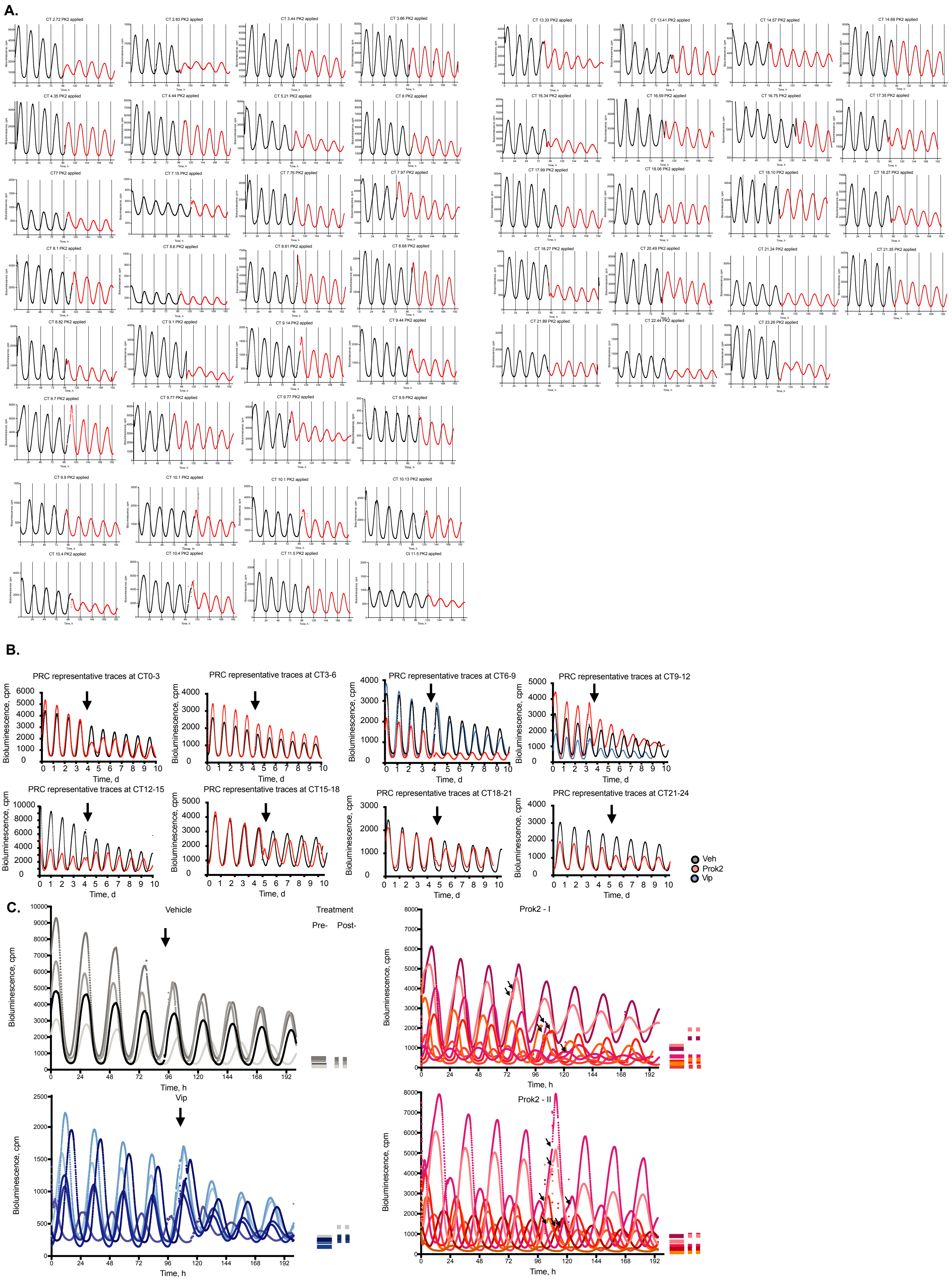

Appendix Fig. S3A. Raw bioluminescent traces of Per2::Luciferase SCN slices before (black) and after (red) Prok2 treatments across circadian time. **B.** Raw bioluminescent traces of Per2::Luciferase SCN slices before and after (as depicted by the black arrow) vehicle (black trace), Prok2 (red trace) or VIP (blue trace) treatments. Within each 3hr time-window a representative trace from each cohort is depicted (VIP was only applied between CT7 and 12). **C.** All traces from each respective cohort that were treated between CT9 and 12 are shown in cohort-specific graphs. To the right of the bioluminescent traces solid bars for each slice indicate the level of base-line Per2::Luciferase colour-matched with the

Appendix Figure S4. Raw bioluminescent traces of SCN treated with ProkR2 antagonist and corresponding controls, Related to Figure 7:

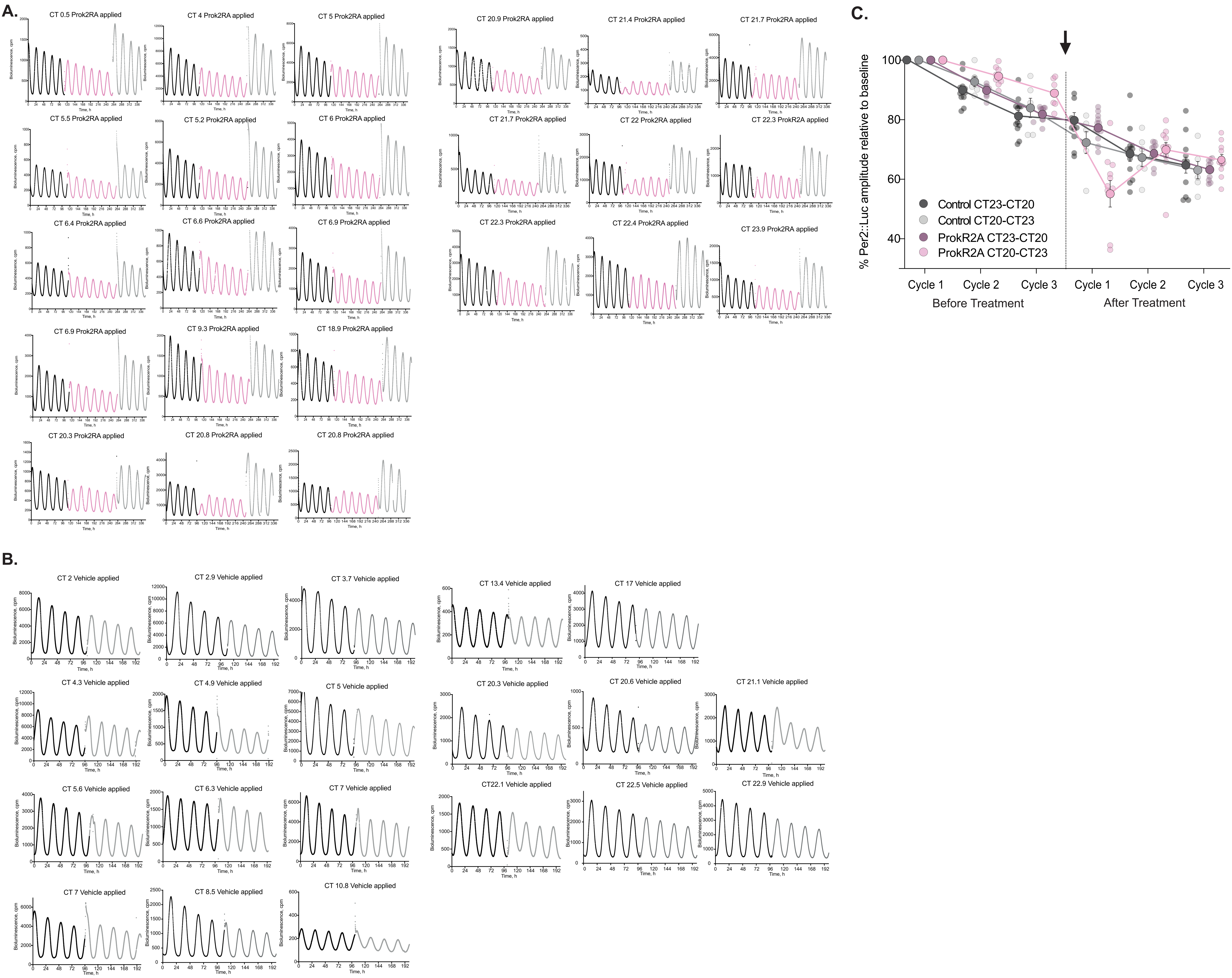

Appendix Fig. S4A. Raw bioluminescent traces of Per2::Luciferase SCN slices before (back), after Prok2RA (pink) and washout (grey). **B.** Raw bioluminescent traces of Per2::Luciferase SCN slices before (black) and after vehicle (grey) treatments across circadian time. **C.** Change in Per2 amplitude across three cycles preceding and succeeding vehicle (grey) or Prok2RA (pink) treatment. Vehicle and Prok2RA cohorts are further split in CT23-20 (dark grey vehicle *n*=12 and pink Prok2RA *n*=13, respectively) or CT20-23 treated (crossed light grey vehicle *n*=6 and pink Prok2RA *n*=11). Individual slices are depicted as individual points which are offset for clarity. A significant dip in amplitude is seen when Prok2R signalling is blocked between CT20-23 ($p<0.0001$).
