# Supplementary material for: Single‐cell transcriptomics of suprachiasmatic nuclei reveal a Prokineticin‐driven circadian network
Source: EMBO J. 2021 Sep 6;40(20):e108614. doi: 10.15252/embj.2021108614 (PMC8521297; doi:10.15252/embj.2021108614)
Supplement: Supplementary file 13 [file EMBJ-40-e108614-s005.pdf]

THE EMBO JOURNAL

Single-cell transcriptomics of suprachiasmatic nuclei reveal a Prokineticin-driven circadian network

Emma Morris, Andrew Patton, Johanna Chesham, Alastair Crisp, Antony Adamson, and Michael Hastings
DOI: 10.15252/embj.2021108614

Corresponding author(s): Michael Hastings (mha@mrc-lmb.cam.ac.uk)

Review Timeline:

Submission Date:	30th Apr 21
Editorial Decision:	16th Jun 21
Revision Received:	5th Jul 21
Editorial Decision:	27th Jul 21
Revision Received:	5th Aug 21
Accepted:	9th Aug 21

Editor: Karin Dumstrei

Transaction Report:

(Note: With the exception of the correction of typographical or spelling errors that could be a source of ambiguity, letters and reports are not edited. Depending on transfer agreements, referee reports obtained elsewhere may or may not be included in this compilation. Referee reports are anonymous unless the Referee chooses to sign their reports.)

1st Editorial Decision 16th Jun 2021

Dear Dr. Hastings,

Thank you for submitting your manuscript to The EMBO Journal. Your study has now been seen by three referees and their comments are provided below.

As you can see, the referees find the analysis interesting and insightful. Referees #1 and 2 raise relative minor concerns that are easy to sort out. The main concerns raised by referee #3 are regarding using organotypic SCN brain slices for scRNAseq and collecting only two circadian time points (CT7.5 and CT15.5). Regarding the use of organotypic slices for scRNAseq: did you do any validation to look at RNA degradation or any other validation that can be used to address this point? Regarding the two timepoints, I think that is fine - more would have been better, but it is fine as is. I also don't see major issues with novelty as pointed out by this referee.

I would therefore like to invite you to submit a revised version. I think it would be helpful to discuss the revisions needed further I am happy to do so via email or video.

When preparing your letter of response to the referees' comments, please bear in mind that this will form part of the Review Process File, and will therefore be available online to the community. For more details on our Transparent Editorial Process, please visit our website: https://www.embopress.org/page/journal/14602075/authorguide#transparentprocess

Thank you for the opportunity to consider your work for publication. I look forward to discussing your revisions further.

I have attached a document with helpful tips on how to prepare the revised version. Please pay attention to the part on the Data Availability Section and figure legends (to include stats and replicate info in the legends).

with best wishes

Karin

Karin Dumstrei, PhD
Senior Editor
The EMBO Journal

Instructions for preparing your revised manuscript:

Please make sure you upload a letter of response to the referees' comments together with the revised manuscript.

Please also check that the title and abstract of the manuscript are brief, yet explicit, even to non-specialists.

When assembling figures, please refer to our figure preparation guideline in order to ensure proper formatting and readability in print as well as on screen:

https://bit.ly/EMBOPressFigurePreparationGuideline

IMPORTANT: When you send the revision we will require
- a point-by-point response to the referees' comments, with a detailed description of the changes made (as a word file).
- a word file of the manuscript text.
- individual production quality figure files (one file per figure)
- a complete author checklist, which you can download from our author guidelines (https://www.embopress.org/page/journal/14602075/authorguide).
- Expanded View files (replacing Supplementary Information)
Please see out instructions to authors
https://www.embopress.org/page/journal/14602075/authorguide#expandedview

Please remember: Digital image enhancement is acceptable practice, as long as it accurately represents the original data and conforms to community standards. If a figure has been subjected to significant electronic manipulation, this must be noted in the figure legend or in the 'Materials and Methods' section. The editors reserve the right to request original versions of figures and the original images that were used to assemble the figure.

Further information is available in our Guide For Authors:
https://www.embopress.org/page/journal/14602075/authorguide

Revision to The EMBO Journal should be submitted online within 90 days, unless an extension has been requested and approved by the editor; please click on the link below to submit the revision online before 14th Sep 2021:

https://emboj.msubmit.net/cgi-bin/main.plex

Referee #1:

Mammalian circadian rhythms are generated by a network or 20,000 neurons in the SCN. Whilst intracellular mechanisms are critical for this process, network activity of the SCN also plays a key role. Despite great advances in our understanding of circadian rhythms, how different cellular populations within the SCN contribute to this process is poorly understood. In this paper, the authors use single cell RNAseq (scRNAseq) of the SCN, identifying 11 distinct neuronal subpopulations. They go on to define neuropeptidergic signalling between these neuronal subtypes, highlighting a key role for prokineticin 2 (Prok2) and its receptor ProkR2, which undergo dramatic differences in inter-cluster signalling between day and night. Using cell-specific viral vectors as well as Prok2/Prok2R pharmacological tools, they assess the role of Prok2/ProkR2 in the regulation of circadian rhythms in the SCN. This includes restoring evaluating the effects on overt periodicity by restoring Cry1 expression in just the Prok2/ProkR2 cells as well as investigating whether Prok2/ProkR2 cells alone can drive SCN rhythms by restoring Cry1 expression in these neurons in an arrhythmic SCN.

Together, this manuscript reflects a remarkable body of work which greatly advances our

understanding of the role of specific cellular populations in the SCN in the generation of biological time. Whilst Prok2 has been known to be play a role in the SCN for over ten years, its precise contribution to the generation and maintenance of circadian rhythms was poorly understood. This role is actually quite subtle and complex, and via the use of a series of elegantly-designed experiments, this paper greatly advances this understanding. This work provides a very valuable extension of our understanding of the role of specific neuronal populations and signalling within the SCN. Specific comments are provided below.

Main comments

1. Methodological justification. Why were the specific timepoints chosen (CT7.5 and 15.5)? This is not clearly explained in the manuscript.
2. Expression data for neuronal subtypes. In figure 2B, day vs night enriched genes are shown. I may have missed this, but is a breakdown of these genes by the identified neuronal subtypes included? Are day/night differences selectively enriched in specific cell types? Relating to this, the inclusion of the raw data for each figure is very helpful. However, are full transcriptomic data (all expressed genes) available for each of the specific neuronal subpopulations? This would an incredibly valuable resource for any lab working on gene expression in the SCN, as it will allow whole SCN datasets to be reanalysed with regard to the specific neuronal subtypes identified in this study (without having to download and reanalyse all scRNA data, which would be a significant undertaking). This would provide significant additional added value and be a very useful resource. For example, if gene X is identified as being of interest in a whole SCN study, is its expression confined to a specific neuronal subtype within the SCN?
3. Behavioural predictions. Conducting in vivo behavioural evaluation of the Prok2/ProkR2 axis would be challenging and would presumably take a whole new paper to investigate. However, it would be helpful to extend the discussion to relate the observed circadian and sleep phenotypes of Prok2/ProkR2 mice (e.g. Li et al., 2007; Prosser et al., 2007; Hu et al., 2007) to the new insights provided by this study. For example, the precision of timing, the redistribution of activity, altered body temperature, glucocorticoid rhythms and sleep. Do the current results provide any insight into why entrainment to light may be unaffected (e.g. could light input primarily involved different neuronal subtypes?). What would be the prediction regarding conditional knockout of Prok2/ProkR2 in the SCN? And would the loss of Prok2/ProkR2 be expected to result in an SCN that is more prone to environmental disruption? In hindsight, one advantage of this study was that samples were taken from SCN slices, so the patterns of gene expression observed could not be affected by feedback from behavioural state.

Minor comments

1. It may be a journal requirement, but the very short figure legends did not help with a detailed comprehension of data presented in the figures. More information in these figure legends would make this much more accessible.
2. Presumably the whole trimmed SCN slices were used for the scRNAseq? Presumably the surrounding tissue accounts for the 'extra-SCN hypothalamic neurons'
3. I was unclear how single cell dispersions were made from circa 17 SCN slices (page 4).
4. In Figure 2 is the criteria of $p<0.05$ based on FDR corrected p-values?
5. In Figure 3, the 8 neuronal clusters are classified using several neuropeptidergic identifiers. I was unclear why these specific genes were used as identifiers? Presumably other marker genes from each cluster could have been used? Were these chosen due to previously characterised SCN expression? Or were they the most unique identifiers of each cluster (and if so, based on what metric)? Whilst this is not a limitation in any way, the rationale for classification here could be made

clearer.
6. More information regarding the meaning and interpretation of the signalling topology maps shown in Figure 4 would be helpful.
7. I found the classification of subtypes by neuropeptides and their receptors very informative. Visualisation with related colours for each neuropeptide and receptor may help make this clearer (or some indication of connection). This makes the relationship between autocrine/paracrine signalling more obvious.
8. Page 7 refers to 683 Prok2 an 887 ProkR2 cells from the scRNAseq data, but this is shown as 683 and 886 in Fig 3D.

Referee #2:

The central circadian clock is localized in the hypothalamic suprachiasmatic nucleus (SCN). Recent work used single cell RNASeq to identify 5 SCN neuronal subtypes in tissue from adult SCN including cluster-specific markers: VIP, AVP, GRP, CCK, and the cell-adhesion regulator C1ql3 (Wen et al., 2020).

This study followed a similar approach to conduct single-cell sequencing of SCN organotypic slices. They found also a number of neuropeptidergic signalling axes between these nodes, and built neuropeptide-specific networks. The authors focused on the Prokineticin2 (Prok2)/ Prokineticin receptor2 (ProkR2) signalling axis.

The authors went on to examine the contribution of this signalling pathway and found evidence that the Prok2-ProkR2 neuropeptides are a key regulator of SCN period and rhythmicity. The authors contrast the role of Prok2-ProkR2 as an integrator compared to VIP as a distributor of circadian information.

Overall, this beautifully written study was a pleasure to read. Initially, I was concerned that there may be too much overlap with the scRNA-seq from the Yan laboratory. However, as soon as I got into this ms, I was not longer concerned. By using the slice, the authors could claim circadian vs. diurnal rhythm but really the overlap was limited.
The present study focused on the Prokineticin2 (Prok2)/ Prokineticin receptor2 (ProkR2) signalling axis which has really been neglected compared to VIP and AVP networks.

The figures are very nice and help the reader follow the work.

The data supports the conclusions.

I just had a few minor concerns.

First, as a field, we likely do not give enough credit to the work of Dr. Zhou and his team at UCI. The discussion should include more description of their work.
• Attenuated circadian rhythms in mice lacking the prokineticin 2 gene. Li JD, Hu WP, Boehmer L, Cheng MY, Lee AG, Jilek A, Siegel JM, Zhou QY. J Neurosci. 2006 Nov 8;26(45):11615-23. doi: 10.1523/JNEUROSCI.3679-06.2006.
• Regulation of prokineticin 2 expression by light and the circadian clock. Cheng MY, Bittman EL, Hattar S, Zhou QY. BMC Neurosci. 2005 Mar 11;6:17. doi: 10.1186/1471-2202-6-17.
• Altered circadian and homeostatic sleep regulation in prokineticin 2-deficient mice. Hu WP, Li JD, Zhang C, Boehmer L, Siegel JM, Zhou QY. Sleep. 2007 Mar;30(3):247-56.

• Overexpression of Prokineticin 2 in Transgenic Mice Leads to Reduced Circadian Behavioral Rhythmicity and Altered Molecular Rhythms in the Suprachiasmatic Clock. Li X, Zhang C, Zhou QY. J Circadian Rhythms. 2018 Nov 6;16:13. doi: 10.5334/jcr.170.

Second, I think that the figure legends need some attention. This may be a stylistic difference but I like to be able to read the legends and know what is going on. I felt like they were in short-hand and would be fine for someone working in the area but not for more general readers.

I cannot speak to the ScRNASeq analysis, but from a circadian perspective, this is first-rate work.

Referee #3:

The manuscript of Morris et al. entitled "Single-cell transcriptomics of suprachiasmatic nuclei reveal a Prokineticin-driven circadian network" reports a single-cell RNAseq (scRNAseq) study of SCN brain slices. Together with real-time imaging, the authors propose Prok2-ProkR2 axis as a key regulator of SCN period and rhythmicity. SCN neuropeptide-receptor signaling network is key to our understanding of the mechanism of circadian pacemaking activity of SCN. The authors aim to construct such network by scRNAseq and validate it by imaging analysis. However, there is a major experimental limitation of using organotypic SCN brain slices for scRNAseq and collecting only two circadian time points (CT7.5 and CT15.5). In addition, all experiments described here are conducted on brain slices and there is a lack of novelty in the main finding derived from this data, as the role of Prok2 and Prokr2 in circadian rhythm has been reported earlier by this group (Prosser et al., 2007) and other group (Cheng et al., 2002; Li et al., 2006) using transgenic animals.

Major Points:

1. The choice of "free-running organotypic SCN slices" is inappropriate for SCN scRNAseq study. Three days of brain slice in culture can result in the degradation of RNAs leading to the artifact in RNAseq data. It is also unclear why the authors chose the two time points: CT7.5 and CT15.5 (at 8-hour interval) in the SCN sampling?

2. The SCN cell clusters found in this study lack independent validation such as in situ hybridization. So it is hard to know whether they are really subtypes present within SCN or outside of SCN.

3. In Fig. 3-4, the authors defined different SCN cell groups between day and night. This raised the question whether the cell groups defined in this paper reflects cell states rather than cell types/subtypes that should be invariable between day and night.

4. The analysis of "topology of SCN neuropeptidergic signalling axes" appears rather superficial. It is hard to see how "Prok2-Prokr2" axis was singled out for downstream analysis. This leads to a general disconnect between Fig. 1-4 and Fig. 5-9. The day-night difference in Prok2-Prokr2 signaling can be simply explained by the circadian expression of Prok2, which is also known.

5. In the imaging experiments in Fig. 5-9, although the phase difference of Prok2+ vs. Prokr2+ cells is similar to that of Vip+ vs. Vipr2+ cells, the effects of PROK2-PROKR2 on SCN oscillation is apparently weaker than VIP-VPAC2 axis. This again raised the question why Prok2-Prokr2 axis is important. These imaging results on brain slices run against the earlier animal studies on Prok2 and Prokr2, in which they have strong effects on circadian behaviors (Cheng et al., 2002; Li et al., 2006;

Prosser et al., 2007).

Minor points:

It was incorrectly referred in Page 3 line 29. Park et al. characterized the transcriptional, spatial, and functional organization of 352 SCN neurons by single-cell PCR, not by scRNAseq.

Cheng, M.Y., Bullock, C.M., Li, C., Lee, A.G., Bermak, J.C., Belluzzi, J., Weaver, D.R., Leslie, F.M., and Zhou, Q.Y. (2002). Prokineticin 2 transmits the behavioural circadian rhythm of the suprachiasmatic nucleus. Nature 417, 405-410.
Li, J.D., Hu, W.P., Boehmer, L., Cheng, M.Y., Lee, A.G., Jilek, A., Siegel, J.M., and Zhou, Q.Y. (2006). Attenuated circadian rhythms in mice lacking the prokineticin 2 gene. J Neurosci 26, 11615-11623.
Prosser, H.M., Bradley, A., Chesham, J.E., Ebling, F.J., Hastings, M.H., and Maywood, E.S. (2007). Prokineticin receptor 2 (Prokr2) is essential for the regulation of circadian behavior by the suprachiasmatic nuclei. Proc Natl Acad Sci U S A 104, 648-653.

Karin Dumstrei, PhD
Senior Editor
The EMBO Journal

Dear Dr. Dumstrei,

Thank you for your email, the précis of the Referees' comments and the associated guidance. My co-Authors and I are very pleased to receive the three constructive critiques of our work. Below, we detail our responses to the Referees, in green text and the revised manuscript text is highlighted.

Referee #1:

Mammalian circadian rhythms ……………………….… work provides a very valuable extension of our understanding of the role of specific neuronal populations and signalling within the SCN. Specific comments are provided below.

Main comments

1. Methodological justification. Why were the specific timepoints chosen (CT7.5 and 15.5)? This is not clearly explained in the manuscript.

The timepoints of CT7.5 and CT15.5 were chosen to reflect the circadian cycle of neuronal activity in SCN organotypic slices, as revealed by cytosolic $[Ca^{2+}]_i$ levels reported by synapsin-driven GCaMP. These peak between CT7.00 and CT8.00, as published in our most recent work related to the current manuscript (Patton et al., Nat Comms 2020) which showed that SCN neuronal $[Ca^{2+}]_i$ levels have a sharply defined peak at CT7.6±0.2h. Equally, in the same study we showed that the neurons enter their broader interval of quiescence from CT15.00-CT16.00. We believe that these two timepoints encompass the most informative phases regarding the likely changes of the transcriptomic state of SCN cells and their related functions. This has been made explicit in the revised text and we show how these phases were identified in new Appendix Figures S1A, B:

"To obtain a comprehensive transcriptomic dataset of mouse SCN cells, free-running organotypic SCN slices were collected in subjective day, during peak neuronal activity levels (Patton et al., 2020), at circadian time (CT)7.5. This phase was identified by prior recording of Per2::Luciferase bioluminescence rhythms (**Appendix Fig. S1A**)."

"SCN time-keeping is under-pinned by its spontaneously changing transcriptional state across circadian time. We therefore also determined the transcriptome of SCN slices in circadian night, at CT15.5 (**Appendix Fig. S1B**), when the activity of SCN neurons enters its quiescent interval (Patton et al., 2020)."

2. Expression data for neuronal subtypes. In figure 2B, day vs night enriched genes are shown. I may have missed this, but is a breakdown of these genes by the identified neuronal subtypes included?

Alas, we did not provide such a potentially interesting breakdown (not least because of space limitations). Please see comment below re. data availability.

Are day/night differences selectively enriched in specific cell types?

They very possibly are, but again this was not analysed by us. We thank the Referee for highlighting the potential of specific cell-types particularly contributing to the differing transcriptional states at day and night in the SCN, and again please see comment below re. data availability. Certainly, Prok2 stands out in this regard. Prompted by the Referee's query, we have included Gene Ontology analysis to characterise further the major changes in transcriptional state between day and night. The text is amended:

……… up-regulated in the daytime. Overall, gene ontology identified highly significant up-regulation of genes associated with synaptic function (including GABA and dopamine) and cell contacts in circadian day, whilst circadian night was characterised by protein degradation and loss of neuropeptide transport (**Appendix Fig. S2**). The most significantly ……..

Relating to this, the inclusion of the raw data for each figure is very helpful. However, are full transcriptomic data (all expressed genes) available for each of the specific neuronal subpopulations? This would an incredibly ………significant additional added value and be a very useful resource. For example, if gene X is identified as being of interest in a whole SCN study, is its expression confined to a specific neuronal subtype within the SCN?

Yes - we have deposited all of our data with the GEO database, and on publication they will be freely available (accession number: GSE167927). The proposed analysis, i.e., asking if a particular gene X is present/ up-regulated in one of the 11 sub-populations outlined in this study, will be readily and easily achieved by any interested researcher. We would be delighted for other groups to perform such analyses on our dataset, as their interests dictate. This is now explicit in the data availability statement in the text.

ScRNASeq data: NCBI Gene Expression Omnibus with the accession number: GSE167927.

3. Behavioural predictions. Conducting in vivo behavioural evaluation of the Prok2/ProkR2 axis would be challenging and would presumably take a whole new paper to investigate.

We thank the Referee for prompting us to place our findings of intra-SCN Prok2 signalling within the wider context of behavioural activity, whilst recognising that in vivo work would be too challenging for now

However, it would be helpful to extend the discussion to relate the observed circadian and sleep phenotypes of Prok2/ProkR2 mice (e.g. Li et al., 2007; Prosser et al., 2007; Hu et al., 2007) to the new insights provided by this study. For example, the precision of timing, the redistribution of activity, altered body temperature, glucocorticoid rhythms and sleep.

Thank you for the suggestion. We have added the following to the Discussion:

The most pronounced circadian change of network configuration was seen for the Prok2/ProkR2 axis. *Prok2* has four E-box elements that drive its circadian transcription (Cheng et al., 2002, Masumoto et al., 2006) and recombinant Prok2 can induce locomotor activity in rats (Cheng et al., 2002), whilst genomic ablation of Prok2 in mice attenuates clock-controlled physiology, including glucocorticoid signalling and body temperature rhythms (Li et al., 2006, Li et al., 2018). Moreover ProkR2$^{-/-}$ mice lose precise timing of nocturnal locomotor activity onset (Jethwa et al., 2008, Prosser et al., 2007) and Prok2$^{-/-}$ mice show altered circadian and homeostatic regulation of sleep and disrupted post-deprivation recovery sleep (Hu et al., 2007). Given that SCN TTFL rhythms of ProkR2$^{-/-}$ mice appeared normal, these findings placed Prok2-signalling downstream of the SCN clock, as a key output, and argued against any intrinsic network role. Our identification of the Prok2/ProkR2 axis as a pacemaker within the SCN now raises the possibility that at least some of these consequences of global mutation arise from loss of SCN precision. Indeed, Prok2 can suppress GABAergic function in SCN slices in circadian daytime by reducing the amplitude of mini-inhibitory post-synaptic currents, disinhibiting the firing rate of SCN cells (Ren et al., 2011).

Also:

Given that Prok2R is expressed widely in areas innervated by SCN Prok2^{+} cells (e.g., PVN, medial thalamus and DMN), the newly developed AAVs offer the means for more refined analyses to parse out local and distal effects of Prok2-signalling in both the SCN and its targets. For example, can conditional activation or silencing of these pathways initiate, suppress or otherwise re-programme circadian behaviour?

What would be the prediction regarding conditional knockout of Prok2/ProkR2 in the SCN?

We have added the following to the Discussion:

consequent attenuation of activity-induced Per2 (Colwell, 2011). Conditional knockout of Prok2/ProkR2 in the SCN would therefore be expected to enhance GABAergic tone, possibly reducing neuronal firing rate and attenuating Prok2- dependent and -independent output signals, weakening circadian co-ordination.

And would the loss of Prok2/ProkR2 be expected to result in an SCN that is more prone to environmental disruption?

We have added the following to the Discussion running on from the above edit:

…………weakening circadian co-ordination and making the circadian system more prone to environmental disruption.

Do the current results provide any insight into why entrainment to light may be unaffected (e.g. could light input primarily involved different neuronal subtypes?).

We have added the following to the Discussion:

………. re-programme circadian behaviour? Importantly, our scRNASeq data show that the Prok2/ProkR2 axis is distinct from the retinorecipient VIP/GRP cells (only 6% of *Vip* and 3% of *Grp*$^+$ SCN neurons co-express *Prok2*) and this may explain why Prok2-signalling is not involved in phase-entrainment of the SCN. Its transcriptional regulation by retinal input (Cheng et al., 2005) is likely mediated indirectly via VIP-mediated signalling (Hamnett et al. 2019). This provides a clear distinction between the Prok2/ProkR2 and VIP/VPAC2 axes (Hamnett et al., 2019, Patton et al., 2020).

Finally, we make an explicit comparison between Prok2- and VIP-signalling:

…….. "driven" circuit: Prok2-signalling mediating robustness and tight coupling as an "integrator" hub, whilst the VIP-mediated distributor hub determines cellular and ensemble phases.

In hindsight, one advantage of this study was that samples were taken from SCN slices, so the patterns of gene expression observed could not be affected by feedback from behavioural state.

Thank you for this recognition. We agree, and this point is a pertinent complement to a comment made by Referee 3 about the potential limitations of the organotypic slice. Inevitably, all experimental approaches have merits and limitations. In our current work, we wished to examine the intrinsic organisation and properties of the SCN network, for which the isolated slice is ideally placed.

Minor comments

1. It may be a journal requirement, but the very short figure legends did not help with a detailed comprehension of data presented in the figures. More information in these figure legends would make this much more accessible.

Yes - the legends have been amended accordingly. They are detailed at the end of this document and highlighted in the revised text.

2. Presumably the whole trimmed SCN slices were used for the scRNAseq? Presumably the surrounding tissue accounts for the 'extra-SCN hypothalamic neurons'

Yes, that is the case. A perfect dissection of the living SCN tissue is not possible because of its three-dimensional geometry.

3. I was unclear how single cell dispersions were made from circa 17 SCN slices (page 4).

In each sequencing run the single cells of ca. 17 SCN slices were loaded into the 10X Genomics Chromium Chip. To achieve this, firstly the SCN slices were pooled into one Falcon tube and this sample then underwent single-cell dissociation using papain enzymatic digestion (Worthington Dissociation Kit).

We have amended the associated methods passage:

….. enzymatic digestion of pooled organotypic SCN slices …….were maintained throughout the manufacturer's protocol. Each of 16-20 SCN slices that were pooled per sequencing run into a single sample were first imaged on …… healthy sample tissue. The slices were gently removed from their membrane supports, pooled into one Falcon tube and incubated in ……

We have amended the associated figure legend:

Figure 1. ScRNASeq of mouse SCN slices at two opposing circadian time-points

A. Schematic work-flow for 10X Genomics scRNASeq of pooled SCN slices after three days in culture. For each sequencing run ca. 17 organotypic SCN slices were pooled into a single sample and their cells dissociated using papain to obtain single cell suspension of ~8,000 cells/µL. Dispersed single cells, along with barcoded 10X Genomics Chromium Single Cell 3' v2 technology gel beads, were partitioned into water-in-oil droplets. Within these droplets, reverse transcription and amplification steps generated cDNA libraries for the barcoded single cells.

4. In Figure 2 is the criteria of $p<0.05$ based on FDR corrected p-values?

Yes. $P<0.05$ is based on p-values that are adjusted for multiple testing using the Benjamin-Hochberg procedure to control FDR. This is now added to the text:

We next asked which were the main circadian day-/night-defining genes by using a negative binomial exact test (CellRanger in-built algorithm, multiple testing corrected using Benjamin-Hochberg) (**Fig. 2B**).

The corresponding figure legend has also been amended:

Figure 2. Transcriptional state of SCN neurons at day and night

B. Volcano plot showing the magnitude ……….. negative binomial exact test (red dots, dashed line *p*<0.05 adjusted for multiple testing using Benjamin-Hochberg).

5. In Figure 3, the 8 neuronal clusters are classified using several neuropeptidergic identifiers. I was unclear why these specific genes were used as identifiers? Presumably other marker genes from each cluster could have been used? Were these chosen due to previously characterised SCN expression? Or were they the most unique identifiers of each cluster (and if so, based on what metric)? Whilst this is not a limitation in any way, the rationale for classification here could be made clearer.

All of the marker genes shown are within the top 12 most up-regulated genes per cluster, as defined by the negative binomial exact test. For brevity and clarity, from the 12 genes distinguishing each group, three genes associated with tangible network-regulatory properties (neuropeptides, receptors etc.) were chosen as “labels”. As noted above, on publication the dataset will be openly available to interested parties who will be able to re-examine and refine/ enhance the clustering to match their particular interests.

We have amended the text thus:

To identify SCN neuronal sub-populations, we used unsupervised graph-based clustering of the transcriptomes of the 7,931 daytime SCN neurons. This identified eight clusters for which three marker genes, chosen from the top 12 most up-regulated genes per cluster, were used as cluster labels: Groups A (*Gem*$^+$/*Ppp1r17*$^+$/*Anxa5*$^+$), B (*Vip*$^+$/*C1ql3*$^+$/*Calb2*$^{+)}$, C (*Grp*$^+$/*Synpr*$^+$/*Cd24a*$^+$), D (*Prok2*$^+$/*Avp*$^+$/*Nms*$^+$), E (*Cck*$^+$/*ProkR2*$^+$/*Nsmf*$^+$), F (*Mef2c*$^+$/*Peg10*$^+$/*Pcdh11x*$^+$), G (*Gadd45a*$^+$/*Hspa1a*$^+$/*Cck*$^+$) and H (*Vipr2*$^+$/*Lbh*$^+$/*Gas7*$^+$) (**Fig. 3A**). For all three sequencing runs the algorithm consistently found the same marker genes defining the same eight groups.

6. More information regarding the meaning and interpretation of the signalling topology maps shown in Figure 4 would be helpful.

Yes, we have added the text thus:

…………. The strength of intra-SCN communication used by each neuropeptide ligand-receptor pair was inferred from the expression level of each gene of interest in each cluster. More specifically, the average cellular count of the neuropeptide gene expression across one cluster was multiplied with the average cellular count of the corresponding neuropeptide receptor gene expression in another cluster Bentley et al., 2016, Zingg et al., 2014). For example, the strength of …………. at circadian day (**Fig. 4A**, see **Fig. EV1F,G** for the unscaled maps) formalises the Venn diagram (**Fig. 3D**). The overall degree of connectivity within each inferred signalling network is further quantified as the sum of the edge (connection) weights (**Fig. 4B**, sigma values).

The corresponding figure legend has also been amended:

Figure 4. Topology of transcriptionally inferred SCN signalling axes
Schematic of inferred topology of neuropeptidergic signalling axes between SCN neuronal sub-populations in circadian day. Top: clusters are annotated in the overview t-SNE for circadian day (**A.**) and night (**B.**), respectively. Below: the identified SCN subpopulations are depicted as nodes of each neuropeptide-receptor network, connected by edges that are weighted (shown as differential line thickness) based on the average count of the respective cellular gene expression for each ligand and receptor. The nodes of the inferred topology networks are colour-coded to match those of the overview t-SNE plots. Nodes further highlighted in yellow mark ligand-enriched source clusters. The edges are colour-coded for each neuropeptide-receptor signalling pair: *Prok2/ProkR2* – purple, *Vip/Vipr2* – green, *Avp/Avpr1a* – blue. The sum of edge weights within each day and night inferred signalling network is presented below each map to provide a quantification of the overall degree of connectivity.

7. I found the classification of subtypes by neuropeptides and their receptors very informative. Visualisation with related colours for each neuropeptide and receptor may help make this clearer (or some indication of connection). This makes the relationship between autocrine/paracrine signalling more obvious.

We thank the Referee for the very helpful suggestion. We have amended Figure 4 accordingly. Also, please see previous response regarding the amended figure legend.

8. Page 7 refers to 683 Prok2 an 887 ProkR2 cells from the scRNAseq data, but this is shown as 683 and 886 in Fig 3D.

This is now corrected in the text thus:

……….accord with the daytime scRNASeq data, i.e., 77% (683/886) Prok2.

Referee #2:

The central circadian……………The data supports the conclusions.
I just had a few minor concerns.

First, as a field, we likely do not give enough credit to the work of Dr. Zhou *and his team at UCI. The discussion should include more description of their work.*
• Attenuated circadian rhythms in mice lacking the prokineticin 2 gene. Li JD, Hu WP, Boehmer L, Cheng MY, Lee AG, Jilek A, Siegel JM, Zhou QY. J Neurosci. 2006 Nov 8;26(45):11615-23. doi: 10.1523/JNEUROSCI.3679-06.2006.
• Regulation of prokineticin 2 expression by light and the circadian clock. Cheng MY, Bittman EL, Hattar S, Zhou QY. BMC Neurosci. 2005 Mar 11;6:17. doi: 10.1186/1471-2202-6-17.

• Altered circadian and homeostatic sleep regulation in prokineticin 2-deficient mice. Hu WP, Li JD, Zhang C, Boehmer L, Siegel JM, Zhou QY. Sleep. 2007 Mar;30(3):247-56.
• Overexpression of Prokineticin 2 in Transgenic Mice Leads to Reduced Circadian Behavioral Rhythmicity and Altered Molecular Rhythms in the Suprachiasmatic Clock. Li X, Zhang C, Zhou QY. J Circadian Rhythms. 2018 Nov 6;16:13. doi: 10.5334/jcr.170.

We thank the Referee for ensuring we do not overlook this body of work. Accordingly, we have amended the text and all of the citations are now included:

………..its circadian transcription (Cheng, Bullock et al., 2002) (Cheng, Bittman et al., 2005) (Masumoto, Nagano et al., 2006) and recombinant Prok2 can induce locomotor activity in rats (Cheng et al., 2002), whilst genomic ablation of Prok2 in mice attenuates clock-controlled behaviour and physiology, including glucocorticoid signalling and body temperature rhythms, (Li, Hu et al., 2006) and mis-expression of Prok2 disrupts behavioural rhythms (Li, Zhang et al., 2018). Moreover ProkR2$^{-/-}$ mice lose precise timing of nocturnal locomotor activity onset (Jethwa, I'Anson et al., 2008, Prosser, Bradley et al., 2007) and Prok2$^{-/-}$ mice show altered circadian and homeostatic regulation of sleep and disrupted post-deprivation recovery sleep (Hu, Li et al., 2007). Given that………

And:

….. Its transcriptional regulation by retinal input (Cheng et al., 2005) is likely mediated….

Second, I think that the figure legends need some attention. This may be a stylistic difference but I like to be able to read the legends and know what is going on. I felt like they were in short-hand and would be fine for someone working in the area but not for more general readers.

We thank the Referee. This point echoes that from Referee 1 and we have addressed it as suggested.

Referee #3:

The manuscript of Morris et al. entitled "Single-cell transcriptomics of suprachiasmatic nuclei reveal a Prokineticin-driven circadian network" reports a single-cell RNAseq (scRNAseq) study of SCN brain slices. Together with real-time imaging, the authors propose Prok2-ProkR2 axis as a key regulator of SCN period and rhythmicity. SCN neuropeptide-receptor signaling network is key to our understanding of the mechanism of circadian pacemaking activity of SCN. The authors aim to construct such network by scRNAseq and validate it by imaging analysis. However, there is a major experimental limitation of using organotypic SCN brain slices for scRNAseq and collecting only two circadian time points (CT7.5 and CT15.5). In addition, all experiments described here are conducted on brain slices and there is a lack of novelty in the main finding derived from this data, as the role of Prok2 and Prokr2 in circadian rhythm has been reported earlier by this group (Prosser et al., 2007) and other group (Cheng et al., 2002; Li et al., 2006) using transgenic animals.

Major Points:

1. The choice of "free-running organotypic SCN slices" is inappropriate for SCN scRNAseq study. Three days of brain slice in culture can result in the degradation of RNAs leading to the artifact in RNAseq data.

We agree with the Referee on the general point that brain slices in culture can potentially undergo RNA degradation. For several reasons iterated below, we do not think that this is the case for the SCN slice.

First, and in contrast to many other brain regions isolated *ex vivo*, it is the case that the SCN organotypic slice retains it functional circadian and metabolic competence. This is evident from our recordings of circadian gene expression using bioluminescent reporters and from intracellular calcium recordings made by ourselves and others and published elsewhere (Patton et al. 2020). These are extremely precise from the first day in culture and remain stable for weeks, subject to supply of luciferin substrate (which is progressively utilised) and fresh medium. To emphasise this stability and precision, which would be compromised by any cellular dysfunction, we have included in Appendix Figure 1A,B the bioluminescent recordings from the "sister" traces to those SCN slices that were collected in circadian day or circadian night for scRNAseq.

Second, our RNAseq analyses were conducted with strict quality control measures, as performed by the central sequencing facility at the Cancer Research UK Institute, Cambridge. These are now added to the text.

scRNASeq libraries were prepared using the Chromium Single Cell Controller (10x Genomics, Pleasanton, CA). Both biochemical and bioinformatic quality control measures were performed to ensure that the average size and cDNA concentration satisfied quality thresholds and that the spread of the sequenced reads across genes was even across single cells (i.e., a normal distribution of genes with at least one UMI tag). Cells were treated following the manufacturer's protocols using 10x Genomics ChromiumTM Single Cell 3' Library and Gel Bead Kit v2 (120267) and ChromiumTM Single Cell A Chip Kit (1000009).

We are happy to provide further specific detail in this response. First, from a biochemical perspective we have added:

A qualitative analysis of the amplified cDNA was run on the Agilent TapeStation High Sensitivity D1000 ScreenTape to determine the average size and cDNA concentration.

This ensured that the concentration, integrity and molecular weight of DNA used satisfied quality measure thresholds. An example of such output, showing the quality of cDNA from a representative sequencing run is shown in the graph below. Poor quality RNA would not generate such high-quality DNA.

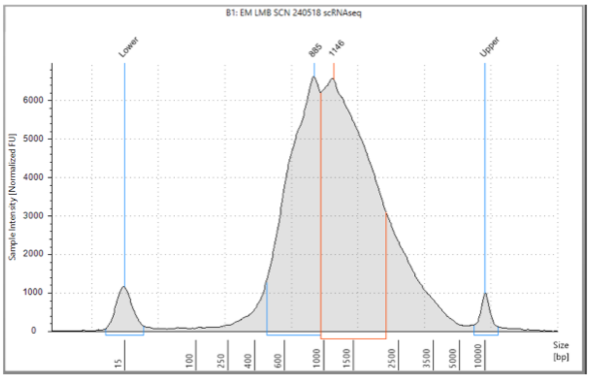

Second, from a bioinformatic perspective a normal distribution of genes with at least one UMI tag, i.e., transcript, was seen across single cells, which is indicative of an even spread of the sequenced reads across genes. Amended text:

……..To ensure high quality data were taken for further analysis, only genes expressed in three or more cells, and cells with at least 100 detected genes, were retained in the dataset. A verification step in Seurat was performed to ensure all mitochondrial transcript-expressing cells were removed from the dataset during Cell-ranger processing.

The above is a common quality control metric, as lysis of a single cell results in the release of cytoplasmic RNA, whereas that enclosed in mitochondria is retained and sequenced. Therefore, the percentage of mitochondrial genes per cell was plotted and lower quality cells were omitted from the data set.

A gene-plot was created to assess the relationship between the number of unique molecules and the number of genes captured. This highlighted that more unique molecules were captured when more genes were probed. Log normalization of the dataset was performed to enable cells to be compared with each other. For this, the gene expression measurements for each cell were normalised by its total expression, scaled by 10,000 and log-transformed. These quality control measures are shown below:

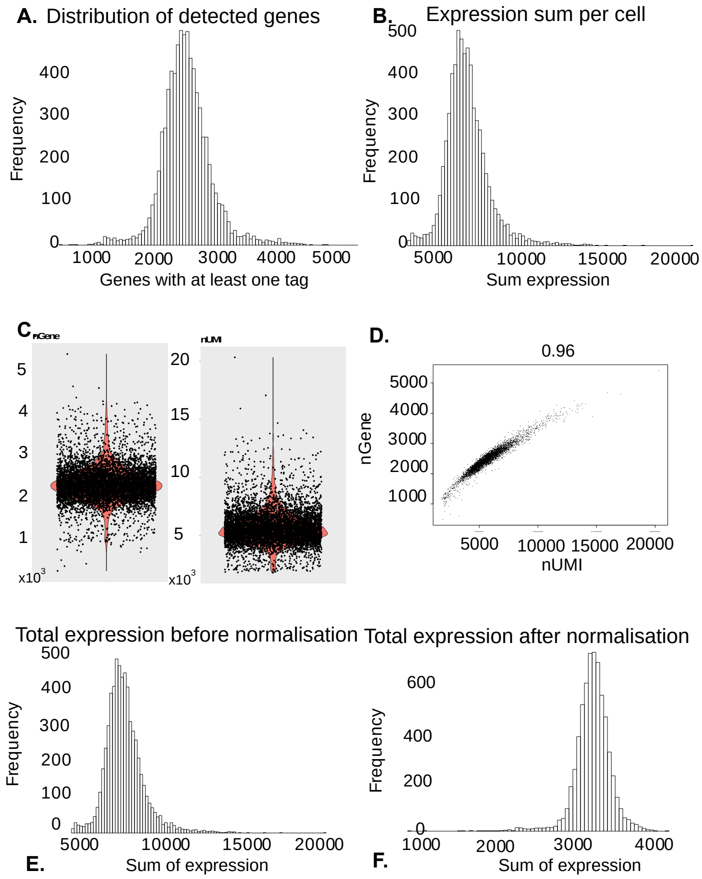

Quality control metrics of single-cell RNA seq. data. **A**. Normal distribution of genes with at least one UMI tag was obtained indicative of an even spread of the sequenced reads across genes. **B**. The average expression level and the sum of genes detected in a single cell were determined. **C. & D**. The relationship between the number of unique molecules and the number of genes captured. **E.** Gene expression measurements were **F.** normalized, scaled and log-transformed

It is also unclear why the authors chose the two time points: CT7.5 and CT15.5 (at 8-hour interval) in the SCN sampling?

Please see our response to Referee #1 on this matter.

2. The SCN cell clusters found in this study lack independent validation such as in situ hybridization. So it is hard to know whether they are really subtypes present within SCN or outside of SCN.

We do show confirmatory in situ hybridisation images for representative SCN cell types, and in addition many of the genes we consider are shown by the Allen Brain Atlas (and the wider literature) to be expressed in the SCN. Moreover, although our analysis of cell groups is more extensive than those by Wen et al. and Park et al., where our three studies do overlap, we have direct correspondence, which adds to confidence in our analytical approach.

Finally, the analysis was conducted only on characteristic SCN neurons, extra-SCN hypothalamic cell types (as well as numerous other cell types) being excluded.

3. In Fig. 3-4, the authors defined different SCN cell groups between day and night. This raised the question whether the cell groups defined in this paper reflects cell states rather than cell types/subtypes that should be invariable between day and night.

We agree with the Referee that this is a fair distinction to make: cell-type versus state. Within a single time-point (day or night), each cluster is a group of cells with a common transcriptional state that distinguishes them from other clusters, each of which is a different group of cells with its own defining transcriptional state. The nuance arises, however, when comparing between time-points, insofar as the cells are still present: there is no neurogenesis and they do not stop existing. Nevertheless, their transcriptional state may have altered. For daytime groups A, B, C, D & E there were obvious transcriptional parallels to the corresponding night-time groups, and their relative abundances did not change, so this issue is less pressing. On the other hand, groups F, G and H had no simple transcriptional match in the night-time and so new "groups" but in reality, new "cellular states" were listed: I, J and K. Parsimony would suggest that cells in groups F, G and H altered their transcriptional profiles to adopt these new states, although in the absence of repeated measures from the same individual cells through time (a technical impossibility, of course) their correspondence is not knowable. We have therefore added the following explanatory note to clarify the matter:

……………… neuropeptidergic identity based on ligand and/or receptor expression was a commonly distinguishing feature across the sequenced neuronal cell types, and was accompanied by distinct, cluster-defined transcriptional programmes (or states). Similarly, clustering of the 9,432 circadian night SCN neurons identified eight distinct sub-populations. Three of these showed strong similarity to daytime Groups A, B and C, whilst two showed partial similarity with CT7.5 Groups D and E, and likely represent the same subpopulations. Nevertheless, three clusters had transcriptional profiles not evident in circadian day and so were named Groups I, J and K. In the absence of neurogenesis, we interpret these as distinct, newly adopted states of the cells identified as Groups F, G, H in day-time samples.

4. The analysis of "topology of SCN neuropeptidergic signalling axes" appears rather superficial. It is hard to see how "Prok2-Prokr2" axis was singled out for downstream analysis.

By the nature of scRNAseq, we had no *a priori* expectations of what types of network pattern would be revealed. Clearly, neuropeptidergic signalling "fell out" of the dataset as a defining feature of cell groups/ states. Moreover, Prok2 was the most emphatic day-time up-regulated gene. Other genes in this category included very well known clock-driven transcription factors (Nr1d1, Dbp) and signalling factors (Rgs16, Dusp4) on which we and others have worked extensively. Prok2 stood out as an opportunity to discover something new about intra-SCN time-keeping: the unbiased analysis of putative signalling networks showed it to change dramatically between circadian day and night, even more so than the

familiar VIP and AVP axes. Our data therefore presented Prok2-signalling within the SCN as an exciting, novel target. To pursue this, we made significant efforts to generate the AAV vectors in order to explore cell functions, adding further novelty to the work.

This leads to a general disconnect between Fig. 1-4 and Fig. 5-9.

With respect, we do not accept the idea of a "general disconnect". For all of the merits of their work, Wen et al. and Park et al. did not engage in any functional testing of their identified SCN cell clusters. In our work, we conducted an extensive categorisation of SCN cellular transcriptomes, which highlighted the Prok2/ProkR2 clusters as a dramatically circadian axis, and one that is distinct from those regularly studied i.e., AVP, VIP. Given that the role of Prok2/ ProkR2 cells within the SCN has been overlooked by many groups, including us, we then went on to explore their contribution in the SCN network. There is obviously a change in methodological approaches, but we see that as a positive evolution of our work as we progressed from description to interrogation.

The day-night difference in Prok2-Prokr2 signaling can be simply explained by the circadian expression of Prok2, which is also known.

Yes, we agree that circadian expression of Prok2 is known. What we do here is to put that change into its cellular context and the context of the network, in which circadian patterns of VIP- and AVP-dependent signalling are also described. To reiterate our earlier comments, following the transcriptomic leads has allowed us to identify the Prok2 and Prok2R axis as a novel and distinct element of the SCN network, with a topology that does not map onto the VIP or AVP topologies.

5. In the imaging experiments in Fig. 5-9, although the phase difference of Prok2+ vs. Prokr2+ cells is similar to that of Vip+ vs. Vipr2+ cells, the effects of PROK2-PROKR2 on SCN oscillation is apparently weaker than VIP-VPAC2 axis.

We would not say weaker, but different. The phase-shifting effects of Prok2 are clearly weaker than those of VIP (i.e., they do not exist), but of course that distinction is one part of the point we seek to make (please see our response to Referee 1 regarding entrainment, for example). We interpret the effects of Prok2 on TTFL amplitude and baseline via an interaction with GABAergic tone across the SCN, and this is something we shall pursue in follow-up work. The pronounced effects on network period and oscillation of the Prok2/ProkR2 cellular axis raise the question of the mediating signalling cascade: again, a topic for future analyses.

These imaging results on brain slices run against the earlier animal studies on Prok2 and Prokr2, in which they have strong effects on circadian behaviors (Cheng et al., 2002; Li et al., 2006; Prosser et al., 2007).

We agree with the Referee that we and others have shown that, in the whole-mouse mutants, the loss of Prok2-signalling does indeed have strong effects on circadian behaviour and other outputs, and we highlight this in our text (including our responses to Referees 1 and 2). The results from our isolated SCN brain slice studies now show a powerful effect of the Prok2/ ProkR2 cellular axis on period and oscillatory competence. The next stages of our work will be to explore how these newly validated (AAV-based) manipulations in the SCN ex vivo translate into behavioural and physiological rhythms in vivo. What actions are intra-SCN specific and what effects translate solely to the distal control of output rhythms – i.e., are the effects of these cells exerted within the SCN and/ or within its target sites? These points are also made by Referee 1 and have been incorporated into our modified text (please see above).

Minor points:

It was incorrectly referred in Page 3 line 29. Park et al. characterized the transcriptional, spatial, and functional organization of 352 SCN neurons by single-cell PCR, not by scRNAseq.

Thank you, we have corrected the text accordingly:

Transcriptomic analysis using singe-cell PCR on 87 candidate genes recently provided greater resolution, highlighting distinct cellular sub-populations within the neuropeptidergic topology of the SCN (Park et al., 2016).

Amended Figure Legends (Referees 1 and 2)

Figure 1. ScRNASeq of mouse SCN slices at two opposing circadian time-points

A. Schematic work-flow for 10X Genomics scRNASeq of pooled SCN slices after three days in culture. For each sequencing run ca. 17 organotypic SCN slices were pooled into a single sample and their cells dissociated using papain to obtain single cell suspension of ~8,000 cells/µL. Dispersed single cells, along with barcoded 10X Genomics Chromium Single Cell 3' v2 technology gel beads, were partitioned into water-in-oil droplets. Within these droplets, reverse transcription and amplification steps generated cDNA libraries for the barcoded single cells.

B. t-SNE plot from 13,324 sequenced SCN cells collected at CT7.5, across three independent sequencing runs. Each sequenced cell marked with a unique barcode is represented as a single dot. This dimensionality reduction method aims to maintain both local and global structure of the data by clustering data points of highest similarity nearest to each other.

Cell clusters recognised by the graph-based clustering algorithm are further colour-coded to highlight the cell types: putative SCN neurons, extra-SCN hypothalamic neurons, astrocytes, oligodendrocytes, radial glia, microglia, ependymocytes and endothelial cells.

C. Heatmap of the top 5 up-regulated genes that most distinguish the transcriptional clusters identified in B. The degree of up-regulation is measured as the log 2-fold change ratio of gene expression calculated for cells of each cluster and normalized to a size factor accounting for the total transcript count of each sequenced cell across each cluster.

D. t-SNE plot from 16,996 SCN sequenced cells collected at CT15.5, across two independent sequencing runs. As for B, the cell types: putative SCN neurons, extra-SCN hypothalamic neurons, astrocytes, oligodendrocytes, radial glia, microglia, ependymocytes and endothelial cells, were identified.

E. As C, but for cell clusters identified at CT15.5.

Figure 2. Transcriptional state of SCN neurons at day and night

A. Pooled SCN cells generate a combined dataset of 17,363 cells. The sequenced SCN cells carry explicit barcodes which were used to back-reference sequencing run identity. This yields a clear-cut segregation into two transcriptionally distinct cell groups. Left: 98.6% of cells in the yellow cluster are CT7 5 cells. Right: 98.8% of cells in the grey cluster are CT15.5 cells.

B. Volcano plot showing the magnitude of differential gene up-regulation, as measured by the respective log 2-fold change in expression level, at day (positive) or night (negative) versus statistical significance, as quantified by a negative binomial exact test (red dots, dashed line $p<0.05$ adjusted for multiple testing using Benjamin-Hochberg). Known circadian-regulated genes are labelled.

Figure 3. SCN neuronal sub-population transcriptomes

A. Sub-clustering of eight neuronal populations using unsupervised graph-based clustering within the transcriptomes of CT7.5 (top) and CT15.5 (bottom) SCN cells. The heatmaps show the respective up-regulation of three representative marker genes in each sub-population. When compared, three subpopulations showed strong transcriptional similarity between the time-points of sequencing (Groups A, B and C), two showed partial similarity (Groups D and E) and three were transcriptionally distinct to either CT7.5 or CT15.5 sequenced cells, giving rise to the identification of eleven SCN neuronal sub-populations.

B. Violin plots depicting the distribution of the quantified average cellular expression of *Prok2*, *ProkR2*, *Vip*, *Vipr2*, *Avp*, *Avpr1a, Grp* and *Grpr* in each sub-cluster in CT7.5 (left) and CT15.5 (right) datasets.

C. Visualisation of the location within the pooled t-SNE plot of cells positively expressing >90% of the overall expression range of *Prok2*, *ProkR2*, *Vip*, *Vipr2*, *Avp*, *Avpr1a*, *Grp* and *Grpr*. This shows a contrasting expression pattern of the respective genes in cells collected at CT7.5 versus CT15.5.

D. Venn diagram summarising the degree of non-/overlapping signalling axes: *Prok2* (purple) - *ProkR2* (brown), *Vip* (green) - *Vipr2* (red), *Avp* (blue) - *Avpr1a* (orange) *and Grp* (pink) - *Grpr* (grey). The number SCN neurons collected at CT7.5 positively expressing each gene, which is associated to the respective signalling axis, is further annotated within the population circles.

Figure 4. Topology of transcriptionally inferred SCN signalling axes

Schematic of inferred topology of neuropeptidergic signalling axes between SCN neuronal sub-populations in circadian day. Top: clusters are annotated in the overview t-SNE for circadian day (**A.**) and night (**B.**), respectively. Below: the identified SCN subpopulations are depicted as nodes of each neuropeptide-receptor network, connected by edges that are weighted (shown as differential line thickness) based on the average count of the respective cellular gene expression for each ligand and receptor. The nodes of the inferred topology networks are colour-coded to match those of the overview t-SNE plots. Nodes further highlighted in yellow mark ligand-enriched source clusters. The edges are colour-coded for each neuropeptide-receptor signalling pair: *Prok2/ProkR2* – purple, *Vip/Vipr2* – green, *Avp/Avpr1a* – blue. The sum of edge weights within each day and night inferred signalling network is presented below each map to provide a quantification of the overall degree of connectivity.

Figure 5. Circadian functions of SCN *Prok2*$^{+}$ and *ProkR2*$^{+}$ cells

A. Representative, detrended *Cry1*-luc traces of emitted bioluminescence (photon count values) from 4 SCN slices, pre-transfected with AAV *pCry1*-DIO-Luciferase after addition of (left) *pProk2*.Cre.T2A.mCherry (red, cohort *n*=10) or (right) *pProkR2*.Cre.T2A.Venus (green, cohort *n*=9) transfection.

B. Raster plots (yellow=trough, blue=peak) of *Cry1*-luc bioluminescence oscillations from a representative slice of each cohort. The black arrow marks the time-point of *pProk2*.Cre.T2A.mCherry or *pProkR2*.Cre.T2A.Venus AAV transfection, thus resulting in the evolution of rhythmic *Cry1*-luc bioluminescence.

C. The period of *Cry1*-luc rhythms in SCN slices transfected with either *pProk2*.Cre.T2A.mCherry or *pProkR2*.Cre.T2A.Venus does not differ between treatments (p=0.71, n= 10, 9).

D. Similarly, the amplitude of *Cry1*-luc bioluminescence rhythm from *Prok2*$^{+}$ SCN cells and *ProkR2*$^{+}$ SCN cells, does not differ between (p=0.67).

E. Representative raster plots of single-cell bioluminescence (yellow=minimum, blue=maximum) from Prok2$^{Cry1\text{-}luc}$ (left) or ProkR2$^{Cry1\text{-}luc}$ (right) cells in single slices.

F. Within-slice cellular synchrony depicted in representative Rayleigh plots of the phases of peak *Cry1*-luc bioluminescence for Prok2$^{Cry1\text{-}luc}$ (red) and ProkR2$^{Cry1\text{-}luc}$ (green) cells. The Rayleigh plots can be read as the face of a clock with CT0 marking the top and CT12 the bottom of the plot circle. The black arrow depicts the mean peak phase across the individual oscillators.

G. The mean Rayleigh vector lengths for Prok2$^{Cry1\text{-}Luc}$ (n=5) and ProkR2$^{Cry1\text{-}luc}$ (n=5) (p>0.99) do not differ between the cohorts.

H. Left: confocal images of representative SCN slices dual-transfected with *pProk2*.Cre.T2A.mCherry and *Syn*.GCaMP6f (top), or *pProkR2*.Cre.T2A.Venus and *Syn*.jRCaMP1a (below) (scale bar=500µm). Right, above: representative detrended plots of signal from GCaMP6f (green) traces and *pCry1*-DIO-Luc, emitted from *Prok2*$^{+}$ SCN cells (red). Right, below: representative detrended plots of jRCaMP1a (purple) and *pCry1*-DIO-Luc emitted from *ProkR2*$^{+}$ cells (light green). Orientation bars depict the horizontal axis of the optic chiasm and the vertical axis of the third ventricle for each SCN.

I. After registering the respective peaks of $[Ca^{2+}]_i$ fluorescence to the peaks of Cry1-luc bioluminescence in each respective cohort, phase-alignment between the peaks of Prok2$^{Cry1\text{-}luc}$ and ProkR2$^{Cry1\text{-}luc}$ bioluminescence shows that the intracellular clocks of *Prok2*$^{+}$ cells are phase advanced by ~0.6h to those of *ProkR2*$^{+}$ cells (**p<0.01).

Data information: All values depicted as mean±S.E.M. **C, D, G, I** unpaired two-tailed t-tests.

Figure 6. Exogeneous Prok2 alters oscillation waveform without altering ensemble phase

A. Phase response curve for Prok2- (red) and vehicle-treated (grey) SCN slices (p>0.05). The phase shift (h), calculated from the mean of the first three peaks after Prok2 application in Per2::Luciferase rhythms, is plotted against the CT of treatment. Faded circles (grey=vehicle, red=Prok2) show individual SCN datapoints, whilst the mean values across the vehicle-treated or Prok2-treated cohort within 3 hour time-bins are shown bold.

B. Application of VIP (blue) delivered during CT7-13 served as a positive control and elicited robust phase delays of ongoing Per2::Luciferase SCN slice oscillations when administered between CT9-12.

C. Comparison of phase shifts measured across each treatment (vehicle vs. VIP, *p=0.04) when administered between CT9-12.

D. Representative Per2::Luciferase traces before and after administration (arrow) of vehicle (black), Prok2 (red) or VIP (blue) within CT9-12.

E. Comparison across treatment cohorts of the relative change in baseline of Per2-driven bioluminescence when vehicle, Prok2 or VIP was applied within CT9-12. Only VIP is statistically shown to significantly raise Per2::Luciferase baseline levels (p=0.0007; vehicle vs. Prok2, ns. p=0.17; vehicle vs. VIP, **p=0.002, Prok2 vs. VIP, *p=0.02).

F. The relative change in amplitude of Per2::Luciferase rhythm after treatment with Prok2 or VIP significantly decreases compared to vehicle application (p=0.002; vehicle vs. Prok2, *p=0.01, vehicle vs. VIP, **p=0.001).

G. Relative change in baseline Per2::Luciferase measured from the first cycle after treatment across circadian time, binned in 6 hour intervals (treatment factor p<0.0001). Prok2 within CT12-18 significantly increased baseline Per2::Luciferase more than vehicle, *p=0.01.

H. Relative change in the amplitude of the first Per2::Luciferase cycle measured after treatment across circadian time, binned in 6 hour intervals (treatment factor p<0.0001). Prok2 within CT6-12 and CT18-24 significantly decreased Per2::Luciferase rhythm amplitude compared to vehicle, CT6-12 *p=0.01, CT18-24 *p=0.04.

Data information: All values depicted as mean ±S.E.M. **C, E, F** one-way ANOVA with Tukey's multiple comparisons; **A, G, H, I** two-way ANOVA with Sidak's multiple comparisons. For **G, H** CT0-6 n=7 vehicle and n=8 Prok2 treated, CT6-12 n=10 vehicle and n=24 Prok2 treated, CT12-18 n=8 vehicle and n=9 Prok2 treated, CT18-24 n=3 vehicle and n=10 Prok2 treated.

Figure 7. SCN rhythm amplitude and period are determined by endogenous Prok2 signalling

A. Representative Per2::Luciferase traces before (black) and after vehicle (grey) or Prok2RA (pink) topical application of SCN slices.

B. Comparison of the Per2::Luciferase rhythm amplitude of the cycle preceding and the first and second cycles succeeding vehicle (applied CT23-20 dark grey n=14, CT-20-23 light grey n=6) or Prok2RA (applied CT23-20 purple n=13, CT20-23 pink n=11) treatment (interaction, p<0.0001). A significant decline in amplitude occurred when Prok2RA signalling was blocked between CT20-23 (****p<0.0001).

C. Change in SCN period after treatment with vehicle (grey) or Prok2RA (pink) between CT23-20 and CT20-23 (vehicle CT23-20 vs. Prok2RA CT20-23, *p=0.01; vehicle CT20-23 vs. Prok2RA CT20-23, *p=0.04).

D. Prok2RA application resulted in a significant period lengthening effect of ongoing SCN ensemble Per2::Luciferase rhythms, which was reversed upon drug washout (p=0.0002, ***p=0.0009, *p=0.01).

Data information: All values depicted as mean ±S.E.M. **B** two-way ANOVA with Sidak's multiple comparisons; **C,D** one-way ANOVA with Tukey's multiple comparisons.

Figure 8. *Prok2*$^{+}$ and *Prok2R*$^{+}$ cells control SCN circadian period

A. Representative Per2::Luciferase trace of Cry1$^{-/-}$ SCN slice at baseline (black) and after AAV-mediated (arrows) Cry1 complementation in *Prok2*$^{+}$ cells (red) or *ProkR2*$^{+}$ cells (yellow).

B. Corresponding peak-to-peak period plots for baseline (black), *Cry1*-expressed in *Prok2*$^{+}$ (red), and then in *ProkR2*$^{+}$ cells (yellow). Individual slices are depicted as grey traces, filled points are their mean±SEM.

C. Group data (*n*=5) across three Per2::Luciferase cycles as shown in B. (*p*=0.006; baseline vs. *Prok2*.Cre **p*=0.04, baseline vs. *Prok2*.Cre and *ProkR2*.Cre **p*=0.02, *Prok2*.Cre vs. *Prok2*.Cre and *ProkR2*.Cre ns. *p*=0.95).

D. As in A but with order of transfection reversed; green =Cry1 only in *ProkR2*$^{+}$ cells (baseline vs. *ProkR2*.Cre **p*=0.04).

E, F. As in B and C but with transfection order reversed (green =Cry1 expression in *ProkR2*$^{+}$ cells only, *n*=6) (*p*=0.006; baseline vs. *ProkR2*.Cre **p*=0.03, baseline vs. *ProkR2*.Cre and *Prok2*.Cre ***p*=0.009, *ProkR2*.Cre vs. *ProkR2*.Cre and *Prok2*.Cre ns. *p*=0.1).

G. The final circadian periods of Cry1$^{-/-}$ SCN slice double-transduced in complementary reverse orders did not differ between each other, *p*=0.7.

H. Period lengthening occured at the same rate when *Prok2*.Cre or *ProkR2*.Cre was the first AAV to be applied (*p*=0.1; *Prok2*.Cre - baseline vs. 1 AAV, **p*=0.01; baseline vs. both AAVs, **p*=0.04; *ProkR2*.Cre – baseline vs. 1 AAV, **p*=0.03; baseline vs. both AAVs, ***p*=0.01.

Data information: All values depicted as mean±S.E.M. **C, D, E, F** one-way ANOVA with Tukey's multiple comparisons; **G** unpaired two-tailed t-test; **H** two-way ANOVA with Sidak's multiple comparisons. In histograms individual points are individual slices, bars are their mean±SEM.

Figure 9. *Prok2*$^{+}$ and *Prok2R*$^{+}$ cells can initiate de novo SCN network rhythms

A. Representative Per2::Luciferase traces of Cry1,2$^{-/-}$ SCN slices at baseline (black, arrhythmic), after Cry1 complementation in *Prok2*$^{+}$ cells (red) and further complementation in *ProkR2*$^{+}$ cells (yellow, rhythmic) (cohort *n*=8).

B. as in A but order of complementation reversed (green =Cry1 complementation in *ProkR2*$^{+}$ cells) (cohort *n*=8).

C. SCN period after Cry1 expression in both *Prok2*$^{+}$ and *ProkR2*$^{+}$ cells did not differ with order of transduction (*p*=0.4).

D. Bioluminescence rhythm amplitude did not differ with order of complementation (*p*=0.96).

E. Paired goodness-of-fit values for Per2::Luciferase oscillations of baseline, single-transfection and double-transfection SCN (*p*=0.02, *Prok2*.Cre before and after, ns. *p*=0.17; *ProkR2*.Cre before and after, ns. *p*=0.99; both *Prok2*.Cre and *ProkR2*.Cre applied before and after ****$p<0.0001$). All values depicted as mean±S.E.M.

F. Binary representation of initiated slice elements of a representative SCN slice (CCD recorded bioluminescence pixels) – black indicates non-initiated and pink indicates initiated, i.e., rhythmic elements (cohort *n*=12).

G. Phase map of Per2::Luciferase bioluminescence in a representative *Prok2*$^+$/*ProkR2*$^+$ *Cry1*-initiated SCN slice showing spatial phase synchronisation across the slices (phases colour-coded relative to the mean phase of each slice).

H. Inferred neuronal group interactions in relation to circuit-level contribution as "Distributor" or "Integrator" hubs.

Data information: **C, D** unpaired two-tailed t-tests; **E** two-way ANOVA with Sidak's multiple comparisons.

1st Revision - Editorial Decision 27th Jul 2021

Dear Michael,

Thank you for submitting your revision to The EMBO journal. Your study has now been re-reviewed by referee #1. I also asked the referee to take a look at the concerns raised by referee #3. I have now heard back from the referee who appreciates the introduced changes and also your response to the concerns raised by referee #3.

I am therefore very pleased to let you know that we will accept the manuscript for publication. Before sending you the formal acceptance letter there are just a few formatting issues to resolve.

- "Declaration of Interests" should be re-labelled as Conflict of Interest

- Please check the reference format. References with more than 10 authors should be cut after 10 authors followed by et al.

- Take a look at the layout of EV1 - portrait mode might work better here.

- There is no figure call out to Fig EV2E. There are callouts to Appendix Fig S1A+B, but the figure has no panels. Appendix Figs S3+S4 panel callouts are missing.

- The appendix needs to be uploaded as one pdf file. The nomenclature needs correcting to 'Appendix Figure S#'. Please also correct callout in text

- The Reagents and Tools table should be removed from the appendix and uploaded as a file type "Reagents Table". Word or excel file is fine

- The source data needs to be split into one file per figure.

- Please make sure that the datasets are publicly available upon publication.

- Our publisher has also done their pre-publication check on your manuscript. When you log into the manuscript submission system you will see the file "Data Edited MS file". Take a look at the word file and the comments regarding the figure legends and respond to the issues.

- We include a synopsis of the paper (see http://emboj.embopress.org/). Please provide me with a general summary statement and 3-5 bullet points that capture the key findings of the paper.

- We also need a summary figure for the synopsis. The size should be 550 wide by [200-400] high (pixels). You can also use something from the figures if that is easier.

Let me know if you need any further input from me

With best wishes

Karin

Karin Dumstrei, PhD
Senior Editor

The EMBO Journal

Guide For Authors: https://www.embopress.org/page/journal/14602075/authorguide

The revision must be submitted online within 90 days; please click on the link below to submit the revision online before 25th Oct 2021.

https://emboj.msubmit.net/cgi-bin/main.plex

Referee #1:

The authors have addressed my previous comments regarding the manuscript, providing an extended of the context of their findings and clarifying some of the experimental design and analysis.

2nd Revision - Editorial Decision 9th Aug 2021

Dear Michael,

Thank you for submitting your revised manuscript to The EMBO Journal. I have looked at everything and all looks good!

I am therefore very pleased to accept the manuscript for publication here.

Congratulations on a great manuscript. I hope that this is a good reason to have a nice celebration in the lab

With best wishes

Karin

Karin Dumstrei, PhD
Senior Editor
The EMBO Journal

Please note that it is EMBO Journal policy for the transcript of the editorial process (containing referee reports and your response letter) to be published as an online supplement to each paper. If you do NOT want this, you will need to inform the Editorial Office via email immediately. More information is available here:
https://www.embopress.org/page/journal/14602075/authorguide#transparentprocess

Your manuscript will be processed for publication in the journal by EMBO Press. Manuscripts in the PDF and electronic editions of The EMBO Journal will be copy edited, and you will be provided with page proofs prior to publication. Please note that supplementary information is not included in the proofs.

Please note that you will be contacted by Wiley Author Services to complete licensing and payment information. The 'Page Charges Authorization Form' is available here:
https://www.embopress.org/pb-assets/embo-site/tej_apc.pdf

Should you be planning a Press Release on your article, please get in contact with embojournal@wiley.com as early as possible, in order to coordinate publication and release dates.

If you have any questions, please do not hesitate to call or email the Editorial Office. Thank you for your contribution to The EMBO Journal.

** Click here to be directed to your login page: https://emboj.msubmit.net

EMBO PRESS

YOU MUST COMPLETE ALL CELLS WITH A PINK BACKGROUND

PLEASE NOTE THAT THIS CHECKLIST WILL BE PUBLISHED ALONGSIDE YOUR PAPER

Corresponding Author Name: Michael Hastings
Journal Submitted to: EMBO Journal
Manuscript Number: EMBOJ-2021-108614

Reporting Checklist For Life Sciences Articles (Rev. June 2017)

This checklist is used to ensure good reporting standards and to improve the reproducibility of published results. These guidelines are consistent with the Principles and Guidelines for Reporting Preclinical Research issued by the NIH in 2014. Please follow the journal's authorship guidelines in preparing your manuscript.

A- Figures
1. Data
The data shown in figures should satisfy the following conditions:

- the data were obtained and processed according to the field's best practice and are presented to reflect the results of the experiments in an accurate and unbiased manner.
- figure panels include only data points, measurements or observations that can be compared to each other in a scientifically meaningful way.
- graphs include clearly labeled error bars for independent experiments and sample sizes. Unless justified, error bars should not be shown for technical replicates.
- if n< 5, the individual data points from each experiment should be plotted and any statistical test employed should be justified
- Source Data should be included to report the data underlying graphs. Please follow the guidelines set out in the author ship guidelines on Data Presentation.

2. Captions
Each figure caption should contain the following information, for each panel where they are relevant:

- a specification of the experimental system investigated (eg cell line, species name).
- the assay(s) and method(s) used to carry out the reported observations and measurements
- an explicit mention of the biological and chemical entity(ies) that are being measured.
- an explicit mention of the biological and chemical entity(ies) that are altered/varied/perturbed in a controlled manner.
- the exact sample size (n) for each experimental group/condition, given as a number, not a range;
- a description of the sample collection allowing the reader to understand whether the samples represent technical or biological replicates (including how many animals, litters, cultures, etc.).
- a statement of how many times the experiment shown was independently replicated in the laboratory.
- definitions of statistical methods and measures:
  - common tests, such as t-test (please specify whether paired vs. unpaired), simple χ2 tests, Wilcoxon and Mann-Whitney tests, can be unambiguously identified by name only, but more complex techniques should be described in the methods section;
  - are tests one-sided or two-sided?
  - are there adjustments for multiple comparisons?
  - exact statistical test results, e.g., P values = x but not P values < x;
  - definition of 'center values' as median or average;
  - definition of error bars as s.d. or s.e.m.

Any descriptions too long for the figure legend should be included in the methods section and/or with the source data.

In the pink boxes below, please ensure that the answers to the following questions are reported in the manuscript itself. Every question should be answered. If the question is not relevant to your research, please write NA (non applicable). We encourage you to include a specific subsection in the methods section for statistics, reagents, animal models and human subjects.

USEFUL LINKS FOR COMPLETING THIS FORM

http://www.antibodypedia.com
http://1degreebio.org
http://www.equator-network.org/reporting-guidelines/improving-bioscience-research-repor
http://grants.nih.gov/grants/olaw/olaw.htm
http://www.mrc.ac.uk/Ourresearch/Ethicsresearchguidance/Useofanimals/index.htm
http://ClinicalTrials.gov
http://www.consort-statement.org
http://www.consort-statement.org/checklists/view/32-consort/66-title
http://www.equator-network.org/reporting-guidelines/reporting-recommendations-for-tumo
http://datadryad.org
http://figshare.com
http://www.ncbi.nlm.nih.gov/gap
http://www.ebi.ac.uk/ega
http://biomodels.net/
http://biomodels.net/miriam/
http://jjj.biochem.sun.ac.za
http://oba.od.nih.gov/biosecurity/biosecurity_documents.html
http://www.selectagents.gov/

B- Statistics and general methods

Please fill out these boxes (Do not worry if you cannot see all your text once you press return)

1.a. How was the sample size chosen to ensure adequate power to detect a pre-specified effect size?	All ex vivo experiments were performed on at least five animals to ensure adequate power of detecting a pre-specificed effect size.
1.b. For animal studies, include a statement about sample size estimate even if no statistical methods were used.	Sample sizes, statistical power and variance are routinely determined in Prism 8
2. Describe inclusion/exclusion criteria if samples or animals were excluded from the analysis. Were the criteria pre-established?	Data from all the experiments were included in the analysis, with the only exlusion of SCN slices being those that died for unrelated technical reasons (e.g., inadequate seal of the glass cover on petri dishes).
3. Were any steps taken to minimize the effects of subjective bias when allocating animals/samples to treatment (e.g. randomization procedure)? If yes, please describe.	Once genotypes of animals were determined, brain tissue slices were assigned at random to treatments.
For animal studies, include a statement about randomization even if no randomization was used.	Animals were selected in an unblended manner, but no specific randomization strategy was used.
4.a. Were any steps taken to minimize the effects of subjective bias during group allocation or/and when assessing results (e.g. blinding of the investigator)? If yes please describe.	scRNASeq data was assessed blinded: only after clustering algorithms had been applied were the cell-specific barcodes back-referenced to reveal their time-of-sequencing.
4.b. For animal studies, include a statement about blinding even if no blinding was done	It was not always possible to blind experiments as genotypes of tissue slices and mice, the AAV treatments and other treatments had to be known to the investigators to ensure correct experimental design.
5. For every figure, are statistical tests justified as appropriate?	yes, assessed in Prism 8
Do the data meet the assumptions of the tests (e.g., normal distribution)? Describe any methods used to assess it.	yes, assessed in Prism 8
Is there an estimate of variation within each group of data?	yes, assessed in Prism 8

Is the variance similar between the groups that are being statistically compared?	yes, assessed in Prism 8

C- Reagents

6. To show that antibodies were profiled for use in the system under study (assay and species), provide a citation, catalog number and/or clone number, supplementary information or reference to an antibody validation profile. e.g., Antibodypedia (see link list at top right), 1DegreeBio (see link list at top right).	provided in the Appendix S3, reagents and tools table. Anti-Prok2: Cat# ab76747, RRID AB_1524238; Anti-ProkR2: Cat# APR-042; Alexa Fluor 488 Goat Anti-rabbit: Cat# A-11008, RRID AB_143165; Alexa Fluor 568 Goat Anti-rabbit: Cat# A-11011, RRID AB_2336788
7. Identify the source of cell lines and report if they were recently authenticated (e.g., by STR profiling) and tested for mycoplasma contamination.	No Cell lines used

* for all hyperlinks, please see the table at the top right of the document

D- Animal Models

8. Report species, strain, gender, age of animals and genetic modification status where applicable. Please detail housing and husbandry conditions and the source of animals.	All the experiments were performed on healthy mice, with normal immune status, housed in a specific pathogen free (SPF) unit (Ares Facility, Babraham Institute Campus, Cambridge, UK). Experimental subjects were not involved in any previous test or drug treatment. For scRNASeq and ex vivo SCN slice experiments, both female and male pups were used. Pups were maintained in a 12:12 light-dark cycle together with their mothers before being sacrificed at P10-12. SCN material used for scRNASeq at CT15-5 came from pups housed in a reversed 12:12 lighting schedule. Food and water was provided ad libitum. PER2::LUC mice (allele: B6.129S6-Per2tmlJt/J) were gifted by Joe Takahashi (UT Southwestern, US). Cryptochrome-null mice were derived from founders gifted by G van der Horst (Erasmus University Medical Centre, Rotterdam, NL). All lines were maintained on a C57BL/6J background with required genotypes bred in-house by inter-crossing the lines.
9. For experiments involving live vertebrates, include a statement of compliance with ethical regulations and identify the committee(s) approving the experiments.	All animal work was conducted and licensed in accordance with the Code of Practice for the Housing and Care of Animals Bred, Supplied or Used for Scientific Purposes under A(SP)A and the EU Directive 2010/63/EU, and with local ethical approval (MRC-LMB AWERB).
10. We recommend consulting the ARRIVE guidelines (see link list at top right) (PLoS Biol. 8(6), e1000412, 2010) to ensure that other relevant aspects of animal studies are adequately reported. See author guidelines, under 'Reporting Guidelines'. See also: NIH (see link list at top right) and MRC (see link list at top right) recommendations. Please confirm compliance.	We confirm compliance to the ARRIVE guidelines.

E- Human Subjects

11. Identify the committee(s) approving the study protocol.	NA
12. Include a statement confirming that informed consent was obtained from all subjects and that the experiments conformed to the principles set out in the WMA Declaration of Helsinki and the Department of Health and Human Services Belmont Report.	NA
13. For publication of patient photos, include a statement confirming that consent to publish was obtained.	NA
14. Report any restrictions on the availability (and/or on the use) of human data or samples.	NA
15. Report the clinical trial registration number (at ClinicalTrials.gov or equivalent), where applicable.	NA
16. For phase II and III randomized controlled trials, please refer to the CONSORT flow diagram (see link list at top right) and submit the CONSORT checklist (see link list at top right) with your submission. See author guidelines, under 'Reporting Guidelines'. Please confirm you have submitted this list.	NA
17. For tumor marker prognostic studies, we recommend that you follow the REMARK reporting guidelines (see link list at top right). See author guidelines, under 'Reporting Guidelines'. Please confirm you have followed these guidelines.	NA

F- Data Accessibility

18: Provide a "Data Availability" section at the end of the Materials & Methods, listing the accession codes for data generated in this study and deposited in a public database (e.g. RNA-Seq data: Gene Expression Omnibus GSE39462, Proteomics data: PRIDE PXD000208 etc.) Please refer to our author guidelines for 'Data Deposition'.<br><br>Data deposition in a public repository is mandatory for:<br>a. Protein, DNA and RNA sequences<br>b. Macromolecular structures<br>c. Crystallographic data for small molecules<br>d. Functional genomics data<br>e. Proteomics and molecular interactions	We confirm that a data availability section is provided. ScRNASeq data was deposited in the NCBI Gene Expression Omnibus: GSE167927. Codes used to analyse scRNASeq data are publicly available from 10X Genomics at "https://support.10xgenomics.com/single-cell-gene-expression/software/overview/welcome". Viral reagents generated in this study are to be deposited with Addgene, pProk2.Cre.T2A.mCherry and pProkR2.Cre.T2A.Venus.
19. Deposition is strongly recommended for any datasets that are central and integral to the study; please consider the journal's data policy. If no structured public repository exists for a given data type, we encourage the provision of datasets in the manuscript as a Supplementary Document (see author guidelines under 'Expanded View' or in unstructured repositories such as Dryad (see link list at top right) or Figshare (see link list at top right).	We confirm our compliance
20. Access to human clinical and genomic datasets should be provided with as few restrictions as possible while respecting ethical obligations to the patients and relevant medical and legal issues. If practically possible and compatible with the individual consent agreement used in the study, such data should be deposited in one of the major public access-controlled repositories such as dbGAP (see link list at top right) or EGA (see link list at top right).	NA
21. Computational models that are central and integral to a study should be shared without restrictions and provided in a machine-readable form. The relevant accession numbers or links should be provided. When possible, standardized format (SBML, CellML) should be used instead of scripts (e.g. MATLAB). Authors are strongly encouraged to follow the MIRIAM guidelines (see link list at top right) and deposit their model in a public database such as Biomodels (see link list at top right) or JWS Online (see link list at top right). If computer source code is provided with the paper, it should be deposited in a public repository or included in supplementary information.	We confirm our compliance

G- Dual use research of concern

22. Could your study fall under dual use research restrictions? Please check biosecurity documents (see link list at top right) and list of select agents and toxins (APHIS/CDC) (see link list at top right). According to our biosecurity guidelines, provide a statement only if it could.	
